# New indenylidene-type metathesis catalysts bearing unsymmetrical *N*-heterocyclic ligands with mesityl and nitrobenzyl substituents

**DOI:** 10.1007/s00706-016-1697-7

**Published:** 2016-03-10

**Authors:** Marta Malinowska, Mariana Kozlowska, Agnieszka Hryniewicka, Stanisław Witkowski, Jacek W. Morzycki

**Affiliations:** Institute of Chemistry, University of Białystok, Ciołkowskiego 1K, 15-245 Białystok, Poland

**Keywords:** Metathesis, Ruthenium catalyst, *N*-Heterocyclic carbenes, Nitro compounds, DFT calculations

## Abstract

**Abstract:**

New indenylidene-type second generation catalysts bearing modified unsymmetrically substituted *N*-heterocyclic carbene ligands were synthesized. The complexes contain an *N*-mesityl and *N′*-nitrobenzyl substituted NHC ligand. The precursors of free carbenes—imidazolinium salts—were obtained in an easy and environment-friendly way (under aqueous or neat conditions). The new catalysts were prepared by reaction of in situ generated carbenes with a 1st generation indenylidene catalyst, containing pyridine ligands instead of tricyclohexylphosphine. The complexes were tested in RCM, CM, and ene-yne metathesis model reactions in commercial-grade solvents in air. Their activities were compared with that of commercially available indenylidene catalyst. The structures of complexes and their stability were investigated using static DFT calculations with mixed basis set.

**Graphical abstract:**

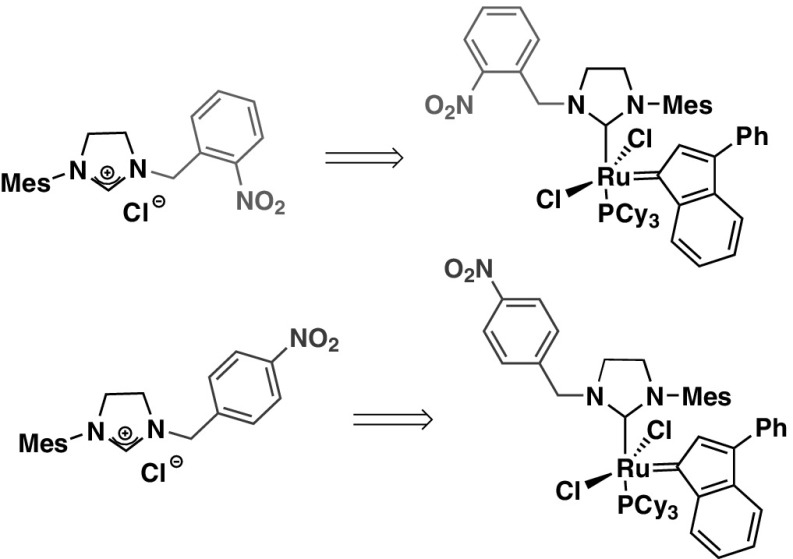

**Electronic supplementary material:**

The online version of this article (doi:10.1007/s00706-016-1697-7) contains supplementary material, which is available to authorized users.

## Introduction

**Fig. 1:**
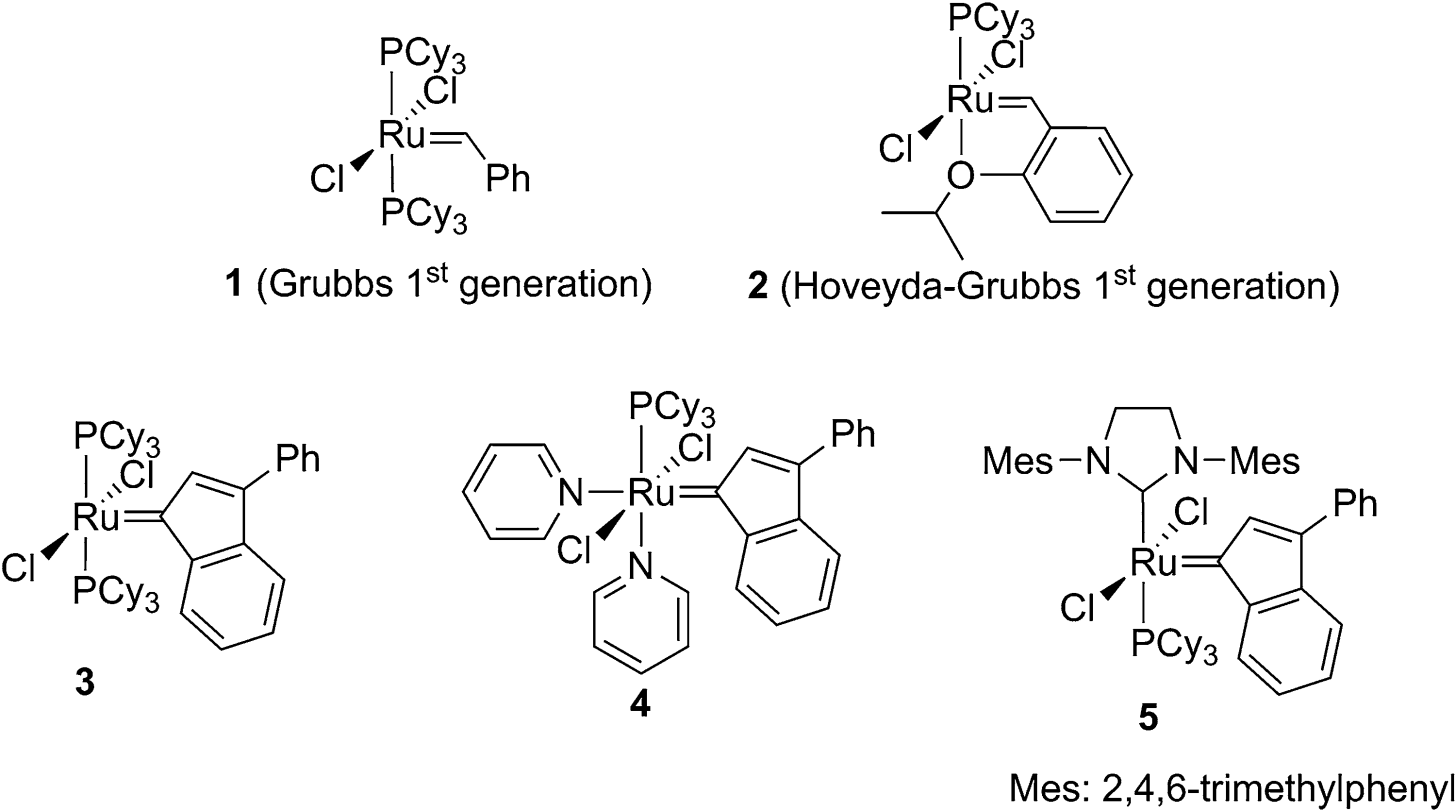
Examples of known ruthenium catalysts

Olefin metathesis is a very versatile and powerful method in organic synthesis [1]. In spite of great progress in methodology, no universal catalyst, effective in all types of metathetic transformations has been constructed yet [2–4]. Furthermore, also known complexes (Fig. 1) show numerous limitations [5, 6]. Therefore chemists in many laboratories are working on their improvement [7–9], especially to increase their activity and stereoselectivity [10]. Air-stable catalysts, that remain robust in commercial-grade solvents, are still needed. Ruthenium benzylidene complexes (e.g., **1** and **2**, Fig. 1) were shown to be less stable than indenylidene catalysts (e.g., **3**–**5**, Fig. 1) [11, 12], which are therefore attractive alternatives to well-known and more frequently used benzylidene catalysts. The indenylidene-bearing family of complexes has exhibited a rapid growth in use in recent years and is quickly becoming a mainstream catalyst in metathesis-type reactions [13].

New indenylidene-type catalysts, bearing NHC ligands with *N*-mesityl and *N′*-nitroaryl substituents were designed. We believed that unsymmetrical NHCs could affect the geometry of the metallacyclobutane intermediate produced during the reaction with an olefin, thus increasing *Z*-selectivity of the new catalyst. It was supposed that the olefin preferentially approaches the Ru-methylidene center from one side, resulting in an *all*-*cis* configuration of metallacyclobutane substituents, leading to a high *Z/E* ratio of olefinic products.

**Fig. 2:**
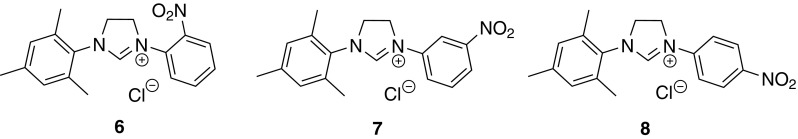
Examples of unsymmetrical imidazolinium salts

We have previously reported the synthesis of unsymmetrically substituted imidazolinium salts **6**-**8** (Fig. 2) [14]. In these salts an *ortho*-, *meta*-, or *para*-nitro substituted aromatic ring is present.

## Results and discussion

**Fig. 3:**
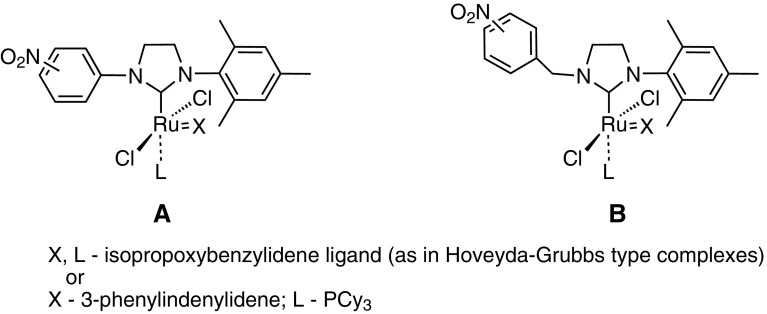
Proposed new catalysts

Two types of ruthenium catalysts with modified NHCs ligands were designed (Fig. 3). The first one (type **A**) is based on earlier reported salts **6**–**8** as the source of NHC carbene. Several attempts were undertaken to obtain new catalysts of type **A** via exchange with the commercial Hoveyda-Grubbs 1^st^ generation complex (**2**) or complex **3**. However, all experiments aimed at the NHC ligand exchange were unsuccessful. An alternative way involving the NHC carbene generation in situ by thermal decomposition of 2–(pentafluorophenyl)imidazolidine **10** in the presence of **2** was also attempted. The method also proved unsuccessful. The adduct **10** was synthesized by acid-catalyzed condensation of 2,3,4,5,6-pentafluorobenzaldehyde with diaryl substituted ethylenediamine **9**, prepared according to our earlier procedure [14] (Scheme 1).

**Figure:**
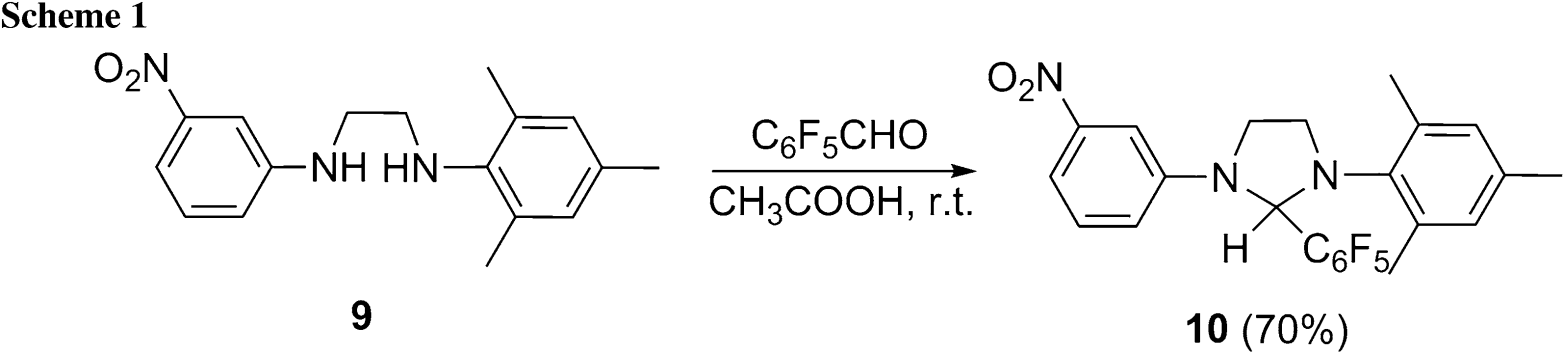

We assume that coordination of a carbene, containing the nitroaryl group directly connected with an imidazolinium ring, to the ruthenium center is not feasible. The exchange of carbene ligands in the commercial complexes failed probably due to poor nucleophilicity of the NHCs bearing nitroaryl substituents [15].

The above-described observations prompted us to design complexes with nitrobenzyl groups (type **B**, Fig. 3). A methylene spacer should effectively suppress electronic interaction with the imidazolinium ring. Separating of the electron-deficient aromatic ring from the imidazolinium part might enable coordination of the resulting carbene to the ruthenium center.

### Synthesis of new imidazolinium salts

New unsymmetrical imidazolinium salts, substituted with one nitrobenzyl group (as in the type **B** catalyst, Fig. 3) were synthesized in a simple and efficient way. *N*-Mesityl-1,2-diaminoethane (**11**) was obtained according to the modified literature procedure [16]. Condensation of **11** and *ortho*– or p*ara*-nitrobenzaldehyde under neat conditions followed by reduction of the resulting imines gave corresponding ethylenediamines in high yields. The compound **12a** was converted to dihydrochloride and subjected to cyclization with trimethyl orthoformate to give the corresponding imidazolinium salt **13a** in 77 % yield. In a similar way the compound **12b** was converted to **13b** in 80 % yield (Scheme 2).

**Figure:**
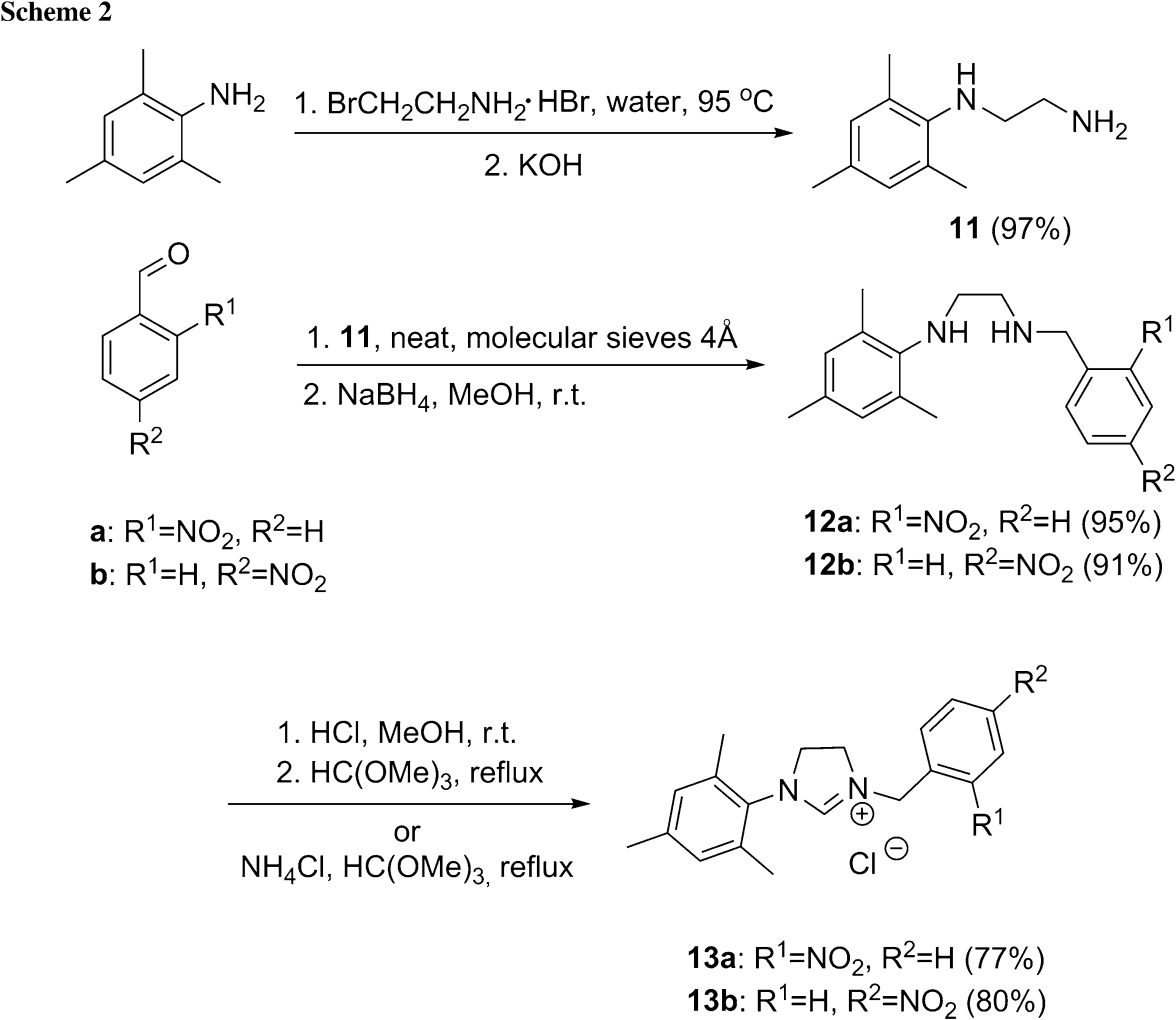

### Synthesis of new catalysts

The synthesis of new ruthenium catalysts of type **B** bearing a modified NHCs by reaction of in situ generated carbenes (from new salts **13a** and **13b**) with the commercially available indenylidene 1st generation catalyst **3** was attempted. Unfortunately, the reaction failed probably due to poor nucleophilicity of the NHCs compared to tricyclohexylphosphine ligand in the complex **3**. This issue will be the subject of further detailed investigations (e.g., including the Nolan and Cavallo method [17]). To obviate the problem the PCy_3_ ligand in **3** was first exchanged for the more labile one—pyridine [18]. A study of the obtained complex Cl_2_Ru(PCy_3_)-(pyridine)_2_(3-phenylindenylidene) (**4**) revealed that pyridine ligands can be exchanged with NHCs bearing electron-withdrawing nitro groups [15]. The bis(pyridine) adduct **4** was obtained from the catalyst **3** with an excess of pyridine as a brownish red solid and sensitive to air and moisture [19]. The carbenes, generated in situ from the imidazolinium salts **13a** and **13b** by deprotonation with potassium *tert*-butoxide, were used to synthesize new ruthenium catalysts. The complexes **14a** and **14b** were obtained in 15 and 22 % yields, respectively (Scheme 3). The attempts to increase the yields were unsuccessful.

**Figure:**
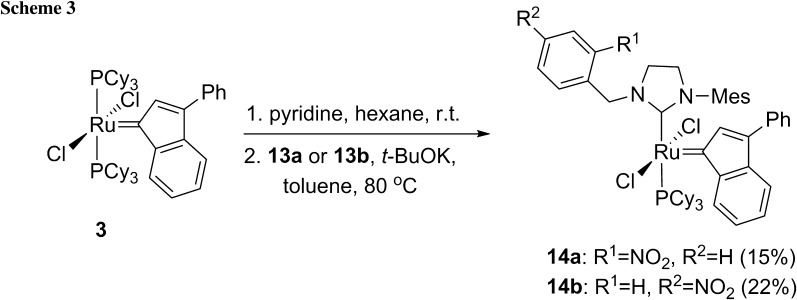

The ^1^H NMR spectrum confirmed the structure of the obtained catalysts. The signals derived from the mesityl group protons (three singlets from the methyl groups and two singlets from the aromatic protons) have been well resolved in ^1^H NMR. These signals appear also as separated singlets in the spectrum of catalyst **5** with symmetrically substituted NHC [20]. In the ^13^C NMR spectra signals from the carbenic carbons were weak. The HMBC correlation of **14a** confirmed the catalyst structure and showed good correlation of the attached proton signal from indenylidene group with the weak carbenic carbon peak.

### Testing of new catalysts

**Fig. 4:**
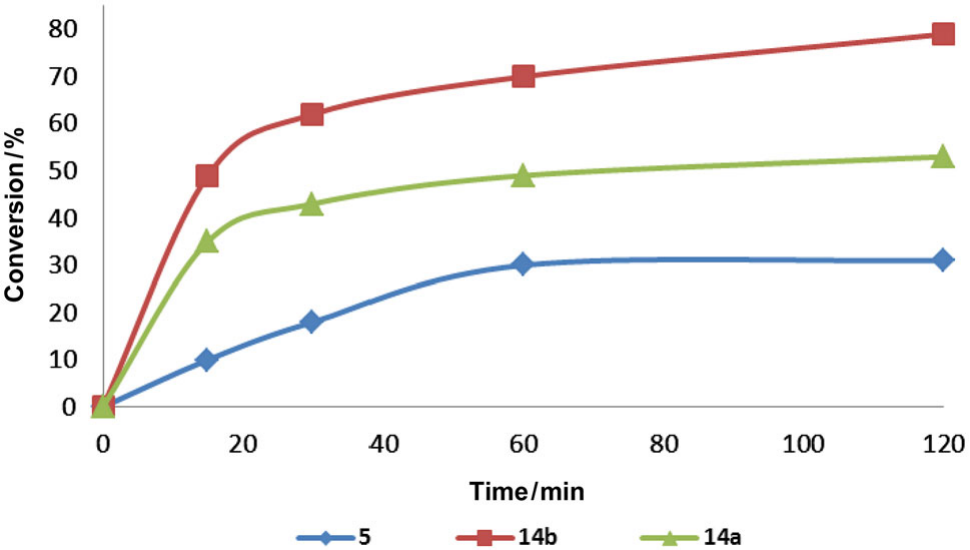
RCM of diethyl diallyl malonate in CH_2_Cl_2_ at 30 °C with 1 mol% catalyst **5**, **14a**, and **14b**. Conversion was determined by ^1^H NMR spectroscopy

The catalytic activity of the new catalysts was investigated in model RCM, CM, and ene-yne reactions using a reagent-grade non-degassed solvents in air (Table 1). The reactivity of the complexes was compared to that of **5**. Several indenylidene-type catalysts have been recently tested in air [15, 21]. The catalysts proved to be active in RCM reactions leading to di-, tri-, or tetrasubstituted olefins. Complexes **14a** and **14b** initiated RCM significantly faster than **5** (Fig. 4).

**Table 1:**
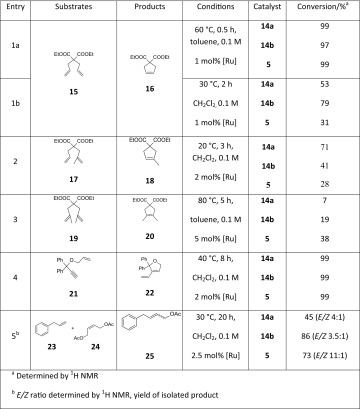
Comparative investigation of catalysts in RCM, CM and ene-yne reactions

In the RCM reaction of diethyl diallylmalonate catalysts **14a** and **14b** revealed higher reactivity than the commercial catalyst **5** when the reactions were carried out at 30 °C (Table 1, entry 1b) and comparable reactivity at elevated temperature (Table 1, entry 1a). The new complexes more efficiently promoted the formation of a trisubstituted double bond than **5**. The conversion using complex **14a** was good, while with **14b** it was only moderate (Table 1, entry 2). Surprisingly, in the RCM reactions of diethyl dimethyl allylmalonate leading to tetrasubstituted olefin using complexes **14a** and **14b** yields were lower than those for **5** (Table 1, entry 3). Although the results obtained for **5** (Table 1, entries 2 and 3) were worse than those reported under inert conditions [22, 23], they are reliable. The experiments performed simultaneously with the same batch of reagents in air clearly showed differences in reactivity of **5**, **14a**, and **14b**. The tested catalysts very effectively promoted ring-closing ene-yne metathesis (Table 1, entry 4). Catalyst **14b** demonstrated high efficiency in the cross metathesis reaction of allylbenzene with *cis*-1,4-diacetoxybut-2-ene. In this transformation **14b** gave higher yield than **5**. It should be noted that both complexes **14a** and **14b** showed approximately three times higher *Z*-selectivity than that observed for **5** (20 % *Z*-isomer for **14a**, 22 % for **14b**, and 8 % for **5**; Table 1, entry 5).

### DFT calculations

**Fig. 5:**
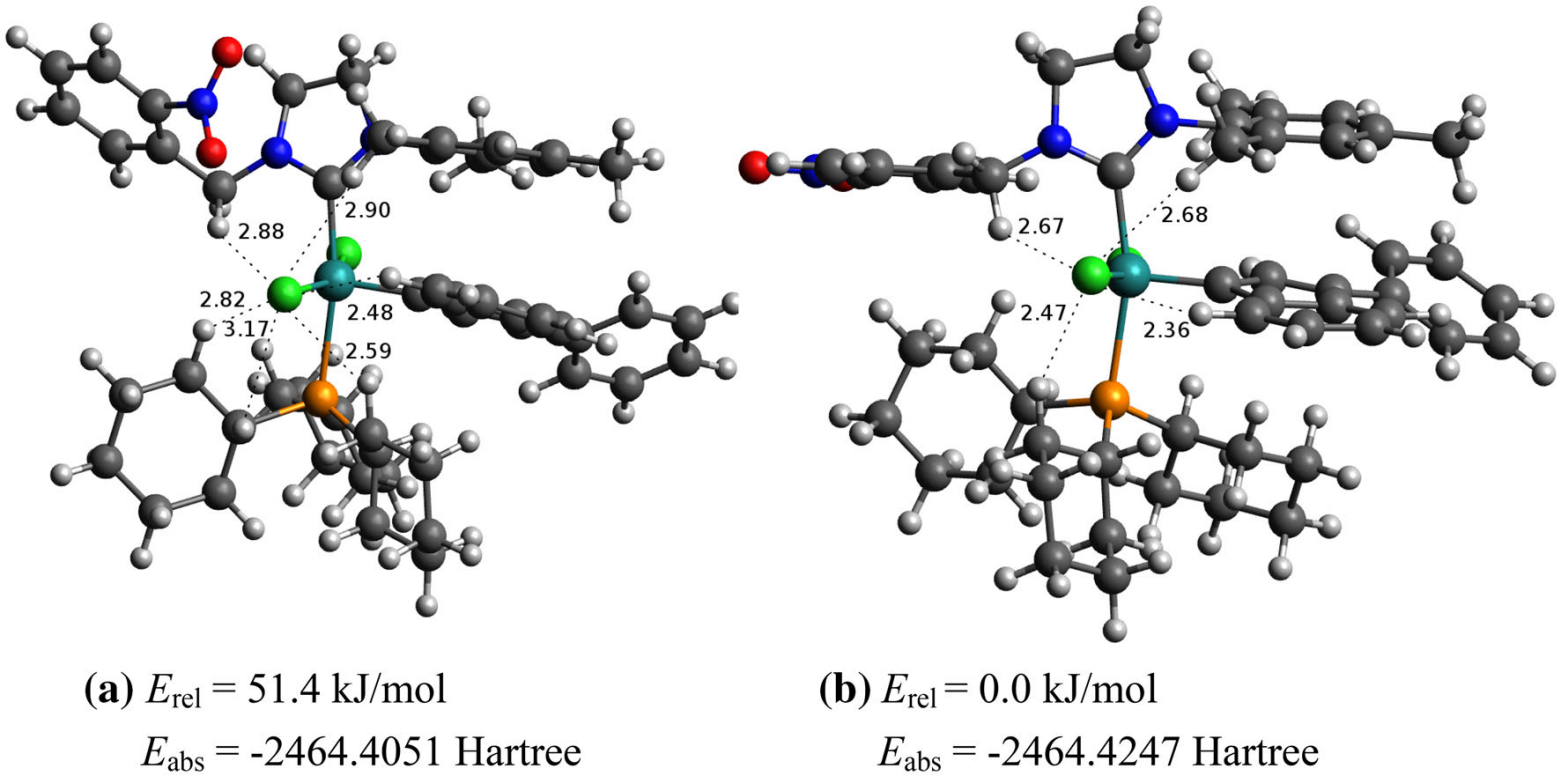
The global minima structures of **14a** (**a**) and **14b** (**b**) complexes with the corresponding values of absolute (*E*_abs_) and relative (*E*_rel_) energy obtained using B3LYP/6-311G(d,p)-LANL2DZ for P, Cl, and Ru atoms

The reactivity and *E*/*Z* selectivity of synthesized complexes **14a** and **14b** are directly connected with the geometrical parameters and additional intramolecular interactions in a particular isomer. In order to estimate the most probable structure of the indenylidene-type second generation catalysts the static DFT calculations were performed. We have considered 13 different conformers of each catalysts **14a** and **14b**. After geometry optimization some of the conformers converged to a similar local minimum or had very close absolute energies. As a result, we have chosen 6 conformers per isomer (see Supplementary Material) to show the structural diversity of the synthesized catalysts and influence of weak interactions on their catalytic activity. The global minima structures of **14a** and **14b** are shown in Fig. 5 and represent the most stable structures of complexes in the gas phase. More detailed description of these structures is available in Supplementary Material.

On the basis of computational results we have estimated the structural determinants which influence the stability and *E*/*Z* selectivity of the synthesized catalysts. Weak intramolecular interactions such as C–H···O, C–H···Cl, C–H···π hydrogen bonds and attractive π–π stacking interactions are among them. The structural parameters of the mentioned H-bonds, that denote their strength, are shown in Supplementary Material. No specific interactions between the *o*-nitro group of complex **14a** and the ruthenium atom were identified. The average Ru–P, Ru-Cl(1), and Ru-Cl(2) bond lengths amount 2.52, 2.45, and 2.55 Å for **14a** and 2.53, 2.48, and 2.54 Å for **14b**, respectively.

Higher *Z*-selectivity of the synthesized **14a** and **14b** compared to the commercial complex **5** is mainly due to the unsymmetrical NHC ligand, which induces the indenylidene ligand to be located on the side of the mesityl group, but not the nitrobenzyl one. The relative energy differences between such conformers amount about 50 and 20 kJ/mol for **14a** and **14b**, respectively (see Supplementary Material). Additionally, the movements of chlorine ligands are suppressed due to the presence of stabilizing C–H···Cl H-bonds. Therefore, olefin molecules are restricted to the nitrobenzyl side for binding to the Ru-methylidene center and therefore relatively more of a *Z*-olefinic product is formed.

## Conclusion

In summary, the synthesis of two new ruthenium indenylidene-type catalysts bearing unsymmetrical NHC ligand was described. A three-step protocol for the synthesis of unsymmetrical precursors of NHC with mesityl and nitrobenzyl substituents was elaborated. Despite initial failures in synthesis of catalysts associated with weak nucleophilicity of nitroaryl substituted NHCs, new complexes with nitrobenzyl substituted NHC were obtained. The complexes were tested in model RCM, leading to formation of five-membered cyclic products bearing di-, tri-, or tetrasubstituted double bonds, as well as ene-yne reactions. The metathesis reactions were carried out in commercial-grade solvents in air. The catalysts were also tested in a model CM reaction of allylbenzene with *cis*-1,4-diacetoxybut-2-ene showing increased *Z*-selectivity. The reactivity and higher *Z*-selectivity compared to **5** of the synthesized catalysts were explained on the basis of their conformational preferences determined by the static DFT calculations.

## Experimental

Most manipulations of organometallic compounds were carried out using standard Schlenk techniques under an atmosphere of dry argon. CH_2_Cl_2_, hexane, toluene, and chloroform were dried by distillation over CaH_2_, pyridine over KOH. Melting points were determined on a Kofler apparatus of the Boetius type. ^1^H and ^13^C NMR spectra were recorded on a Bruker Avance II spectrometer (400 and 100 MHz, respectively). Spectra are referenced relative to the chemical shift (*δ*) of TMS. Mass spectra were obtained with Micromass LCT TOF and AutoSpec Premier (Waters) spectrometers. Infrared spectra were recorded on a FT-IR spectrometer as KBr pellets or as solid samples using the ATR technique. Flash chromatography (FC) was performed on silica gel 230–400 mesh. Yields refer to chromatographically purified products unless otherwise stated. Catalysts **3** and **5** were commercially available. Substrates for testing catalysts in RCM reactions were prepared by allylation of commercial diethyl malonate with allyl bromide and/or 3-chloro-2-methylpropene according to Hensle [24]. Their purity was estimated by ^1^H NMR spectroscopy and found to be at least 95 %. Other chemicals are commercially available and used as received. *N*-Mesityl-*N*’-(2-nitrophenyl)ethylenediamine (**9**) was prepared according to literature [14].

### *N*-*Mesityl*-*N′*-*(3*-*nitrophenyl)*-*2*–*(pentafluorophenyl)imidazoline* (**10**, C_24_H_20_F_5_N_3_O_2_)

To a solution of 72 mg *N*-mesityl-*N*′-(3-nitrophenyl)ethylenediamine (0.24 mmol) in 0.5 cm^3^ AcOH 80 mg 2,3,4,5,6–pentafluorobenzaldehyde (0.41 mmol) was added and the reaction mixture was stirred for 1 h. The resulting precipitate was filtered off and washed with cold methanol to afford 80 mg (70 %) of compound **10** as a yellow solid. M.p.: 163–164 °C; ^1^H NMR (400 MHz, CDCl_3_): *δ* = 7.58 (d, 1H, *J* = 7.9 Hz, H-Ar), 7.41 (s, 1H, H-Ar), 7.32 (t, 1H, *J* = 8.2 Hz, H-Ar), 6.91 (s, 1H, H-Ar), 6.81 (s, 1H, H-Ar), 6.78 (m, 1H, H-Ar), 6.39 (s, 1H, CHC_6_F_5_), 3.99 (q, 1H, *J* = 7.9 Hz, CH_2_), 3.91 (t, 1H, *J* = 7.6 Hz, CH_2_), 3.83 (m, 1H, CH_2_), 3.65 (q, 1H, *J* = 8.0 Hz, CH_2_), 2.34 (s, 3H, CH_3_), 2.27 (s, 3H, CH_3_), 1.95 (s, 3H, CH_3_) ppm; ^13^C NMR (100 MHz, CDCl_3_): *δ* = 149.3, 144.6, 138.9, 138.0, 136.7, 136.6, 130.6, 129.9, 129.3, 117.5, 115.0, 112.0, 106.5, 70.4, 50.5, 47.1, 20.8, 18.2, 17.9 ppm; MS (ES +): *m/z* = 476 ([M-H]^+^), 430 ([M-NO_2_]^+^).

### *N*-*Mesitylethylenediamine* (**11**)

Mesitylamine (7 cm^3^, 50 mmol) and 5.13 g bromoethylamine hydrobromide (25 mmol) were vigorously stirred in 12.5 cm^3^ of water at 95 °C overnight. After cooling to room temperature, 15 cm^3^ of water was added and the solution was extracted with ethyl acetate. The aqueous phase was evaporated to dryness, the resulting solid was recrystallized from MeOH/AcOEt to give  a white powder. To the resulting product 50 cm^3^ of 20 % KOH (aq.) and CH_2_Cl_2_ were added. The residue was vigorously stirred, then separated organic layers were washed with brine, water and dried over anhydrous Na_2_SO_4_. The solvent was evaporated in vacuo to give 4.317 g of a pale yellow oil (97 %). IR, ^1^H and ^13^C NMR spectra were found to be identical with those described in literature [25].

### General procedure for the preparation of diamines 12a and 12b

An appropriate aldehyde (1 equiv.), **11** (1.5 equiv.) and molecular sieves 4 Å were stirred under Ar atmosphere overnight. Then, to the resulting mixture 10-30 cm^3^ of MeOH was added and the flask was placed in an ice-cooling bath. Next NaBH_4_ (5 equiv.) was added portionwise (three portions with 10 min intervals) and the mixture was stirred for 2 h at room temperature. The solvent was evaporated to dryness and the crude mixture was washed with saturated NaHCO_3_ (aq.) solution until pH became slightly basic. The product was extracted with ethyl acetate. Combined organic layers were washed with brine and water and dried over anhydrous Na_2_SO_4_. The solvent was evaporated in vacuo, and the obtained solid was purified by flash chromatography (hexane-AcOEt) to afford the expected product.

#### *N*-*(2*-*Nitrobenzyl)*-*N′*-*mesitylethylenediamine* (**12a**, C_18_H_23_N_3_O_2_)

General procedure was followed using 624 mg 2-nitrobenzaldehyde (4.13 mmol), 1.094 g *N*-mesityl-1,2-diaminoethane (6.2 mmol), and 785 mg NaBH_4_ (20.65 mmol). After purification (FC, hexane-AcOEt, v/v 1:1) 1.181 g (91 %) **12a** was obtained as a yellow powder. M.p.: 40.1–43 °C; IR (KBr): $\overline{v}$ = 3341, 3278, 1616, 1555, 1426, 1117 cm^−1^; ^1^H NMR (400 MHz, CDCl_3_): *δ* = 7.98 (dd, 1H, *J* = 8.2, 1.0 Hz, H-Ar), 7.65-7.59 (m, 2H, H-Ar), 7.44 (m, 1H, H-Ar), 6.84 (s, 2H, H-Ar), 4.12 (s, 2H, CH_2_), 3.09-3.07 (m, 2H, CH_2_), 2.88-2.86 (m, 2H, CH_2_), 2.65 (brs, 2H, NH), 2.29 (s, 6H, 2 × *o*-CH_3_), 2.26 (s, 3H, *p*-CH_3_) ppm; ^13^C NMR (100 MHz, CDCl_3_): *δ* = 149.2, 143.6, 135.6, 133.1, 131.2, 129.6, 129.4, 128.0, 124.8, 50.7, 49.6, 48.3, 20.6, 18.4 ppm; MS (ESI+): *m/z* = 336 ([M+Na]^+^), 314 ([M+H]^+^).

#### *N*-*(4*-*Nitrobenzyl)*-*N’*-*mesitylethylenediamine* (**12b**, C_18_H_23_N_3_O_2_)

General procedure was followed using 971 mg 4-nitrobenzaldehyde (6.43 mmol), 1.679 g *N*-mesityl-1,2-diaminoethane (9.65 mmol), and 1.222 g NaBH_4_ (32.17 mmol). After purification (FC, hexane-AcOEt, v/v 1:1) 1.913 g (95 %) **12b** was obtained as a yellow powder. M.p.: 56.1-58.6 °C; IR: $\overline{v}$ = 3350, 2914, 1604, 1515, 1340 cm^−1^; ^1^H NMR (400 MHz, CDCl_3_): *δ* = 8.39 (d, 2H, *J* = 8.7 Hz, H-Ar), 7.54 (d, 2H, *J* = 8.6 Hz, H-Ar), 6.85 (s, 2H, H-Ar), 3.97 (s, 2H, CH_2_), 3.08–3.10 (m, 2H, CH_2_), 2.86-2.89 (m, 2H, CH_2_), 2.30 (s, 6H, 2x *o*-CH_3_), 2.26 (s, 3H, *p*-CH_3_) ppm; ^13^C NMR (100 MHz, CDCl_3_): *δ* = 148.0, 147.0, 143.4, 131.2, 129.5, 129.4, 128.5, 123.5, 53.0, 49.5, 48.1, 20.4, 18.3 ppm; MS (EI +): *m/z* = 148 (MesNHCH_2_^+•^), 313 (M^+^).

#### *1*-*Mesityl*-*3*-*(2*-*nitrobenzyl)imidazolinium chloride* (**13a**, C_19_H_22_ClN_3_O_2_)

To a suspension of 1.548 g **12a** (4.95 mmol) in methanol, 1 cm^3^ of conc. HCl (9.89 mmol) was added. The solvent was evaporated, and the dihydrochloride was treated with 7.5 cm^3^ of trimethyl orthoformate. The reaction mixture was refluxed under Ar atmosphere for 2 h, and the solvent was evaporated in vacuo. The residue was dissolved in a small volume of methylene chloride, and the product was precipitated with diethyl ether to yield **13a** (1.373 g; 77 %) as a white solid. M.p.: 195–199 °C; IR: $\overline{v}$ = 3372, 2918, 1635, 1518, 1341 cm^−1^; ^1^H NMR (400 MHz, CDCl_3_): *δ* = 9.46 (s, 1H, CH), 8.33 (d, 1H, *J* = 7.7 Hz, H-Ar), 8.10 (d, 1H, *J* = 8.2 Hz, H-Ar), 7.78 (t, 1H, *J* = 7.5 Hz, H-Ar), 7.62 (t, 1H, *J* = 7.3 Hz, H-Ar), 6.94 (s, 2H, H-Ar), 5.57 (s, 2H, CH_2_), 4.33-4.27 (m, 2H, CH_2_), 4.16-4.10 (m, 2H, CH_2_), 2.32 (s, 6H, 2x *o*–CH_3_), 2.29 (s, 3H, *p*-CH_3_) ppm; ^13^C NMR (100 MHz, CDCl_3_): *δ* = 159.7, 148.5, 140.0, 135.0, 134.6, 133.4, 130.3, 129.7, 127.8, 125.2, 51.0, 48.7, 48.6, 20.8, 17.7 ppm; MS (ES+): *m/z* = 324 ([M–Cl]^+^).

#### *1*-*Mesityl*-*3*-*(4*-*nitrobenzyl)imidazolinium chloride* (**13b**, C_19_H_22_ClN_3_O_2_)

Trimethyl orthoformate (9.5 cm^3^, 82 mmol) was added to 1.280 g **12b** (4.08 mmol) and 765 mg ammonium chloride (14.3 mmol). The reaction mixture was refluxed, and the progress of reaction was monitored by TLC. After 2 h the solvent was evaporated, and the crude product was dissolved in a small volume of methylene chloride. The product **13b** (1.177 g; 80 %) was precipitated as a white solid with diethyl ether. M.p.: 267 °C; IR: $\overline{v}$ = 3383, 3038, 1632, 1518, 1337 cm^−1^; ^1^H NMR (400 MHz, CDCl_3_): *δ* = 10.37 (s, 1H, CH), 8.25 (d, 2H, *J* = 8.3 Hz, H-Ar), 7.80 (d, 2H, *J* = 8.4 Hz, H-Ar), 6.92 (s, 2H, H-Ar), 5.47 (s, 2H, CH_2_), 4.11 (s, 4H, CH_2_), 2.30 (s, 6H, 2x CH_3_), 2.28 (s, 3H, CH_3_) ppm; ^13^C NMR (100 MHz, CDCl_3_): *δ* = 160.1, 148.1, 140.4, 140.3, 134.9, 130.4, 130.3, 129.9, 124.2, 51.0, 48.3, 20.9, 18.0 ppm; MS (ES +): *m/z* = 324 ([M–Cl]^+^).

### General procedure for the preparation of catalysts 14a and 14b

The catalyst **3** (1 equiv.) was placed in a flame dried Schlenk tube and dry hexane was added under Ar atmosphere. To this suspension dry pyridine (10 equiv.) was added. The mixture was stirred 1 h at room temperature before adding dry hexane. The suspension was stored at -20 °C overnight. The resulting precipitate was filtered and washed with hexane and dried under vacuum. Next the resulting red solid, the imidazolinium salt (2 equiv.) and *t*-BuOK (2 equiv.) were placed in a flame dried Schlenk tube and dry toluene was added. The mixture was stirred for 30 min at 80 °C in a preheated oil bath. The progress of the reaction was monitored by TLC (hexane-AcOEt, v/v 7:3). After complete consumption of substrate the reaction mixture was cooled down to room temperature and the solvent was evaporated. The crude product was purified by a silica gel chromatography to afford the expected product.

#### *[[1*-*Mesityl*-*3*-*(2*-*nitrobenzyl)*-*2*-*imidazolidinylidene]dichloro*-*(3*-*phenyl*-*1H*-*inden*-*1*-*ylidene)(tricyclohexylphosphine)]ruthenium(II)* (**14a**, C_52_H_64_Cl_2_N_3_O_2_PRu)

General procedure was followed using 26 mg **3** (0.028 mmol), 20 mg salt **13a** (0.056 mmol), and 6 mg *t*-BuOK (0.056 mmol). FC (hexane-AcOEt, v/v 8:1) yielded 4 mg of a carmine solid (15 %). IR: $\overline{v}$ = 3726, 2924, 2850, 1525, 1488, 1444, 1344, 1269 cm^−1^; ^1^H NMR (400 MHz, CDCl_3_): *δ* = 8.60 (d, 1H, *J* = 6.9 Hz, H-Ar), 8.43 (d, 1H, *J* = 7.2 Hz, H-Ar), 8.01 (dd, 1H, *J* = 8.7, 1.2 Hz, H-Ar), 7.77 (m, 1H, H-Ar), 7.72 (m, 2H, H-Ar), 7.53 (m, 3H, H-Ar), 7.42 (t, 3H, *J* = 7.6 Hz, H-Ar), 7.21 (s, 1H, H-Ar), 7.17-7.24 (m, 1H, H-Ar), 7.05 (m, 1H, H-Ar), 6.43 (s, 1H, H-Ar), 6.04 (s, 2H, CH_2_), 3.74 (m, 4H, 2x CH_2_), 2.25-3.25 (m, 3H, Cy), 2.18 (s, 3H, -CH_3_), 2.06 (s, 3H, -CH_3_), 1.90 (s, 3H, -CH_3_), 1.21–1.40 (m, 13H, Cy), 1.12-1.01 (m, 7H, Cy), 0.87–0.97 (m, 10H, Cy) ppm; ^13^C NMR (100 MHz, CDCl_3_): *δ* = 291.8, 188.1, 149.8, 140.5, 137.3, 137.1, 136.8, 134.1, 133.7, 133.5, 132.1, 130.9, 129.6, 128.9, 128.6, 128.5, 128.1, 128.0, 127.7, 127.4, 127.3, 126.4, 124.2, 116.1, 52.1, 50.7, 48.8, 35.6, 35.0, 32.8, 32.7, 29.6, 29.4, 27.8, 27.7, 27.6, 27.5, 27.0, 26.9, 26.3, 26.1, 21.0, 18.5, 18.4 ppm; MS (ESI+): *m/z* = 988 ([M + Na]^+^), 930 ([M-Cl]^+^); HR-MS (ESI+): *m/z* calcd for C_52_H_64_^35^ClN_3_O_2_P^102^Ru [M-Cl]^+^ 930.3509, found 930.3468 (4.4 ppm).

#### *[[1*-*Mesityl*-*3*-*(2*-*nitrobenzyl)*-*2*-*imidazolidinylidene]dichloro*-*(3*-*phenyl*-*1H*-*inden*-*1*-*ylidene)(tricyclohexylphosphine)]ruthenium(II)* (**14b**, C_52_H_64_Cl_2_N_3_O_2_PRu)

General procedure was followed using 26 mg **3** (0.028 mmol), 20 mg salt **13b** (0.056 mmol), and 6 mg *t*-BuOK (0.056 mmol). FC (hexane-AcOEt, v/v 8:1) yielded 6 mg of a carmine solid (22 %). IR: $\overline{v}$ = 3734, 3625, 2920, 2850, 1526, 1488, 1445, 1269 cm^−1^; ^1^H NMR (400 MHz, CDCl_3_): *δ* = 8.60 (d, 1H, *J* = 7.4 Hz, H-Ar), 8.42 (d, 1H, *J* = 7.1 Hz, H-Ar), 8.01 (dd, 1H, *J* = 8.2, 1.1 Hz, H-Ar), 7.77 (m, 1H, H-Ar), 7.71 (m, 2H, H-Ar), 7.53 (m, 2H, H-Ar), 7.42 (m, 2H, H-Ar), 7.25-7.15 (m, 3H, H-Ar), 7.20 (s, 1H, H-Ar), 7.05 (m, 1H, H-Ar), 6.43 (s, 1H, H-Ar), 6.04 (s, 2H, CH_2_), 3.76-3.72 (m, 4H, 2x CH_2_), 2.37-2.32 (m, 3H, Cy), 2.18 (s, 3H, -CH_3_), 2.06 (s, 3H, -CH_3_), 1.89 (s, 3H, -CH_3_), 1.82-0.86 (m, 30H, Cy) ppm; ^13^C NMR (100 MHz, CDCl_3_): *δ* = 196.7, 149.8, 140.5, 137.1, 136.7, 133.5, 132.1, 130.9, 128.9, 128.8, 128.7, 128.5, 128.1, 127.7, 127.3, 126.4, 124.2, 116.1, 68.0, 65.7, 52.1, 50.7, 50.7, 50.1, 48.8, 48.5, 35.1, 32.8, 32.7, 30.9, 29.6, 29.5, 27.7, 27.6, 27.5, 27.0, 26.9, 26.4, 25.6, 21.0, 19.5, 18.5 ppm (Ru = C not observed); MS (ESI+): *m/z* = 988 ([M+Na]^+^).

### General RCM procedure for 15 in CH_2_Cl_2_

To a solution of alkene **15** in CH_2_Cl_2_ (0.1 M) a solution of catalyst **5**, **14a**, or **14b** (1 mol%) in CH_2_Cl_2_ was added. The resulting mixture was stirred at 30 °C for 15, 30, 60, and 120 min and controlled by TLC. The crude product was analyzed by ^1^H NMR. Spectroscopic characterization of product **16** agreed with literature data [26].

### General RCM procedure for 15 in toluene

To a solution of alkene **15** in toluene (0.1 M) a solution of catalyst **5**, **14a**, or **14b** (1 mol%) in toluene was added. The resulting mixture was stirred at 60 °C for 30 min and controlled by TLC. The crude product was analyzed by ^1^H NMR. Spectroscopic characterization of product **16** agreed with literature data [26].

### General RCM procedure for 17

To a solution of alkene **17** in CH_2_Cl_2_ (0.1 M) a solution of catalyst **5**, **14a**, or **14b** (2 mol%) in CH_2_Cl_2_ was added. The resulting mixture was stirred at 20 °C for 3 h and controlled by TLC. The crude product was analyzed by ^1^H NMR. Spectroscopic characterization of product **18** agreed with literature data [26].

### General RCM procedure for 19

To a solution of alkene **19** in toluene (0.1 M) a solution of catalyst **5**, **14a**, or **14b** (5 mol%) in toluene was added. The resulting mixture was stirred at 80 °C for 5 h and monitored by TLC. The crude product was analyzed by ^1^H NMR. Spectroscopic characterization of product **20** agreed with literature data [26].

### General ene-yne procedure for 21

To a solution of alkene in CH_2_Cl_2_ (0.1 M) a solution of catalyst **5**, **14a**, or **14b** (2 mol%) in CH_2_Cl_2_ was added. The resulting mixture was stirred at 40 °C for 8 h and monitored by TLC. The crude product was analyzed by ^1^H NMR. Spectroscopic characterization of product **20** agreed with literature data [27].

### General CM procedure for alkenes 23 and 24

To a mixture of alkene **23** (0.1 mmol, 1 equiv.) and alkene **24** (2 equiv.) in CH_2_Cl_2_ a solution of a catalyst (**5**, **14a**, or **14b**; 2.5 mol%) in CH_2_Cl_2_ was added. The resulting mixture was stirred at 30 °C for 20 h and monitored by TLC. The FC (hexane–ethyl acetate v/v 9:1) purification of the crude product yielded **25** as a colorless oil. The *E/Z* ratio was determined by ^1^H NMR. Spectroscopic characterization of product **25** agreed with literature data [28].

### Computational details

The classical density functional B3LYP [29] with a mixed basis set of the Los Alamos angular momentum projected effective core potential (ECP) using double-ζ contraction of valence functions (LANL2DZ) for ruthenium, phosphorus, chlorine [30] and 6-311G(d,p) basis set for other atoms [31] was used for geometry optimization and energy calculation in the gas phase. The geometry optimization was performed using the Broyden–Fletcher–Goldfarb–Shanno (BFGS) algorithm. The high energy and wavefunction convergence criteria of 5 × 10^−6^ and 1 × 10^−8^ Hartree, respectively, were used. The level of theory used for calculations has been recently implemented for geometry optimization in transition-metal-catalyzed reactions including olefin metathesis with Ru catalysts [32–34]. To treat dispersion interactions in catalysts the empirical dispersion correction of Grimme (D3) with the Becke–Johnson damping scheme was additionally used [35, 36]. All calculations were performed with ORCA program package (version 3.0.1) [37]. Avogadro program [38] was used to visualize structures of the examined complexes.

## Electronic supplementary material

Below is the link to the electronic supplementary material.

Supplementary material 1 (PDF 4044 kb)
