# Supplementary material for: New indenylidene-type metathesis catalysts bearing unsymmetrical N-heterocyclic ligands with mesityl and nitrobenzyl substituents
Source: Monatsh Chem. 2016 Mar 10;147:1091–100. doi: 10.1007/s00706-016-1697-7 (PMC4869738; doi:10.1007/s00706-016-1697-7)
Supplement: Supplementary file 1 — Supplementary material 1 (PDF 4044 kb) [file 706_2016_1697_MOESM1_ESM.pdf]

Supporting information

New indenylidene-type metathesis catalysts bearing unsymmetrical N-heterocyclic ligands with mesityl and nitrobenzyl substituents

Marta Malinowska*, Mariana Kozlowska, Agnieszka Hryniewicka, Stanislaw Witkowski, Jacek W. Morzycki

Institute of Chemistry, University of Bialystok, Ciolkowskiego 1K, 15-245 Bialystok, Poland

* e-mail: marta.terpilowska@gmail.com

Contents

1 Copies of NMR spectra of new compounds **2**

1.1 ^{1}H NMR spectrum of *N-mesityl-N-(3-nitrophenyl)-2(pentafluorophenyl)imidazoline* **(10)** 2

1.2 ^{13}C NMR spectrum of *N-mesityl-N-(3-nitrophenyl)-2(pentafluorophenyl)imidazoline* **(10)** 3

1.3 ^{1}H NMR spectrum of *N-(2-nitrobenzyl)-N-mesitylethylenediamine* **(12a)** 4

1.4 ^{13}C NMR spectrum of *N-(2-nitrobenzyl)-N-mesitylethylenediamine* **(12a)** 5

1.5 ^{1}H NMR spectrum of *N-(4-nitrobenzyl)-N-mesitylethylenediamine* **(12b)** 6

1.6 ^{13}C NMR spectrum of *N-(4-nitrobenzyl)-N-mesitylethylenediamine* **(12b)** 7

1.7 ^{1}H NMR spectrum of *1-Mesityl-3-(2-nitrobenzyl)imidazolinium chloride* **(13a)** 8

1.8 ^{13}C NMR spectrum of *1-Mesityl-3-(2-nitrobenzyl)imidazolinium chloride* **(13a)** 9

1.9 ^{1}H NMR spectrum of *1-Mesityl-3-(4-nitrobenzyl)imidazolinium chloride* **(13b)** 10

1.10 ^{13}C NMR spectrum of *1-Mesityl-3-(4-nitrobenzyl)imidazolinium chloride* **(13b)** 11

1.11 ^{1}H NMR spectrum of *[1-Mesityl-3-(2-nitrobenzyl)-2-imidazolidinylidene]dichloro-(3-phenyl-1H-inden-1-ylidene) (tricyclehexylphosphine)ruthenium(II)* **(14a)** 12

1.12 ^{13}C NMR spectrum of *[1-Mesityl-3-(2-nitrobenzyl)-2-imidazolidinylidene]dichloro-(3-phenyl-1H-inden-1-ylidene) (tricyclehexylphosphine)ruthenium(II)* **(14a)** 13

1.13 *HMBC correlationsbetween ^{13}C and ^{1}H NMR spectrum of [1-Mesityl-3-(2-nitrobenzyl)-2-imidazolidinylidene]dichloro-(3-phenyl-1H-inden-1-ylidene)(tricyclehexylphosphine)ruthenium(II)* **(14a)** 14

1.14 ^{1}H NMR spectrum of *[1-Mesityl-3-(2-nitrobenzyl)-2-imidazolidinylidene]dichloro-(3-phenyl-1H-inden-1-ylidene) (tricyclehexylphosphine)ruthenium(II)* **(14b)** 15

1.15 ^{13}C NMR spectrum of *[1-Mesityl-3-(2-nitrobenzyl)-2-imidazolidinylidene]dichloro-(3-phenyl-1H-inden-1-ylidene) (tricyclehexylphosphine)ruthenium(II)* **(14b)** 16

2 DFT calculations **17**

3 The final xyz coordinates of the examined indenylidene-type metathesis catalysts **22**

1. Copies of NMR spectra of new compounds

1.1. ^{1}H NMR spectrum of N-mesityl-N-(3-nitrophenyl)-2(pentafluorophenyl)imidazoline **(10)**

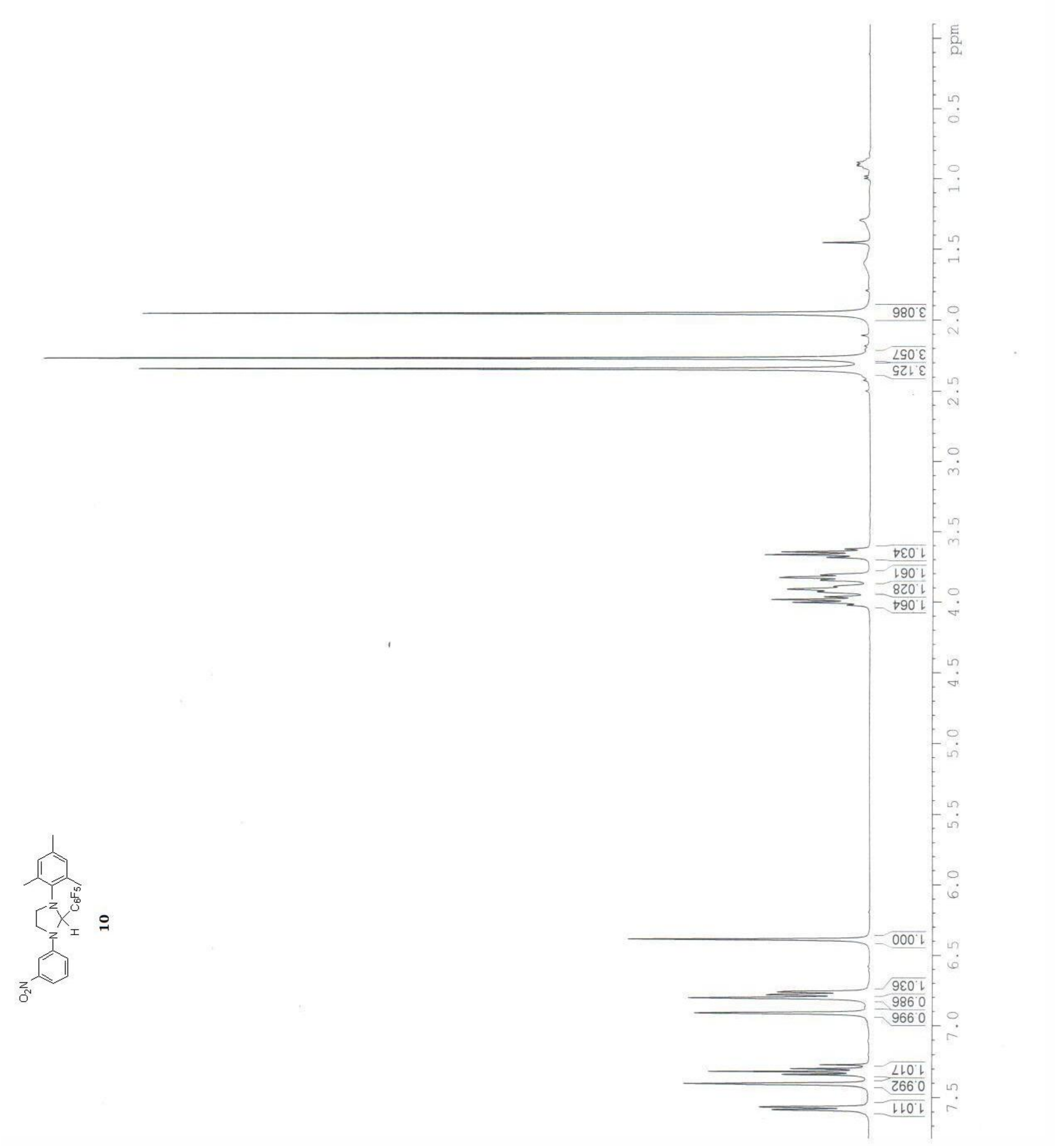

1.2. ^{13}C NMR spectrum of N-mesityl-N-(3-nitrophenyl)-2(pentafluorophenyl)imidazoline **(10)**

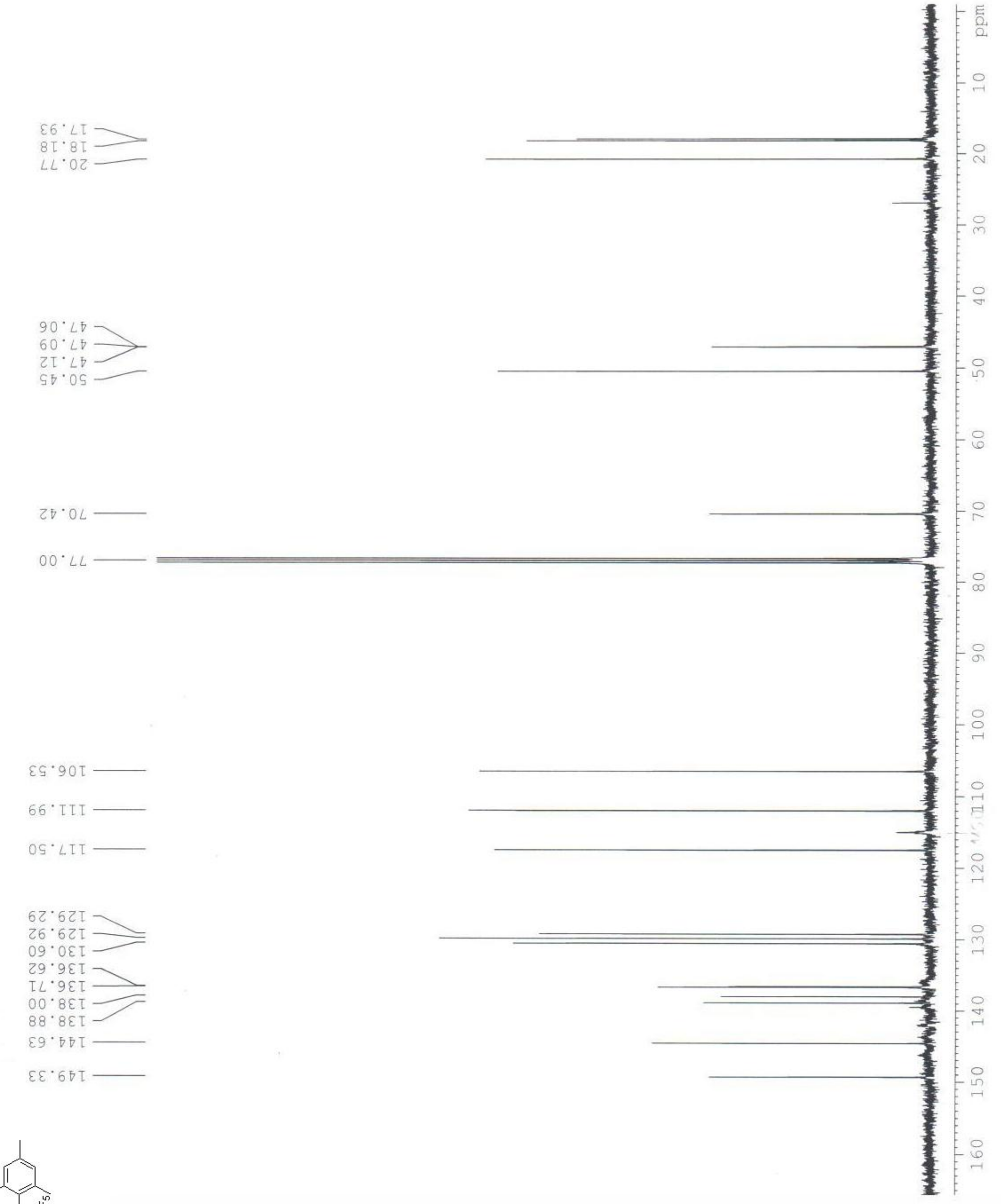

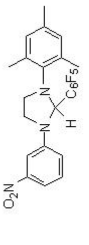

10

1.3. ^{1}H NMR spectrum of N-(2-nitrobenzyl)-N-mesitylethylenediamine **(12a)**

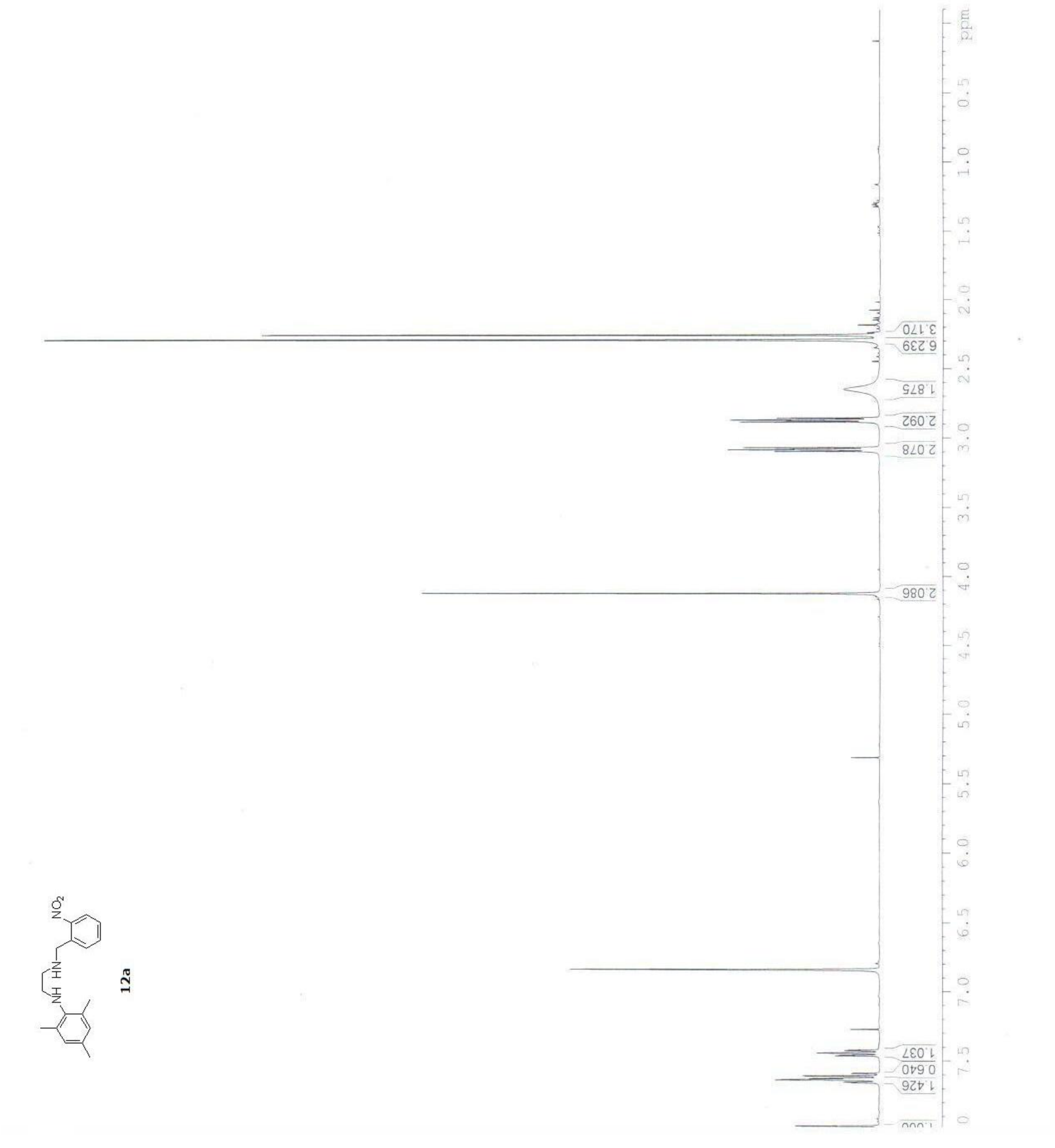

1.4. ^{13}C NMR spectrum of N-(2-nitrobenzyl)-N-mesitylethylenediamine **(12a)**

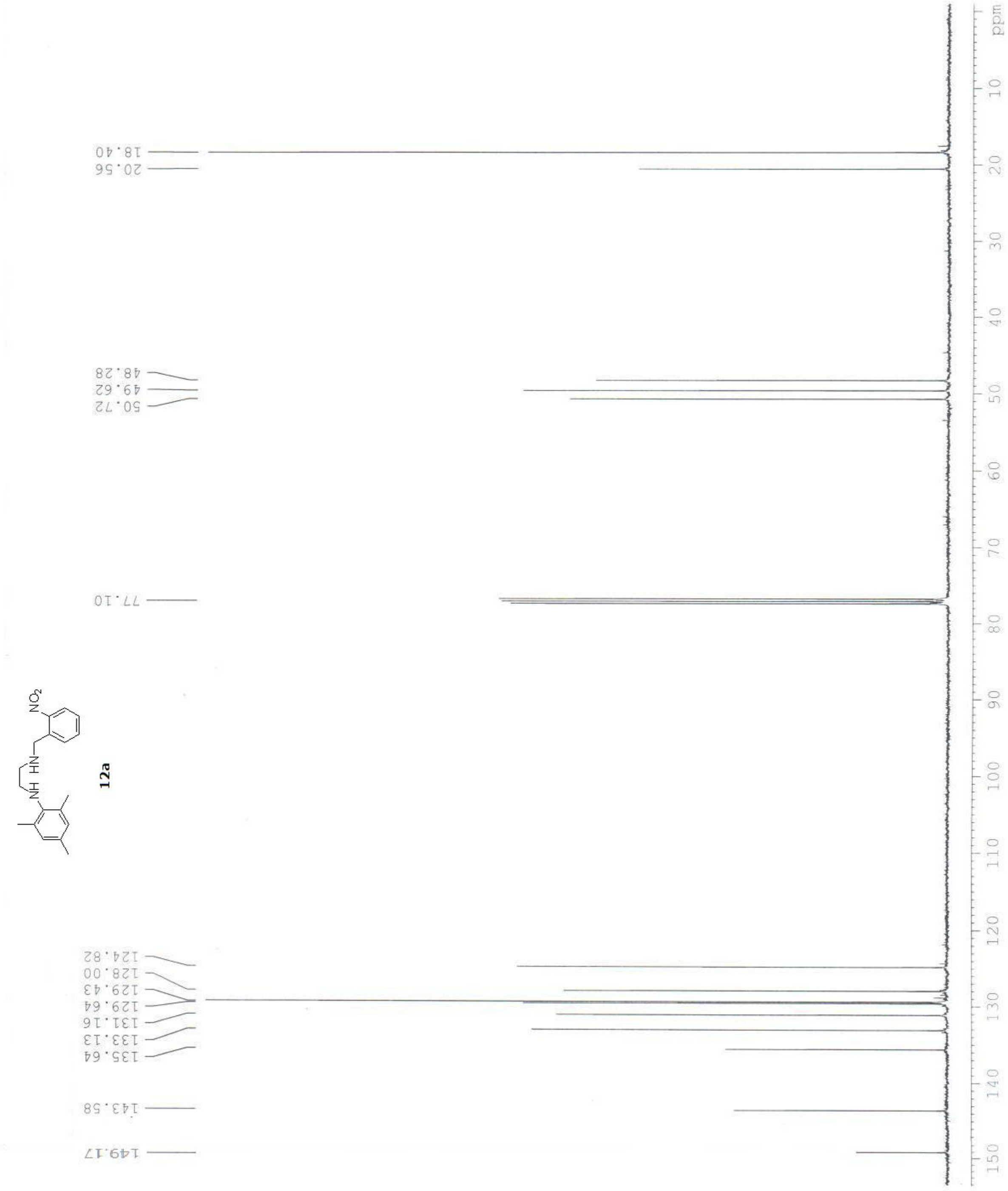

1.5. ^{1}H NMR spectrum of N-(4-nitrobenzyl)-N-mesitylethylenediamine **(12b)**

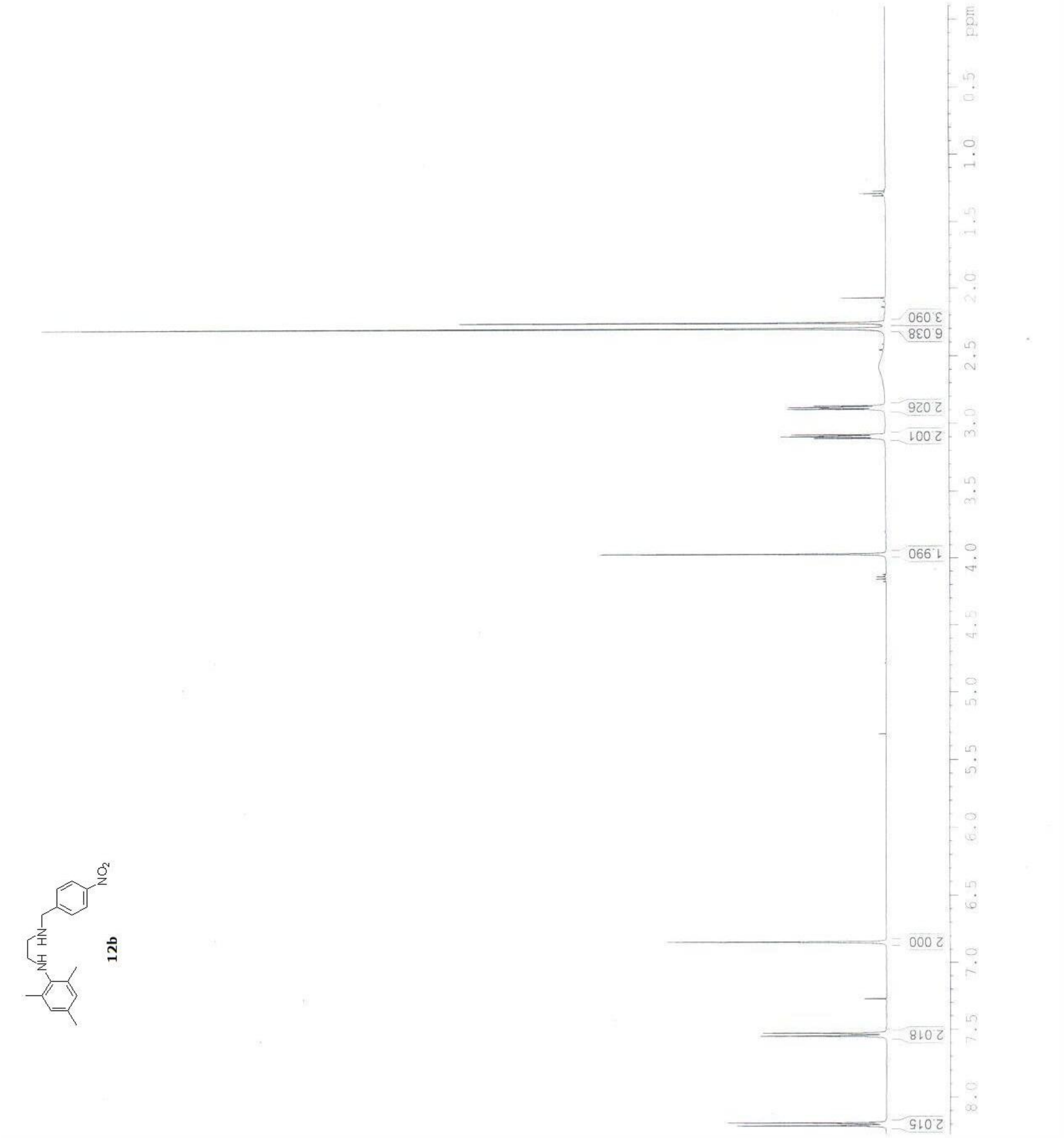

1.6. ^{13}*C NMR spectrum of N-(4-nitrobenzyl)-N-mesitylethylenediamine* **(12b)**

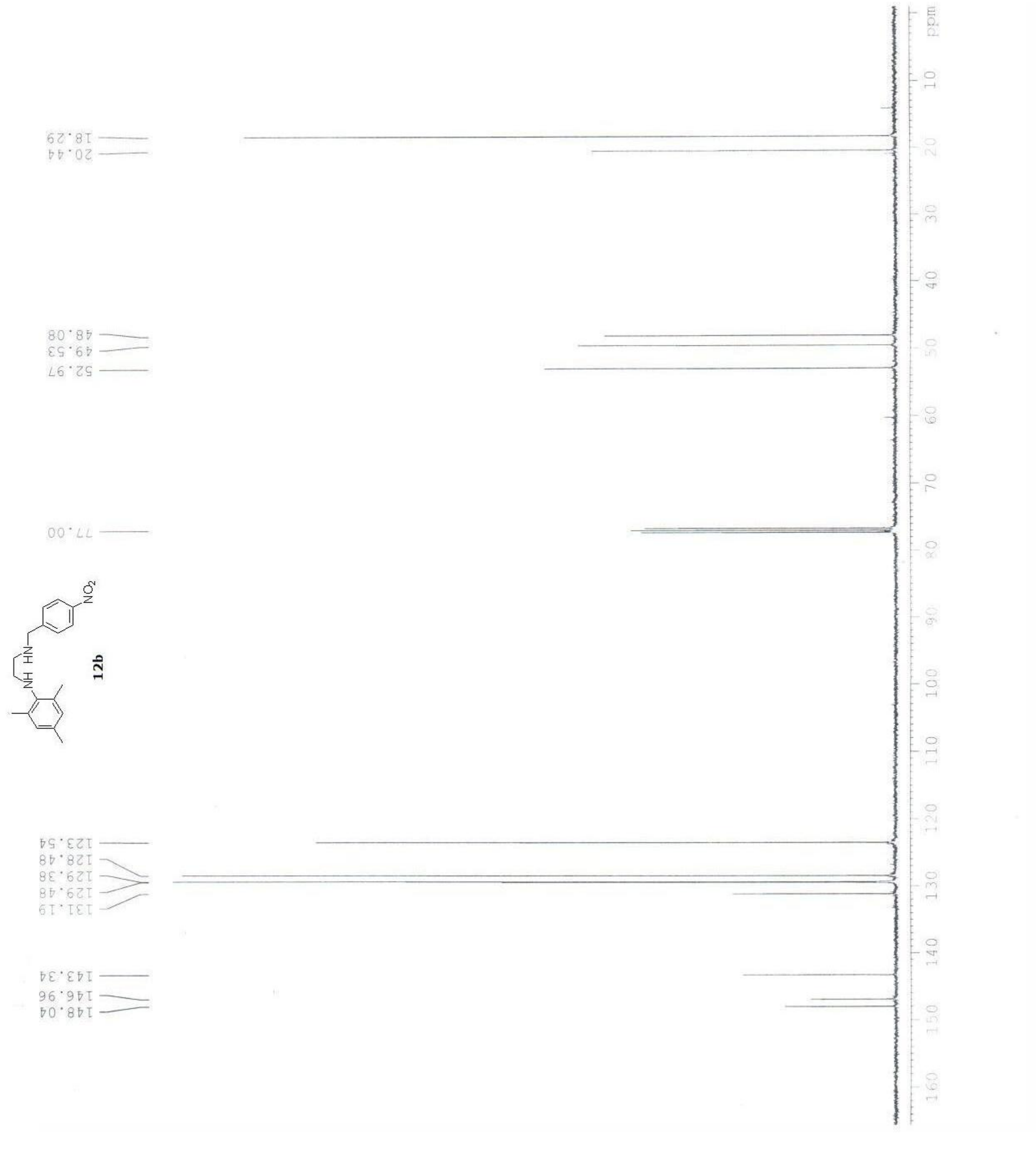

1.7. ^{1}H NMR spectrum of 1-Mesityl-3-(2-nitrobenzyl)imidazolinium chloride **(13a)**

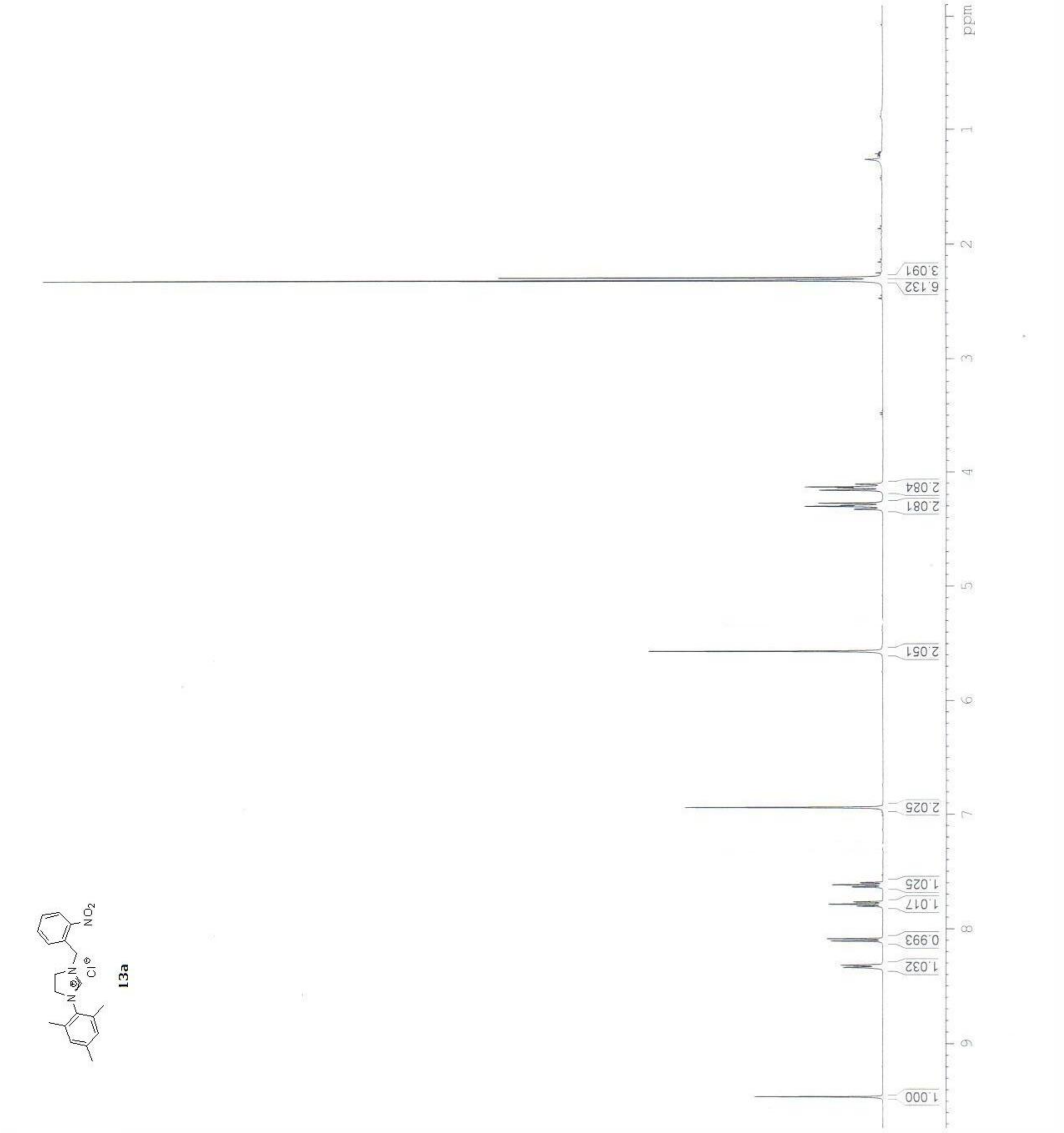

1.8. ^{13}C NMR spectrum of 1-Mesityl-3-(2-nitrobenzyl)imidazolinium chloride **(13a)**

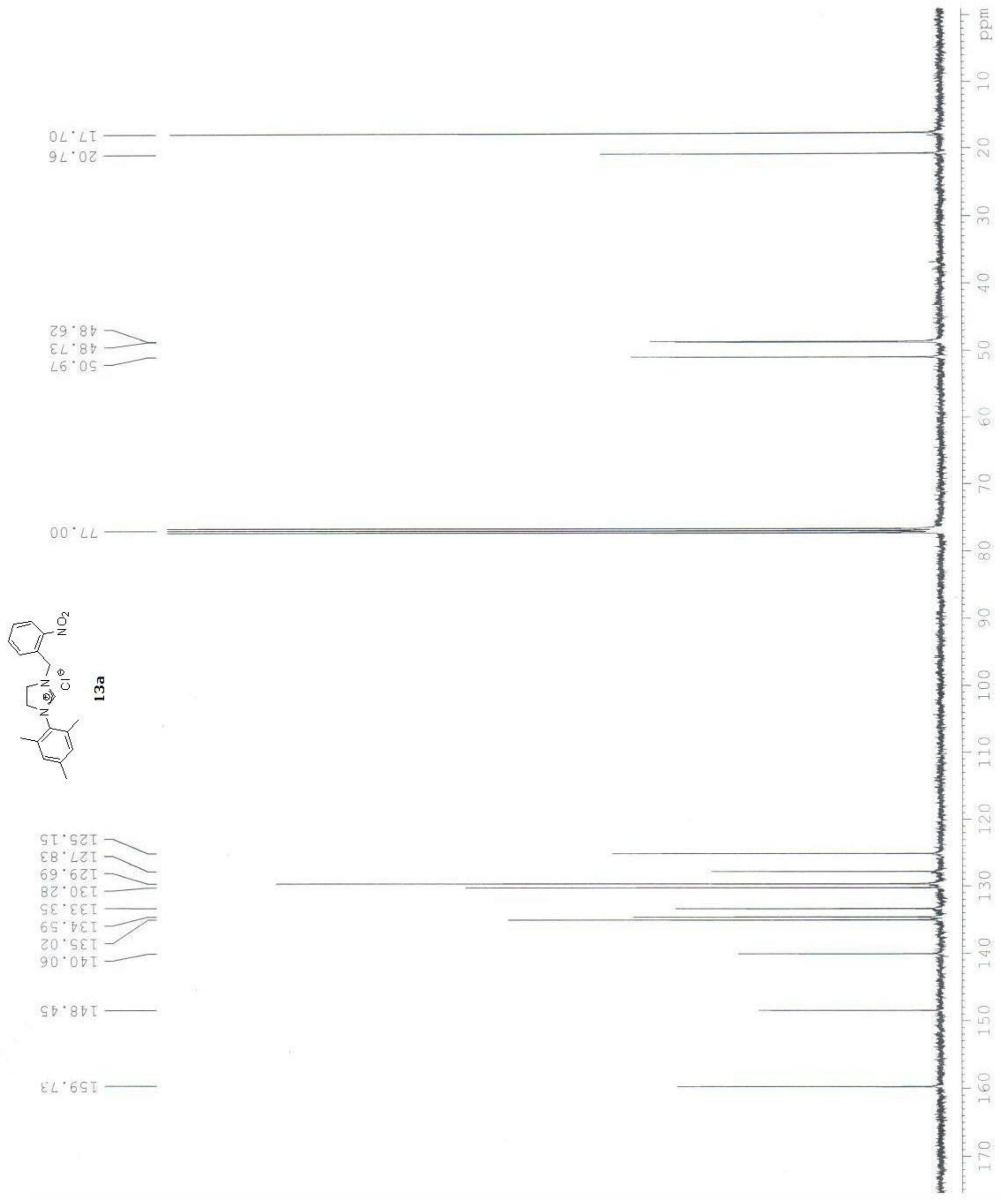

1.9. ^{1}H NMR spectrum of 1-Mesityl-3-(4-nitrobenzyl)imidazolinium chloride **(13b)**

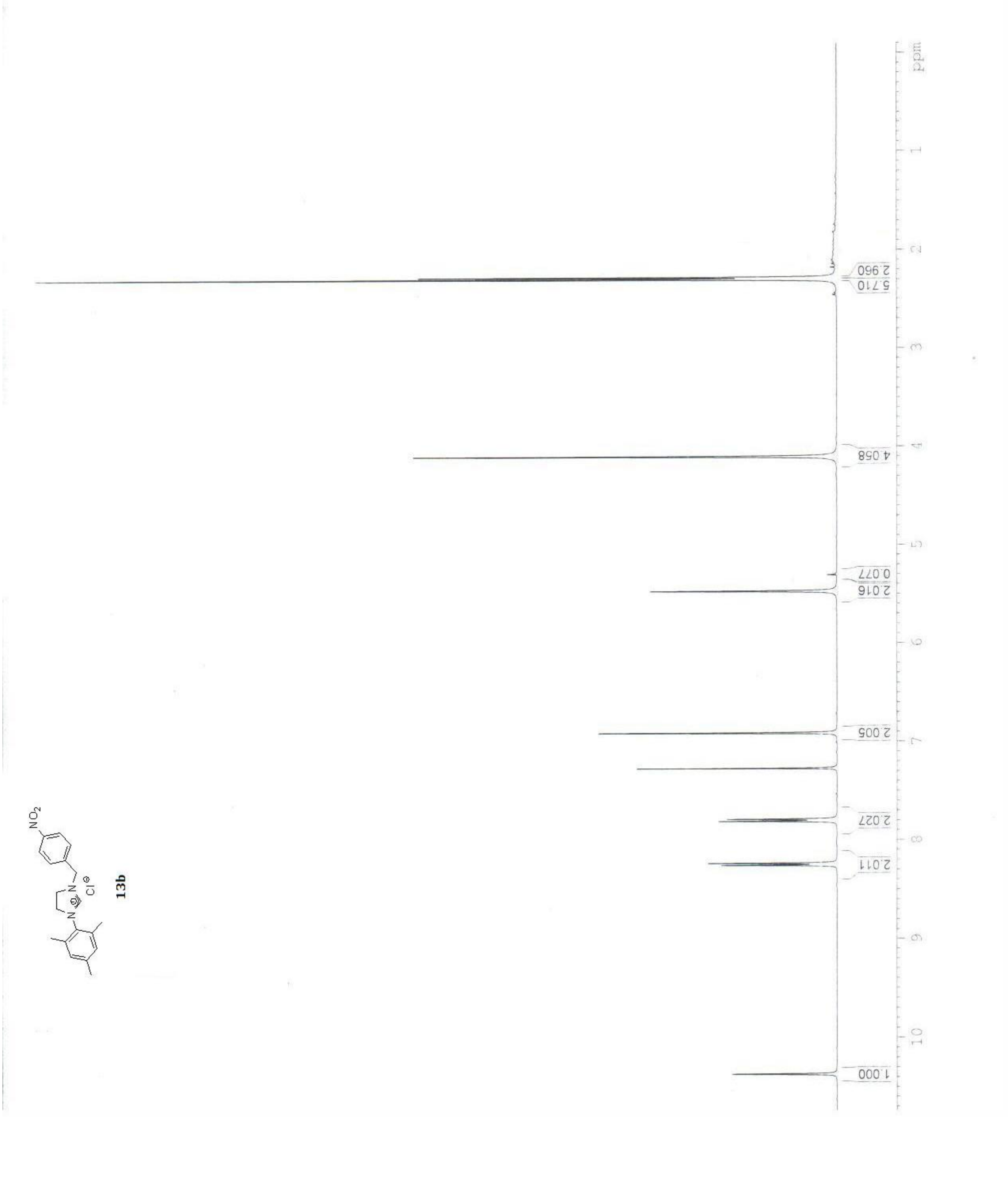

1.10. ^{13}C NMR spectrum of 1-Mesityl-3-(4-nitrobenzyl)imidazolinium chloride **(13b)**

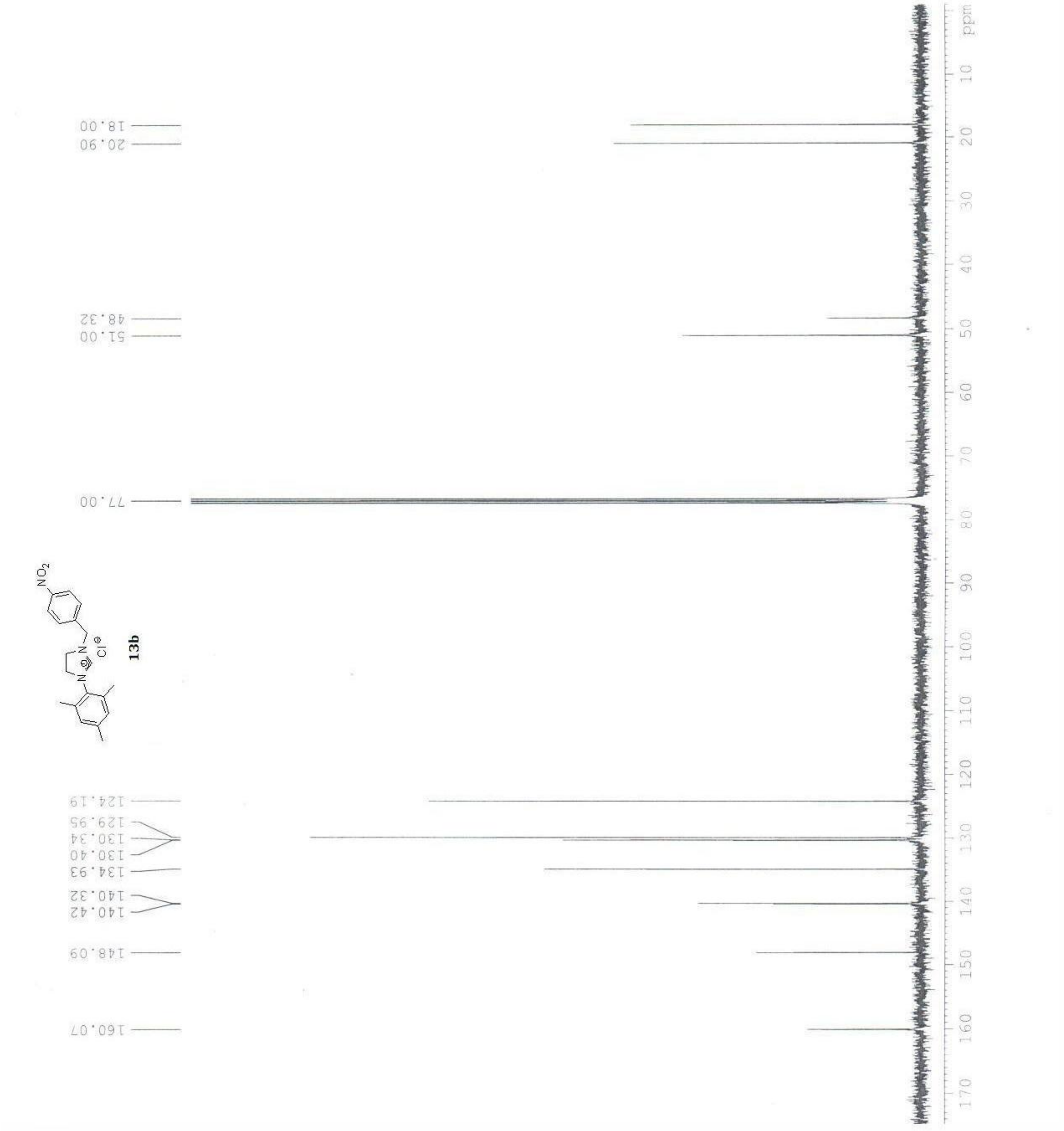

1.11. [1]*H NMR spectrum of [1-Mesityl-3-(2-nitrobenzyl)-2-imidazolidinylidene]dichloro-(3-phenyl-1H-inden-1-ylidene) (tricyclehexylphosphine)ruthenium(II)* **(14a)**

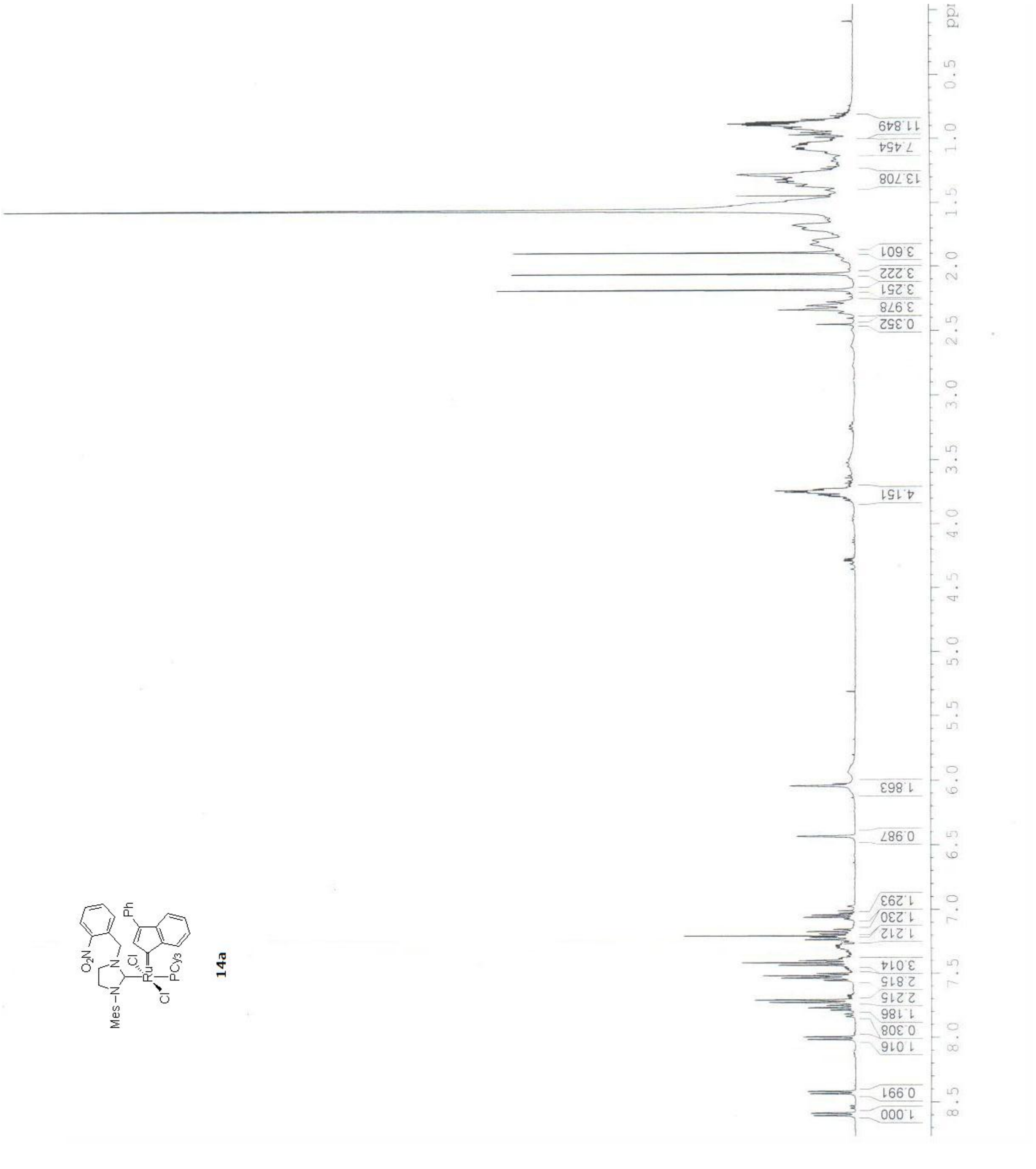

14a

1.12. ^{13}*C NMR spectrum of [1-Mesityl-3-(2-nitrobenzyl)-2-imidazolidinylidene]dichloro-(3-phenyl-1H-inden-1-ylidene) (tricyclehexylphosphine)ruthenium(II)* **(14a)**

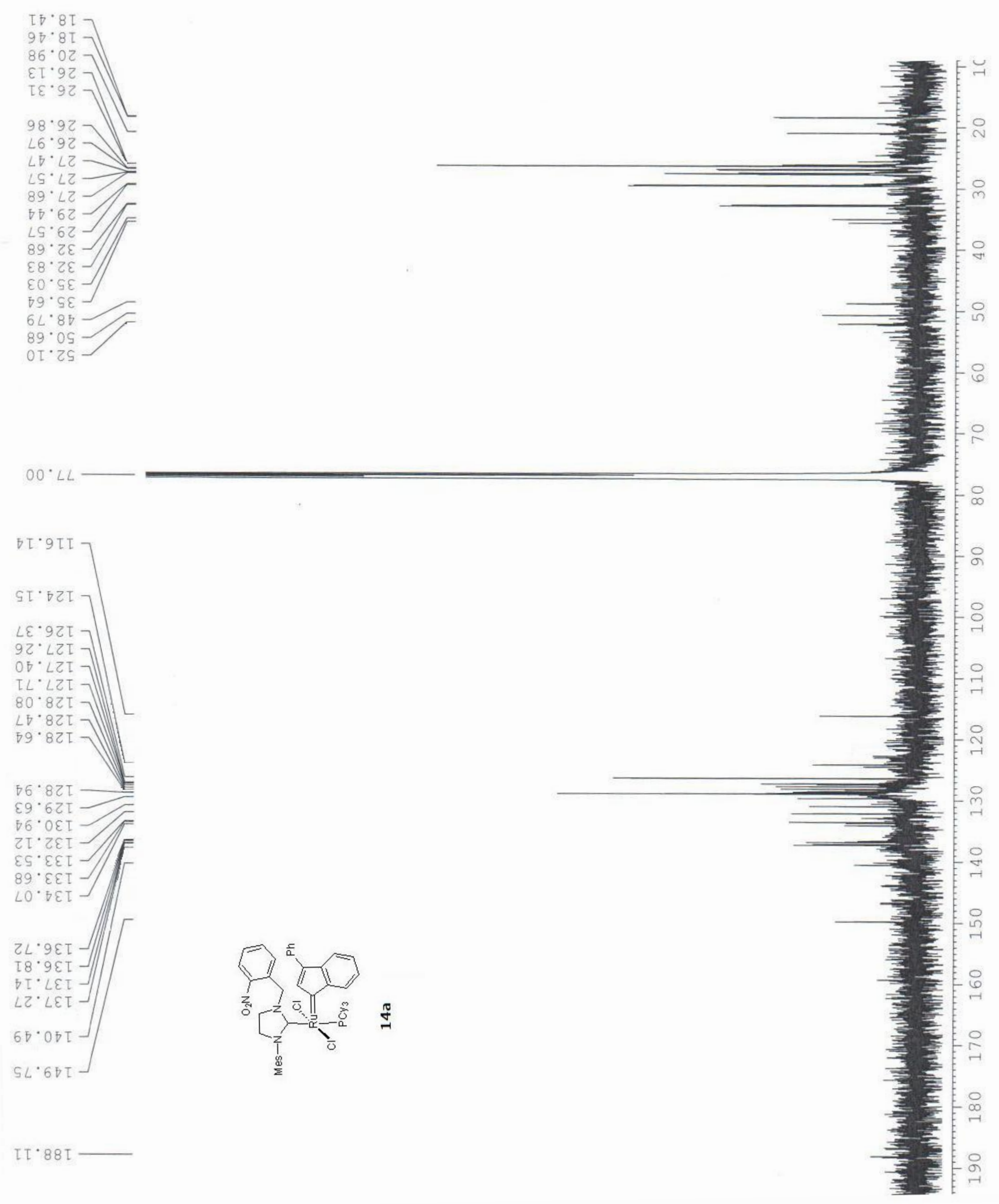

1.13. HMBC correlationsbetween ^{13}C and ^{1}H NMR spectrum of [1-Mesityl-3-(2-nitrobenzyl)-2-imidazolidiny-lidene]dichloro-(3-phenyl-1H-inden-1-ylidene)(tricyclehexylphosphine)ruthenium(II) **(14a)**

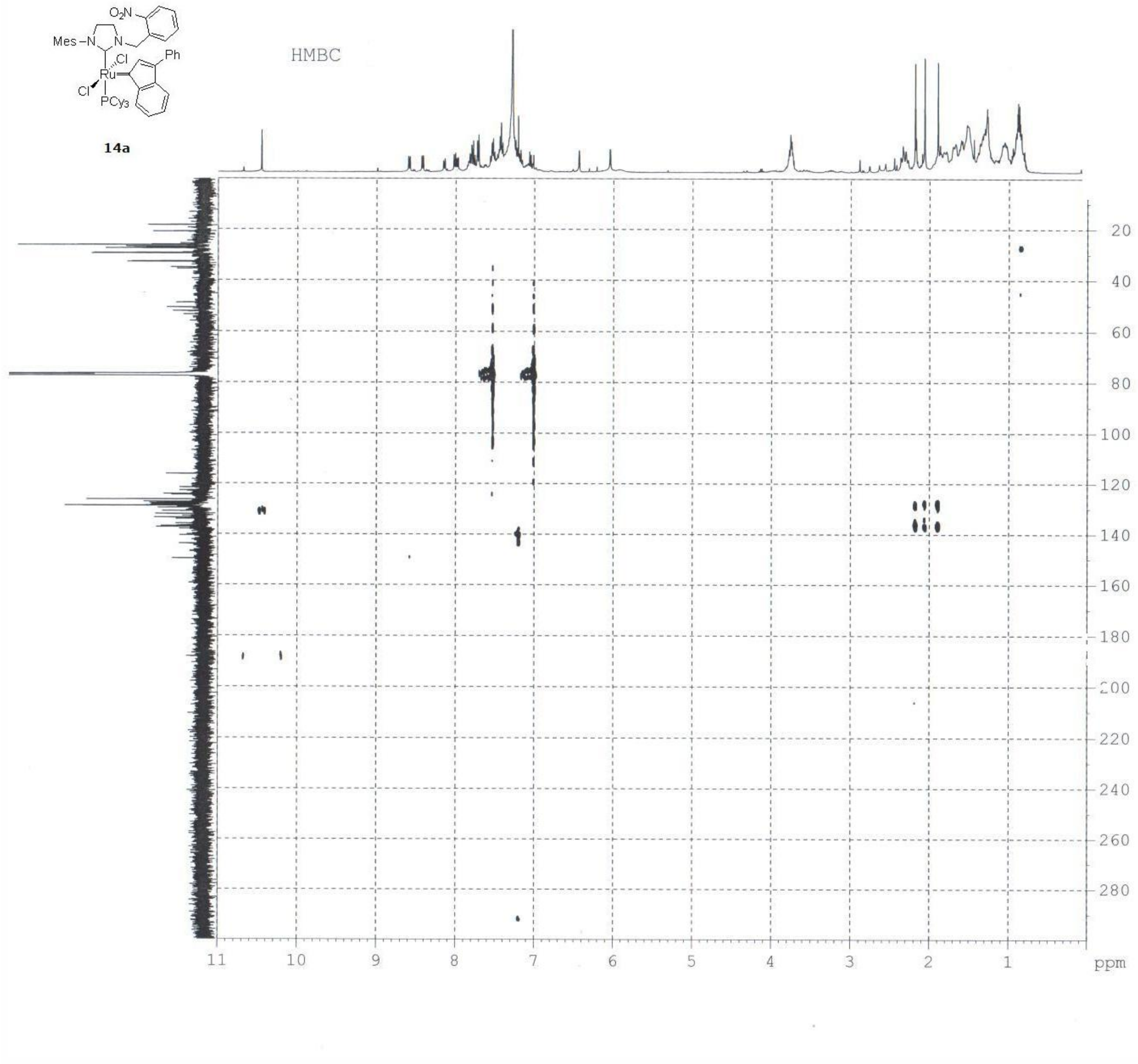

1.14. [1]*H NMR spectrum of [1-Mesityl-3-(2-nitrobenzyl)-2-imidazolidinylidene]dichloro-(3-phenyl-1H-inden-1-ylidene) (tricyclehexylphosphine)ruthenium(II)* **(14b)**

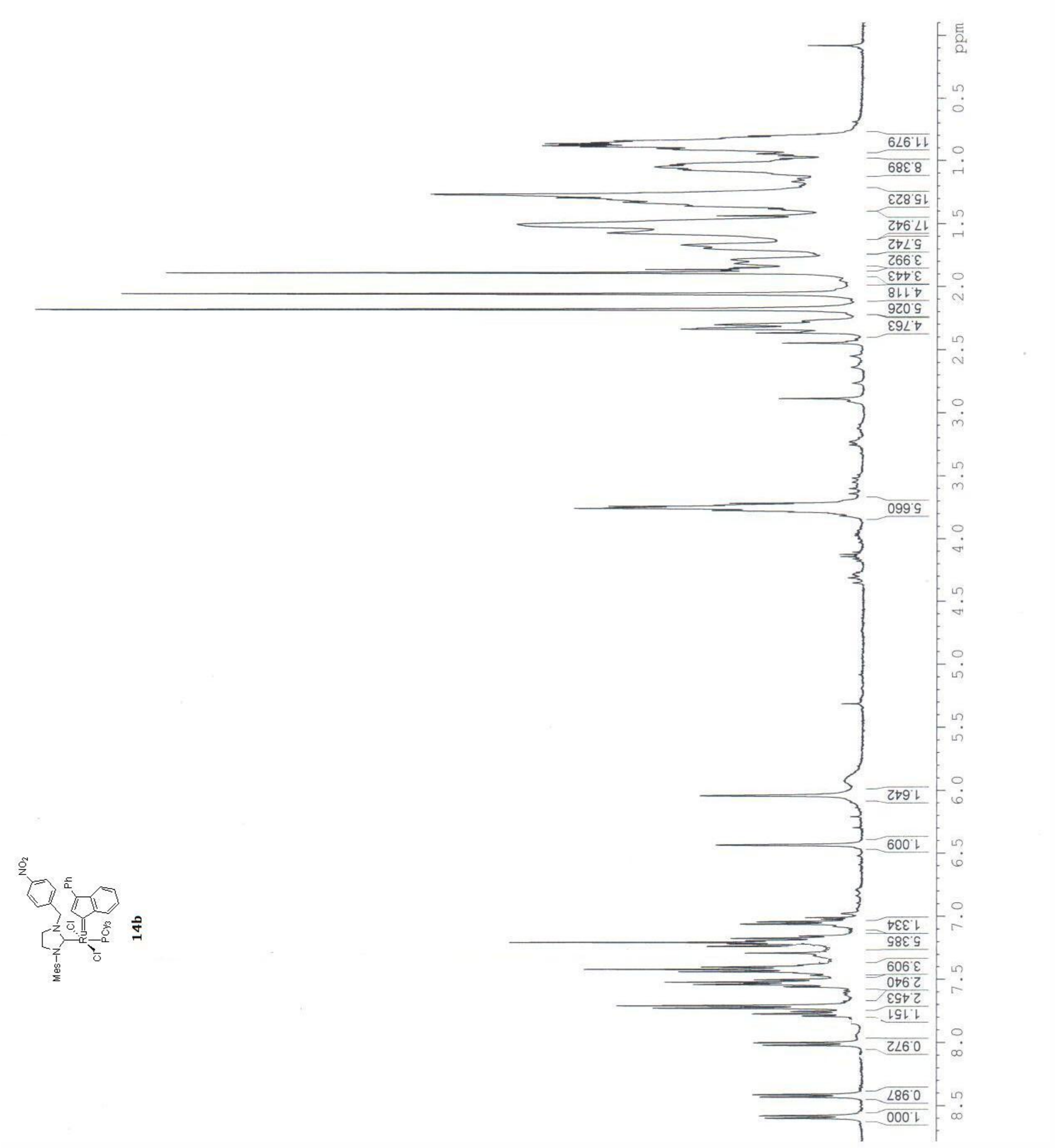

1.15. ^{13}C NMR spectrum of [1-Mesityl-3-(2-nitrobenzyl)-2-imidazolidinylidene]dichloro-(3-phenyl-1H-inden-1-ylidene) (tricyclehexylphosphine)ruthenium(II) **(14b)**

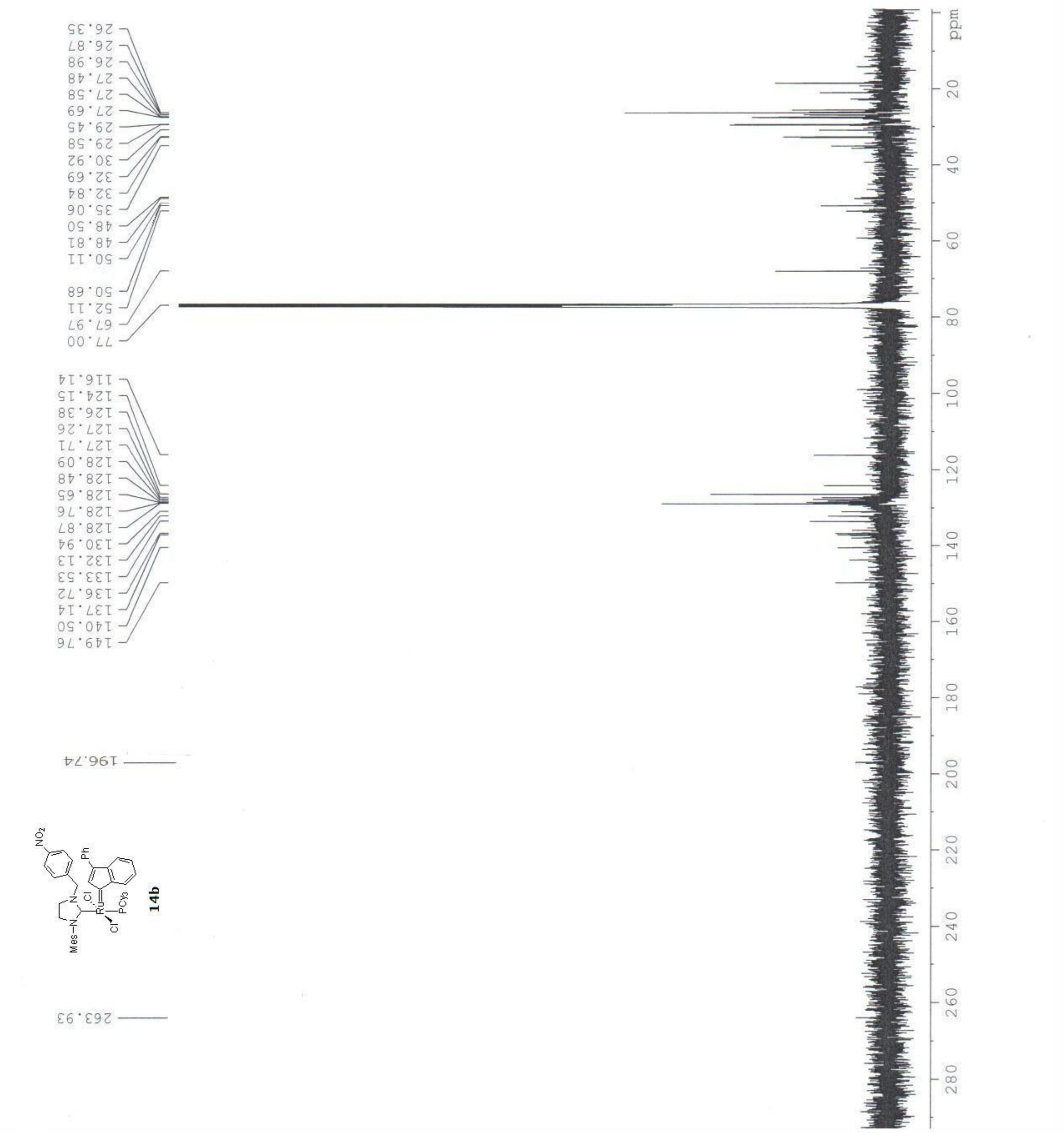

2. DFT calculations

To estimate the most stable structures of new indenylidene-type metathesis catalysts we performed geometry optimization of 13 conformers of each complex. Our main goal was to appoint additional intramolecular interactions which impact the stability of the examined ruthenium complexes. Therefore, we modified the structures of catalysts changing the possibility for hydrogen bonds formation, $\pi-\pi$ stacking interactions and specific $NO_2\cdots$Ru interactions. The used computational setup for all performed calculations is included in the manuscript.

Due to the fact that some of the calculated conformers converged to the same local minima or had very similar values of absolute energy we have chosen only 6 conformers for further analysis. Fig. 1 and Fig. 2 contain the selected local minima after the geometry optimization of **14a,b**. The corresponding relative energy value for all local minima are also given in the figures. The relative energy value is the difference between the values of absolute energy for the particular local minimum and the global minimum, respectively. The higher is the value of relative energy the less stable is the structure of the complex. The most stable structure – the global minimum – is marked with the relative energy equal to 0.0. kJ/mol.

Two synthesized ruthenium complexes differs with the position of the nitro group in the phenyl ring bonded to the *N*-heterocyclic carbene ligand. The comparative analysis of their global minima indicated that complex **14b**, with the nitro group in *para-* position, is more stable than the complex **14a**, with the nitro group linked in *ortho-* position. The value of their absolute energy difference is 51.4 kJ/mol, which is caused with stronger C-H$\cdots\pi$ interactions and C-H$\cdots$Cl hydrogen bonds in the case of **14b** catalyst. The detailed structural parameters of all C-H$\cdots$Cl H-bonds formed in the examined structures are listed in Table 1 and Table 2. Even if C-H$\cdots$Cl H-bonds belong to the weak H-bonds, their amount in the case of synthesized catalysts is enough to influence the structural stability of **14a,b**. The structure **14a(1)** has additional weak C-H$\cdots$O H-bonds formed between the H atom from the mesityl group as the proton donor and the O atom from the nitro group as the proton acceptor. The structural parameters of the mentioned C-H$\cdots$O H-bonds in **14a(1)** equal to 3.01 Å, 3.08 Å and 140.56°, 119.98° for the H$\cdots$O distance and $\angle$ CHO, respectively. Such H-bonds are too weak to stabilize efficienly the structure. Therefore, their absence does not influence the stability of the structure **14b(1)**, which is more stable than **14a(1)**.

Among other specific intramolecular interactions in the synthesized ruthenium catalysts are C-H$\cdots\pi$ H-bonds and $\pi-\pi$ stacking interactions. In the case of complex **14b** the nitro group is located far from mesityl group allowing it to locate in the preferable position for $\pi-\pi$ stacking interactions and C-H$\cdots\pi$ H-bonds formation with the indenylidene ligand. As the result, the energy gain of such interactions is higher in the case of **14b** than **14a**. Moreover, the preferable position of the indenylidene ligand is on the side of mesityl group (like in **14b(1)**), but not on the side of nitrobenzyl one (**14b(2)**), see Fig 2. Similar behavior can be also seen from the structures **14a(1)** and **14a(4)** in Fig 1. Such specific arrangement of indenylidene ligand causes better Z-selectivity of the synthesized complexes comparing with the commercial one (**5**). In all examined complexes no interactions between nitro group and the ruthenium atom were identified.

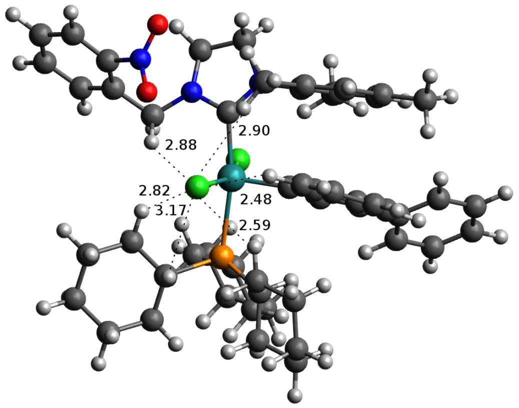

14a(1): E$_{rel}$ = 0.0 kJ / mol

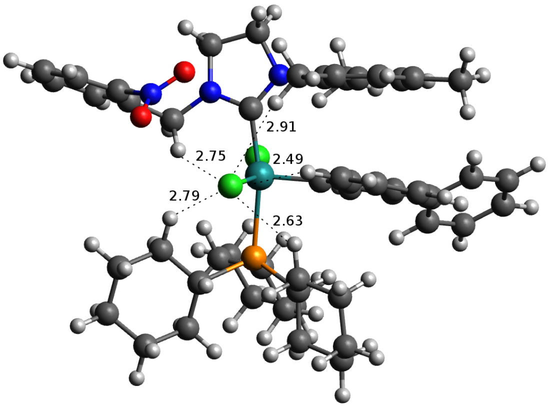

14a(2): E$_{rel}$ = 6.0 kJ / mol

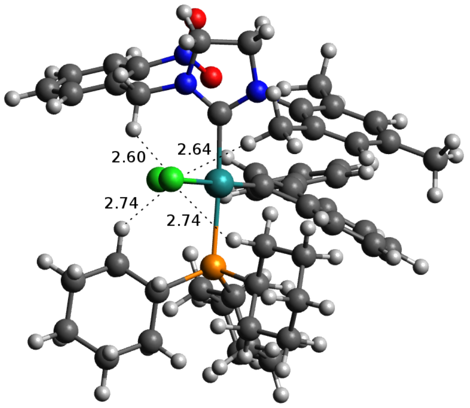

14a(3): E$_{rel}$ = 40.6 kJ / mol

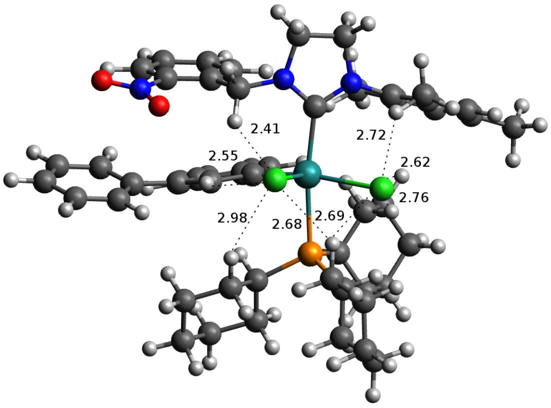

14a(4): E$_{rel}$ = 58.3 kJ / mol

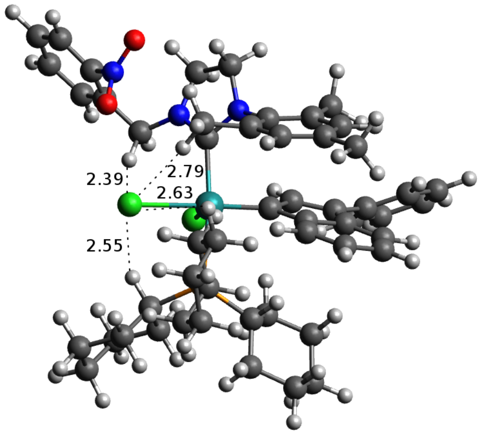

14a(5): E$_{rel}$ = 69.9 kJ / mol

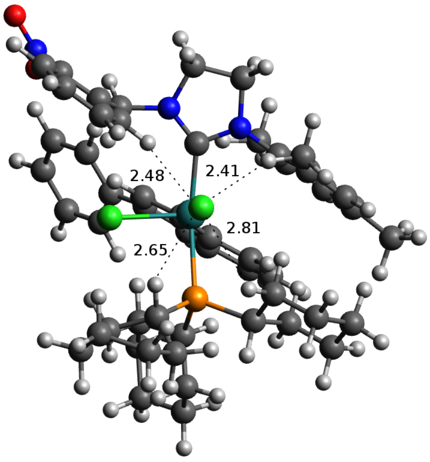

14a(6): E$_{rel}$ = 82.1 kJ / mol

Figure 1: Selected structures of the optimized local minima of **14a** complex with the corresponding relative energy value.

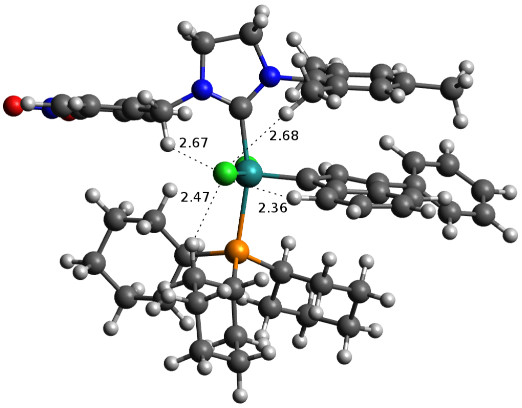

14b(1): E$_{rel}$ = 0.0 kJ / mol

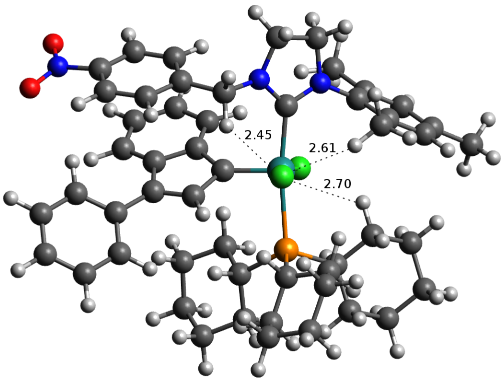

14b(2): E$_{rel}$ = 17.7 kJ / mol

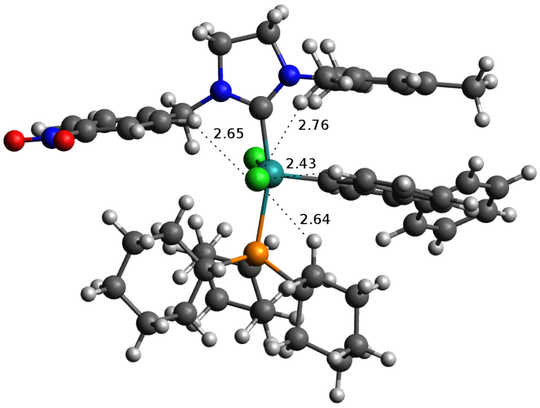

14b(3): E$_{rel}$ = 22.3 kJ / mol

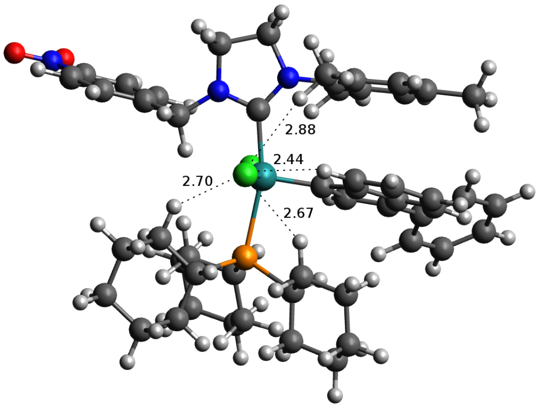

14b(4): E$_{rel}$ = 28.1 kJ / mol

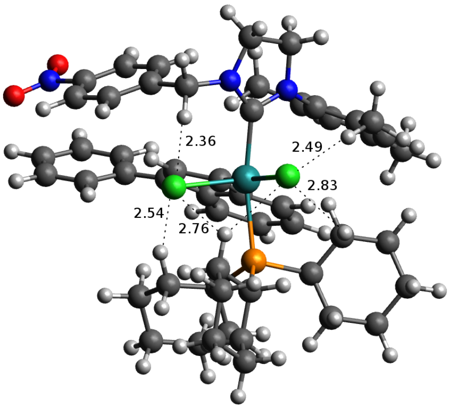

14b(5): E$_{rel}$ = 78.4 kJ / mol

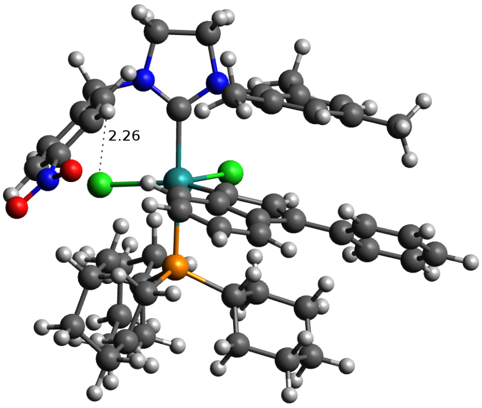

14b(6): E$_{rel}$ = 106.2 kJ / mol

Figure 2: Selected structures of optimized local minima of **14b** complex with the corresponding relative energy value.

Table 1: The structural parameters of C-H$\cdots$Cl H-bonds in **14a** complexes

Label	E_{rel} [kJ/mol]	H$\cdots$Cl [Å]	∠ CHCl [°]
**14a(1)**	**0.0**	2.90	170.90
		2.48	137.43
		2.59	138.49
		2.82	134.79
		2.88	145.54
		2.97	113.70
		2.70	118.16
		2.62	122.37
		2.67	117.28
		2.62	171.60
**14a(2)**	**6.0**	2.91	167.84
		2.49	139.25
		2.63	137.90
		2.79	138.90
		2.75	143.34
		3.04	111.32
		2.70	117.55
		2.66	117.57
		2.58	174.39
**14a(3)**	**40.6**	2.64	176.30
		2.74	120.11
		2.74	134.62
		2.60	128.99
		2.62	111.68
		2.76	116.91
		2.59	154.41
**14a(4)**	**58.3**	2.41	141.51
		2.55	116.21
		2.63	137.90
		2.98	151.95
		2.68	135.05
		2.72	113.27
		2.62	123.11
		2.76	112.02
		2.69	123.83
**14a(5)**	**69.9**	2.79	174.78
		2.63	121.68
		2.55	133.59
		2.39	152.54
		2.98	132.86
**14a(6)**	**82.1**	2.41	148.06
		2.81	110.24
		2.65	138.09
		2.48	163.45
		2.37	139.55
		2.56	140.21

Table 2: The structural parameters of C-H···Cl H-bonds in **14b** complexes

Label	E_{rel} [kJ/mol]	H···Cl [Å]	∠ CHCl [°]
		2.68	175.59
		2.36	142.76
		2.47	151.49
		2.67	127.67
**14b(1)**	**0.0**	2.59	144.34
		2.70	165.01
		2.53	119.83
		2.74	134.16
		2.89	115.67
		2.93	126.40
		2.61	165.47
		2.70	120.17
**14b(2)**	**17.7**	2.45	136.77
		2.57	121.09
		2.82	114.76
		2.95	110.14
		2.76	161.96
		2.43	139.78
		2.64	136.68
**14b(3)**	**22.3**	2.65	134.65
		2.72	121.88
		2.57	118.76
		2.55	117.05
		2.88	165.83
		2.44	141.55
		2.67	143.34
		2.70	138.71
**14b(4)**	**28.1**	2.64	145.70
		2.72	117.04
		2.58	123.43
		2.58	118.32
		2.51	176.58
		2.76	111.18
		2.54	152.55
**14b(5)**	**78.4**	2.36	128.56
		2.49	169.76
		2.83	119.86
		2.68	140.50
		2.26	139.23
		2.86	114.33
**14b(6)**	**106.2**	2.61	123.12
		2.89	123.91
		2.60	147.65

3. **The final xyz coordinates of the examined indenylidene-type metathesis catalysts**

14a(1) – global minimum

125
Coordinates from ORCA-job
C 0.66424970393847 2.26817380788507 3.23953003980830
N 1.89370261645935 2.73004739868184 2.97801344384945
C 2.27588711459624 3.83649388579400 3.88210870385391
C 0.92429928590116 4.22614095733525 4.48060721565197
N 0.15707030961560 2.98655661916720 4.26878671983538
C 2.88887543892701 2.08043895196817 2.18401525849845
H 2.74420453485200 4.64241252643709 3.31773869206353
H 2.97315476464641 3.47411225013635 4.64326441285647
H 0.45165207213573 5.04499382017445 3.92668678540974
H 0.99108148209031 4.48447580556700 5.53627932074401
C -1.22838564081009 2.85824576373628 4.68175124990559
Ru -0.58912922882720 1.12719407472610 2.07270289653130
Cl -0.61238835868456 -0.78490849380331 3.64685890707175
Cl -1.08054345810039 3.43750842899640 1.17547759007727
P -2.54987623607571 0.11836474537451 0.81460902590865
C -3.89931978645106 -0.50048884132307 2.00445081528608
C -5.26568760661799 -0.83346217298885 1.38622934742886
H -3.44327180735366 -1.42687059631867 2.37088300759162
C -6.19299913962952 -1.46349913482220 2.43720464560896
H -5.73222204481894 0.07970584628815 1.00658070313109
H -5.15879907695949 -1.50586957221510 0.53499330755446
C -6.35140459622293 -0.55500546148611 3.66147618716225
H -7.16961924896736 -1.67660913886696 1.99048665692688
H -5.77406072333043 -2.42678197517246 2.75352781770687
C -4.98813103861956 -0.19958814594582 4.26559404011151
H -6.86231959333733 0.36832044994430 3.35851779664672
H -6.98757110303472 -1.03541101767780 4.41178343985819
C -4.05463465551828 0.42489473496398 3.22099664271767
H -5.11128263908239 0.48568467376031 5.11052992180927
H -4.51583144589000 -1.10592502332559 4.66393904044932
H -4.45401042674757 1.39435350639371 2.90820804537917
H -3.07467475992011 0.59466626595796 3.66882689545903
C -2.03633733509218 -1.41641291112500 -0.20870557121418
C -3.23587088294614 1.43763852082562 -0.38948894599224
C -4.16440654540569 2.42949770279505 0.32766916037080
H -2.32006627691056 1.98907915971837 -0.61514852943934
C -4.45765105983657 3.63724221266991 -0.57009027797182
H -5.10801903894752 1.93931010802003 0.58686327804321
H -3.69533058652730 2.77164017288145 1.24859045002132
C -5.05404638428784 3.20463457135636 -1.91337924156107
H -5.13805495015307 4.32360914420019 -0.05468744282502
H -3.52240377026115 4.18370988354914 -0.73494279607597
C -4.14330001778554 2.19208608384508 -2.61495036448250
H -6.03718360515169 2.74609269501320 -1.74320177597886
H -5.22002978210446 4.07442970829559 -2.55678197457929
C -3.83895865343464 0.97932819697933 -1.72135284565464
H -4.59347917254435 1.85417310276075 -3.55404422283588

H -3.19755672760040 2.68359065991176 -2.87832452430346
H -4.76196478355552 0.41316862783386 -1.55066856293738
H -3.14565678203007 0.31907521121254 -2.24651798222516
C -3.10422874320089 -2.17355596462655 -1.01657506985338
H -1.30979661799962 -0.98116565638053 -0.90598374009114
C -2.42358125046091 -3.20625679726495 -1.92932718705729
H -3.76221786605951 -2.70355428080671 -0.32043009520446
H -3.73353985044478 -1.51442265872264 -1.60838286295970
C -1.58385066521504 -4.20527863705087 -1.12375943457394
H -3.18010764897947 -3.73430742971899 -2.51927934248037
H -1.77849039261689 -2.67816727115479 -2.64340687893861
C -0.60000219032136 -3.48767809632968 -0.19378056437037
H -2.25800596010002 -4.82878409669001 -0.52195102038909
H -1.05055289266579 -4.88238914661662 -1.79924866124668
C -1.30532556237810 -2.43958596454261 0.67234057974531
H -0.08456265348439 -4.20837118941026 0.44780314405912
H 0.17731529504858 -2.99320230935305 -0.78715284403172
H -2.04028291100787 -2.93822188004444 1.31729676656111
H -0.60222023190193 -1.95744912813795 1.33993237512498
C 0.55736320245185 0.59154863238496 0.70809332684899
C 0.57676432317291 1.21042567839189 -0.62343839462763
C 1.44744033014138 0.56308804525389 -1.44563893492150
C 2.04269855569022 -0.57158127776073 -0.69502568268772
C 1.52161835706308 -0.54307953562153 0.62256665773126
C 2.89181199834194 -1.58750465727740 -1.09925501327448
C 1.76909671690884 0.94913116519242 -2.81921814220352
H 0.03150533127144 2.10939259923159 -0.85943823177509
C 3.07741343658545 0.84552569217541 -3.31837150138716
C 3.37805847701573 1.26350543384340 -4.61058695745809
C 2.37884540060223 1.78150506965825 -5.43212038570045
C 1.07429333258027 1.88633862517142 -4.94962871554325
C 0.77263650101758 1.47649829208915 -3.65721032361812
H 3.86260959745333 0.46793400209913 -2.67681765355872
H 4.39591620370977 1.18671677283453 -4.97652222621140
H 2.61416479635156 2.10054919723082 -6.44078395907436
H 0.29079804640112 2.28383046137276 -5.58463419603831
H -0.24364365963500 1.54522985714419 -3.28705941230414
C 3.26874635656681 -2.56306014988396 -0.16255963507295
H 3.25373703844409 -1.64241593471817 -2.11805537203294
H 3.93858730708884 -3.36121676022738 -0.46343662005156
C 2.78351184435966 -2.51911281897020 1.13829259910596
C 1.89463602541836 -1.51059786529360 1.53826396753302
H 3.08237613383885 -3.27754679683039 1.85269928863736
H 1.48766530491384 -1.49771293277254 2.53903641797686
C 3.21274967859930 2.60142301358390 0.92720037435703
C 4.21688671911140 1.96362129958769 0.19641470469393
C 4.89824276724172 0.85603343379612 0.69662271458723
C 4.56290223493973 0.38099907433454 1.96687584440317
C 3.55769064440512 0.97240864448811 2.72675393352246
C 2.51219407310208 3.81365899628293 0.37512416707312
H 4.46337386607285 2.34230957444398 -0.78984202945270
C 5.95144379729660 0.15818580675113 -0.12418276924637

H 5.07714261741750 -0.48752386515004 2.36391337427836
C 3.16330057028815 0.41629398230253 4.06973455183493
H 2.61926298498966 3.85305141155523 -0.71057038061407
H 1.44995812406553 3.81518019819210 0.62130007236900
H 2.94929307365297 4.73346259324802 0.78237497077643
H 5.56520633650872 -0.79051818026287 -0.50935544285669
H 6.26365586879953 0.76752435147958 -0.97525533174398
H 6.83748165316798 -0.06878138909863 0.47459360219111
H 3.26931651611524 1.15322627564639 4.87106222053725
H 2.11777641681189 0.09750733931208 4.07897926848308
H 3.78241587865279 -0.44557356969231 4.32315843009501
C -1.42665066847629 3.27056487898106 6.12112669128031
C -2.47720446108592 4.12151815965203 6.46800392398280
C -2.66804889979177 4.54182431984137 7.78200064618923
C -1.78112030254724 4.14564155570580 8.77948811713467
C -0.70788061627965 3.32069450202341 8.45817302458724
C -0.56669846339313 2.88050239292110 7.14999578402464
H -3.14565501978575 4.46383011586069 5.68633461655834
H -3.49964520976903 5.19362484098843 8.02266528431865
H 0.01079150496433 3.01068926277824 9.20593432338683
H -1.88373006561586 3.45412308834551 4.03705626313542
H -1.49208905968514 1.80141349904749 4.57141987222783
N 0.56380977058979 1.96754240812979 6.86992277722523
H -1.91445879316759 4.48301711693250 9.79998921290743
O 1.69019270417880 2.39115482082962 7.11370589782477
O 0.29511458907129 0.86053196920971 6.44574882209013

14a(2)

125
Coordinates from ORCA-job
C -0.50855796004971 2.78470219139453 3.51576176199970
N 0.82644686401262 2.90544380693867 3.56386348996295
C 1.25273831241908 4.03518424779790 4.41806619772859
C -0.07150838769132 4.76245337281445 4.69211948042818
N -1.05055742978313 3.76826508434313 4.24828254776991
C 1.78656526285935 1.94333051920222 3.11871357713665
H 1.97810891555062 4.65460190711625 3.88919621519575
H 1.71338314060045 3.65673400587861 5.33351927924966
H -0.17250508594617 5.67769241418032 4.09836013073231
H -0.21332676027496 4.99576405328368 5.74732752633306
C -2.47917835166844 3.99010358894587 4.30368329301373
Ru -1.73785731732848 1.65657910973033 2.31458515788424
Cl -2.46823104121886 0.30207007347844 4.27185392885962
Cl -1.53045112244074 3.70158675302576 0.85852424892904
P -3.62499128548608 0.71307141893016 0.89897928610068
C -5.29076100465006 0.67142497429490 1.81871358202524
C -6.54930999272214 0.48863552973484 0.95766209901768
H -5.16558997188303 -0.21702882125839 2.44788342464275
C -7.78868062400555 0.31579875769634 1.84817768188726
H -6.69283913040568 1.36932783004611 0.32589679059513
H -6.44663946540735 -0.36540188483176 0.28897317604847

C -7.95626206270899 1.49901374498834 2.80815683418304
H -8.68080913053931 0.20006804215115 1.22394989617321
H -7.68704616954272 -0.60986521703157 2.42926855555125
C -6.69181926578855 1.71430158855759 3.64735662024912
H -8.16278451399018 2.40597941753156 2.22495401480846
H -8.82180861491179 1.33968854532580 3.45876101087655
C -5.44525057017049 1.86947955697128 2.76812382903610
H -6.80473062164856 2.59343722625991 4.29028219508172
H -6.54454902703103 0.85553867132216 4.31350113439007
H -5.51323279449220 2.79718470633369 2.19017695080992
H -4.56417640160040 1.93358200063011 3.40680365370882
C -3.29703662119152 -1.10429956649609 0.39953891929495
C -3.73844727615182 1.75610602556332 -0.70032876478441
C -4.52922728714138 3.05509122103020 -0.47942835057794
H -2.69280577722491 2.05376179336239 -0.80538547409233
C -4.32839315218794 4.00968739066011 -1.66244768720882
H -5.59527733062228 2.83285706127986 -0.37576297573322
H -4.19280085697494 3.53945935242519 0.43647310213244
C -4.71912404974511 3.34732789461265 -2.98774296840851
H -4.91248753137996 4.92206320028472 -1.50557590519378
H -3.27469857413298 4.30809017688625 -1.69010206261025
C -3.96168651683543 2.03134553456307 -3.19014085882263
H -5.79848533114419 3.14388369553122 -2.98758239932063
H -4.52774194047321 4.02539552542921 -3.82594153670282
C -4.15246810023394 1.07015704511214 -2.00575425904103
H -4.28274841251448 1.54335533560201 -4.11676709921762
H -2.89126577232533 2.24702240233365 -3.30112881064322
H -5.20067109900170 0.75443050314489 -1.96033440791825
H -3.54997162320674 0.17590174987695 -2.17589517419391
C -4.35166369008799 -1.85015961812428 -0.43559542193582
H -2.37933394813654 -1.01109334891092 -0.19289109392180
C -3.78048524618847 -3.19870130419606 -0.90473200208742
H -5.22792187252294 -2.04336259976795 0.19243824844078
H -4.69542427422286 -1.27674608887023 -1.29276567491859
C -3.35240958671602 -4.07121277489669 0.28121218672520
H -4.52364852160040 -3.72345240753637 -1.51472876270649
H -2.91493485891371 -3.01113655099172 -1.55271518541291
C -2.40413388217621 -3.31450174801356 1.21847778568371
H -4.24788129304265 -4.37015931310752 0.84160556469525
H -2.88475362462362 -4.99445232215463 -0.07589359262359
C -2.98663528873086 -1.95971987126336 1.63712196535039
H -2.18783794997776 -3.91266313716225 2.10849795022734
H -1.44341375804161 -3.14525646673833 0.71882517176684
H -3.91417879170463 -2.11944325548504 2.20079441501440
H -2.31278244135413 -1.44775429936409 2.31213539273632
C -0.50990299818535 0.59439493648982 1.40699757619680
C -0.08528225303208 0.85108436998036 0.02441770915478
C 0.76487848302214 -0.12035271313906 -0.40859509876552
C 0.91894280911160 -1.12321587903508 0.67643294005653
C 0.16707647220596 -0.67776206314652 1.79150451540318
C 1.57338324381474 -2.34294480913133 0.70663769216163
C 1.44671706375714 -0.13879132742008 -1.70248719019577

H -0.35424930813273 1.74516180678177 -0.51326207091643
C 2.76822521490196 -0.59772678577100 -1.82335934577557
C 3.42393529793412 -0.55698877274883 -3.04930268354814
C 2.77063374555244 -0.06948030497932 -4.17928301369788
C 1.45646059819649 0.38570051260075 -4.07385745703062
C 0.80191909018669 0.35344130494031 -2.84922461337455
H 3.28961442336208 -0.95354636323430 -0.94498689332161
H 4.44837356845454 -0.90373580803968 -3.12189205517395
H 3.28091583780943 -0.04396202118860 -5.13549954702214
H 0.94083082437479 0.76243960093663 -4.94992087222985
H -0.22312593088216 0.69572493251274 -2.77039904928200
C 1.51851385693140 -3.10861295824801 1.88215497549364
H 2.10722780781467 -2.71389953618777 -0.15957901948158
H 2.02878585059130 -4.06441516787369 1.92050195136483
C 0.80807128471924 -2.65745983353393 2.98775326071279
C 0.11646867110116 -1.43854582916611 2.94600285361800
H 0.77186366385647 -3.25819739770635 3.88934247765487
H -0.46455263447709 -1.10271423845744 3.79315403656546
C 2.42760914408517 2.13040853301781 1.88908816771106
C 3.38288907312078 1.18936052541631 1.49944124957877
C 3.71805644188050 0.10526171017078 2.30789286847010
C 3.07261826171182 -0.03602666620900 3.53820299985494
C 2.09911365291270 0.86466410000763 3.96090577994235
C 2.10808519443008 3.30921739154090 1.01043710941464
H 3.86934971764453 1.30825113080162 0.53686452951477
C 4.73259335124049 -0.91490121392471 1.85988658267819
H 3.30975153756922 -0.88553322417885 4.17012608701397
C 1.37198456234568 0.66616603974801 5.26392268523911
H 2.47903032395711 3.14128096057543 -0.00233933596809
H 1.03588229827290 3.50020260319193 0.96222813802618
H 2.58879984925222 4.21833789277824 1.38985929140569
H 4.24053892693367 -1.86092305818525 1.61316086662381
H 5.27667946643716 -0.57426816476969 0.97624532617614
H 5.46183074550056 -1.12295446503382 2.64777808474939
H 1.59340040387064 1.46242970991547 5.98132100693486
H 0.28820882954156 0.66320153554197 5.12631877895972
H 1.66232878400584 -0.28114479761128 5.72118083481774
C -2.97976408961421 4.61192344035248 5.58949140588116
C -3.63683275001054 5.84351382757370 5.51943812256301
C -4.21892182724603 6.43301911179460 6.63815970870546
C -4.17937767980081 5.78116016126260 7.86651497555092
C -3.54949060421661 4.54573973283621 7.96678865719977
C -2.94404232112946 3.99418181560875 6.84514857851467
H -3.69544788236204 6.33920639173951 4.55715999729109
H -4.70939086875300 7.39440648925685 6.54603084466171
H -3.51973538420859 4.00320363128705 8.90279231485974
H -2.79781498302373 4.61689421468146 3.46407742245822
H -2.95264411891313 3.01028381315556 4.19176278235583
N -2.23942486757558 2.70926766702568 7.07146530513890
H -4.63912848696981 6.22369572546046 8.74163144302247
O -2.88131100181789 1.80959342830580 7.58362503602281
O -1.05099049358807 2.66898590122939 6.78708347166356

14a(3)
125
Coordinates from ORCA-job
C 0.60331843674079 2.40785765942700 3.11834567621518
N 1.88911852903463 2.03365257152189 3.24671052618946
C 2.62034073251131 2.81993344006771 4.26210618927799
C 1.46597589682767 3.46901973512061 5.01687333329518
N 0.36607702695292 3.36506194062216 4.03836599106067
C 2.56349586871313 1.02517143792876 2.49360918365951
H 3.25626326903031 3.55942743714304 3.76895585709939
H 3.23396953201541 2.16600746379811 4.88208311999444
H 1.18885604411376 2.90401609416623 5.91478264854781
H 1.65540026938415 4.50756847788946 5.27507073700092
C -0.92226102695073 3.91725924030326 4.39298097000330
Ru -0.71999578117405 1.40859422757937 1.83520786062396
Cl -1.71580637232818 0.62757774204437 4.10888124821345
Cl -2.06619073653955 3.39939667859791 1.46299332574613
P -2.40997138857264 -0.07527206744445 0.72762447086806
C -4.14214141910170 -0.15042813602185 1.52027933850251
C -5.24613121322720 -0.76608973565570 0.64754739565368
H -3.96357367127028 -0.79808028719710 2.38582932080214
C -6.54232531219667 -0.92414521960446 1.45597977108704
H -5.43699530425787 -0.10483475350018 -0.20439083398709
H -4.94946463902117 -1.73071564065210 0.23814916728733
C -7.00661676711353 0.41454990931560 2.04038448592274
H -7.32342494076894 -1.35875601922387 0.82282828305947
H -6.36637361480971 -1.63441911339536 2.27397998626151
C -5.89157223010984 1.07292229028020 2.85973093357995
H -7.29357691453569 1.08340916534848 1.21840579358154
H -7.90201707498934 0.26956868771750 2.65407776786376
C -4.59420068604261 1.21549132432529 2.05565160518055
H -6.21183656537361 2.05750593934308 3.21527101419544
H -5.68777942040703 0.46863694047442 3.75268277430597
H -4.74899562846468 1.90506161902131 1.21954225992811
H -3.81588103054500 1.64564878053939 2.67965972529628
C -1.69343863411661 -1.84614968348751 0.84043385396714
C -2.50854828930770 0.24109488044129 -1.14709303967018
C -3.15275293220278 1.59544016448814 -1.46637282798262
H -1.44483299266046 0.32285898648882 -1.38525389100719
C -2.93015358720358 1.94835027560226 -2.94222494844750
H -4.22862409523383 1.54645433663494 -1.26060622030152
H -2.74209477529485 2.37173655494858 -0.82048858511478
C -3.45308039384635 0.84736327508526 -3.87175746950044
H -3.41033814904384 2.90420686642994 -3.17549191410912
H -1.85557775218568 2.08658537214532 -3.11171458086926
C -2.84238009813130 -0.51428213517919 -3.51884434298196
H -4.54541970024017 0.78607998522577 -3.77855809526264
H -3.24034121032511 1.09673982836623 -4.91675891609064
C -3.07032294547750 -0.87180595071756 -2.04115625015808
H -3.26054155752557 -1.29799153464688 -4.15909799169442
H -1.76269334267842 -0.48709539724985 -3.71353547647996

H -4.14075182175531 -1.00372320008102 -1.86140537239479
H -2.58431666721956 -1.82615917373512 -1.82409542616402
C -2.68473927515109 -3.01540752900758 0.80014088557507
H -1.06271563331254 -1.90632628167618 -0.05224863762090
C -1.93842067119778 -4.35802332502259 0.80821331697581
H -3.34893378797089 -2.96442624288138 1.67031033530262
H -3.31637622453511 -2.96248657625809 -0.08689541871549
C -1.00173537790327 -4.48302885375395 2.01315248738705
H -2.65919594695905 -5.18185687265695 0.79849483651747
H -1.35181737964225 -4.44204411986002 -0.11624059119601
C -0.03255711715020 -3.29827899128823 2.08688911390576
H -1.60004891573181 -4.51172710471331 2.93248560259896
H -0.44826116319968 -5.42655644602621 1.96826738787750
C -0.78910724470515 -1.96724642774163 2.07507281699644
H 0.58239678971542 -3.36797849824911 2.98868511985248
H 0.65566532324375 -3.33519388759466 1.23120987026274
H -1.38133117626727 -1.85337022592008 2.98590148390579
H -0.07273851577724 -1.13737641589641 2.10576107190771
C 0.20330480690610 1.74731299983757 0.21161319121962
C 0.24193302594794 2.98529451064311 -0.56391419626349
C 0.78834367489528 2.78584209026190 -1.79691954866305
C 1.19610770625141 1.36114542720490 -1.90118419282008
C 0.89413311014370 0.74327184786491 -0.66178641655723
C 1.70951997433139 0.62132056160477 -2.95258373217620
C 0.92996945009995 3.80048639898921 -2.84015488245480
H -0.12096208758485 3.92432815379382 -0.18482016341872
C 2.04216255623688 3.82718768316266 -3.69657342630051
C 2.17255469807651 4.82168955597298 -4.66015430753052
C 1.19014076413868 5.80040223758109 -4.79548054198342
C 0.07922466697912 5.78442093598094 -3.95226885099723
C -0.04965048520972 4.79799093570365 -2.98313841017759
H 2.82325922117464 3.08647299511242 -3.57992412199022
H 3.04554960227246 4.83589496374636 -5.30341573175848
H 1.28919803010825 6.57081931020587 -5.55203252059264
H -0.69116143056612 6.54082514905628 -4.05468715265343
H -0.91985732220749 4.77698272548462 -2.33764949217971
C 1.97561023740409 -0.74489193365558 -2.75328220255348
H 1.88841639186103 1.07265433037282 -3.92068094401995
H 2.38043221790780 -1.33393351233431 -3.56852540469109
C 1.72556506516420 -1.34145379915189 -1.52525249068007
C 1.17721057442169 -0.59462178744082 -0.47165849667210
H 1.95054050289514 -2.39080317638372 -1.37588743134880
H 1.00670530697236 -1.06292699328717 0.48403876878276
C 3.32473337167037 1.38720435640415 1.37589665683374
C 4.04093809706793 0.38548214970178 0.71700087931281
C 4.01349831749434 -0.93879106644552 1.14284594201919
C 3.25260845759492 -1.26353224458469 2.27012826839563
C 2.53178878657542 -0.29813353415073 2.96990001039006
C 3.39154155106348 2.81131982453159 0.89143283968771
H 4.61185786788940 0.64794032237508 -0.16766704228537
C 4.77815042026801 -2.00838528836110 0.40602164179733
H 3.23055534602372 -2.29017239175811 2.62206908665558

C 1.79113083663490 -0.65091572522827 4.23478372773158
H 3.48205496043827 2.83970995647952 -0.19597185289605
H 2.51219943457080 3.39051595214741 1.16632440354285
H 4.26785675230008 3.31960410504098 1.30985461119239
H 4.10601234306735 -2.79026325815079 0.03965574370385
H 5.30806000001408 -1.59525727532713 -0.45373908875126
H 5.51211841237880 -2.49066558854174 1.05915143013855
H 2.26958326565746 -0.18416604070571 5.10283814119737
H 0.75237138032912 -0.31408719805747 4.22874301650486
H 1.80165614516677 -1.73031950063243 4.39403462434358
C -1.25499230474386 5.30036504241667 3.86099063927761
C -2.51416120919273 5.80255271330783 4.21169126844008
C -3.00394481806540 6.99399237657277 3.69723822446276
C -2.24526926541607 7.72490665901115 2.78437211842306
C -0.99661390570523 7.25272101652043 2.41053282847655
C -0.51452699183441 6.07330836174943 2.97098611503322
H -3.13241637041859 5.21155687625343 4.87909031955806
H -3.98625558658638 7.34312871893103 3.99200490634768
H -0.38356844494215 7.78935412466729 1.69729322292119
H -1.69493086853075 3.23130844358155 4.05236052209252
H -0.98141920703667 3.93907164750260 5.48631700127575
N 0.85277302114208 5.70590726127079 2.54064264917380
H -2.62295334555350 8.64832780908684 2.36355074128449
O 1.01990771784394 5.41347317492750 1.36750437981686
O 1.74957696768282 5.79866312704428 3.37015660879508

14a(4)

125
Coordinates from ORCA-job
C 0.14660223313824 1.30136649533695 2.10775417520599
N 0.55182826914502 0.83380075033204 3.30962745845117
C 1.88481815559457 1.33169831238398 3.70931849914810
C 2.17528539375556 2.38418553997494 2.64277436544957
N 1.08782322814819 2.16535479399219 1.67860481108424
C 0.01199842306769 -0.24610385849945 4.07891387160428
H 2.60236292813519 0.50475121481387 3.69914858935832
H 1.85156015870838 1.74084534654508 4.71914131480920
H 3.14626108910336 2.24847327778244 2.16000344224925
H 2.12090026624564 3.40631382818860 3.03015087147599
C 1.15239250923024 2.86890281666848 0.41573386594979
Ru -1.64378206660063 0.80313546086993 1.06089447739607
Cl -2.96372614434771 0.73516557937454 3.25312210264435
Cl -2.13562327736185 3.17585860816325 0.83517965541111
P -3.83258071181777 0.02835579774620 0.13194624860294
C -5.42986263191543 0.53931514523146 1.02564689859182
C -6.74994286803441 0.09966372556417 0.37571234313607
H -5.31479296886505 0.03261356076570 1.98822595671401
C -7.92824984206109 0.43323840398507 1.30462713504211
H -6.88574291877258 0.63100543758556 -0.57242516092242
H -6.75498892313310 -0.96552617698869 0.14665227095947
C -7.96558723717490 1.92492679926383 1.65456820119039

H -8.86870598581442 0.12437582152500 0.83501553042455
H -7.82845169386691 -0.15200309679354 2.22725523852979
C -6.62591475486588 2.39123177775891 2.23562458489595
H -8.18234579445661 2.49960532364902 0.74412157742120
H -8.78217259332713 2.12789304190903 2.35543363111434
C -5.45233426885625 2.04940719265978 1.31070988168256
H -6.64531655926743 3.46941677582322 2.42070936223651
H -6.45577049323142 1.90938464017354 3.20614625440521
H -5.55612642774391 2.60249817754633 0.37136831635375
H -4.51520529560465 2.35792033108592 1.76417596711812
C -3.56675975945301 -1.84751408356985 0.34979056850970
C -4.02967580852711 0.24074942313613 -1.74328588644961
C -4.37230472806006 1.69329920448966 -2.10346764517465
H -3.01091024053586 0.04811957157215 -2.09054280052252
C -4.26992656968666 1.90473708632413 -3.61828894725900
H -5.39388741980072 1.91524293579387 -1.77736113848952
H -3.71507450611406 2.38082729809778 -1.56607824370272
C -5.15518415502543 0.91451096197394 -4.38296169683234
H -4.54288049834391 2.93441058892599 -3.87169451026416
H -3.22774658222289 1.76895202246659 -3.92598941473372
C -4.83015390371717 -0.53344078689660 -3.99476853319644
H -6.20948706507882 1.11984521019653 -4.15413593729340
H -5.03493827953838 1.05244738305536 -5.46233969121581
C -4.94320032262427 -0.74875436208685 -2.47769692269224
H -5.49158967244107 -1.22892267635040 -4.52057066894908
H -3.80584255105095 -0.76874213955510 -4.31145421607229
H -5.98322153727611 -0.61033767513650 -2.17096140474067
H -4.67080081703047 -1.77929285744071 -2.23531631436113
C -4.80771742204601 -2.72901045339035 0.52453325711642
H -3.06712221455852 -2.12933276075062 -0.58526435435375
C -4.41773309747775 -4.21080540201115 0.62343217359243
H -5.33838396519765 -2.43111725329382 1.43563647622590
H -5.49929153797424 -2.58712513411876 -0.30725113991768
C -3.40536722511586 -4.45295710079104 1.74694178600519
H -5.31240467343729 -4.82222233858734 0.77513795048011
H -3.97713843443267 -4.52680557467548 -0.33143153025641
C -2.16903254555041 -3.56162315220806 1.57630326811031
H -3.87613609733750 -4.23031723963812 2.71325615663287
H -3.11018889448440 -5.50659122237391 1.77465411079963
C -2.57916036841029 -2.08966242545428 1.50127305239402
H -1.47784942668480 -3.71051016219427 2.40700531655229
H -1.64723520624393 -3.85179936811335 0.65529366423941
H -3.00825545357434 -1.76276530830527 2.44983586585336
H -1.67153473094459 -1.45965657519251 1.38403182120019
C -0.94871285065931 0.69112160294631 -0.72033949531700
C -0.96466490686073 1.73673572480102 -1.74009381395033
C -0.46827231318094 1.30349043502308 -2.93699300669791
C -0.16115953571004 -0.14506229624480 -2.80050938751712
C -0.42379783372110 -0.50654250302132 -1.45270039629529
C 0.25157207232248 -1.10182841905163 -3.71418015328355
C -0.21261068538210 2.15846825645873 -4.09531113382376
H -1.27384193915094 2.74482191934228 -1.52375971688950

C 0.76667972489779 1.83958007331294 -5.05159240699405
C 1.06595076911747 2.71536055620746 -6.08902224374479
C 0.39358994488236 3.92916004362655 -6.19930553411969
C -0.58482692715517 4.26004758592408 -5.26200534944796
C -0.88547136714062 3.38789490931542 -4.22673737523677
H 1.33623441386308 0.92790585763975 -4.95047783017192
H 1.83937255384558 2.45627105404087 -6.80354772558237
H 0.63539143640107 4.61720407155447 -7.00098728447513
H -1.10744562702149 5.20648304756639 -5.33244301936475
H -1.64369911045260 3.65868705686298 -3.50392347972063
C 0.46240608159271 -2.41967059556311 -3.27595942197168
H 0.39744929186487 -0.85936760894713 -4.75812427935321
H 0.78877546832006 -3.17117457200757 -3.98592932317367
C 0.25268234244555 -2.76580878844982 -1.94780829542959
C -0.20330638981652 -1.80577485380098 -1.03211872624012
H 0.42548907714142 -3.78352665684652 -1.61701167224095
H -0.37860340882046 -2.09112192741979 -0.00733887220816
C 0.27984691474810 -1.56284175780220 3.68427730252280
C -0.18953423013624 -2.60298775462091 4.48512677458430
C -0.89906894023686 -2.35677839973737 5.66147507368272
C -1.10645385680253 -1.03329147929932 6.04501746610866
C -0.65280357604904 0.03809827698916 5.27672780416867
C 1.09956390174222 -1.85863966105874 2.45395892188603
H 0.01547173465965 -3.62747505627557 4.19004743816833
C -1.43567742377392 -3.50085931307224 6.48459007088714
H -1.65301870627877 -0.82376980343974 6.95833915473551
C -0.88066338916652 1.45746593145222 5.72135520507108
H 0.98752911901637 -2.90154009275274 2.15321276725950
H 0.81356531449961 -1.22970479669467 1.61314681650460
H 2.16570380839845 -1.68297080012280 2.63855158933243
H -1.79840038438283 -3.15667140558336 7.45455657783467
H -2.27083274650370 -3.98992248759807 5.97225981268505
H -0.66912755926710 -4.26161375992663 6.65871290389354
H 0.04098109905513 1.90371292348733 6.11357490851503
H -1.24002176788629 2.06449751329436 4.89075027972889
H -1.62952525938215 1.49258514756537 6.51360348570265
C 1.98844726499853 2.13476841898338 -0.62318897400134
C 2.38990308396948 0.81711141615443 -0.38536381189031
C 3.13279268124610 0.08859517720607 -1.30634133638086
C 3.53166804265086 0.67309809137372 -2.50373491826763
C 3.16108780094488 1.98174004762396 -2.77140923956022
C 2.39030708427898 2.68774241329224 -1.85124890899413
H 2.08445783791828 0.34556306550678 0.53603343643268
H 3.38555021619448 -0.94228655964972 -1.09008256514324
H 3.43459069668284 2.47510302894125 -3.69194953255704
H 1.57107466086091 3.85969831723058 0.60306833034130
H 0.14534245201798 3.04936277670167 0.06063092016821
N 1.97266380778953 4.04117278596263 -2.26853184213750
H 4.11052301866806 0.11379835093363 -3.22847969254070
O 1.06707218407799 4.58129800846007 -1.64135237693812
O 2.54085134666918 4.54731198489046 -3.22648910752135

14a(5)

125

Coordinates from ORCA-job

C -2.03402074423166 2.00008673958187 4.09714096255517
N -0.73451291819533 1.97370799483405 3.71226385343418
C 0.12776618663711 2.78664469316778 4.59587464944571
C -0.90818049219480 3.66258932311501 5.28922173968478
N -2.13021243216827 2.84794483698539 5.13747618577815
C -0.16322263010800 0.96145956212726 2.88434677586965
H 0.85520343971112 3.35497467623737 4.01782417134623
H 0.65069663602916 2.13824639070713 5.30448171943136
H -1.04875069374118 4.62471187232735 4.78129971162842
H -0.67155646131245 3.84445855303385 6.33513310313119
C -3.35126905258112 3.18675729753781 5.85539900901419
Ru -3.56808724757214 1.02695324318759 3.01173158656767
Cl -4.88932586060710 3.07737864069771 3.03752283902728
Cl -4.50998101076333 0.06250596060499 5.20856431039371
P -5.47463024526610 -0.21859670174718 2.01289738661487
C -7.08542580490090 0.10710392261928 2.99481540802861
C -7.92958171474328 -1.11253534451541 3.37269153238428
H -6.66408645310564 0.50896005568203 3.91825939107977
C -9.09355385023394 -0.69726280377140 4.28463430930536
H -8.31781608583187 -1.61005576838883 2.47615279728217
H -7.31036679772111 -1.83367853586932 3.90488383055155
C -9.94820491413636 0.40783249017979 3.65816975123951
H -9.70798306853624 -1.57282507928446 4.51886238785734
H -8.67797080626478 -0.33867828951627 5.23344816203172
C -9.08414106696893 1.61345814871802 3.27625901448410
H -10.44189435764649 0.01768197600530 2.75775196258574
H -10.74414966162718 0.70968925147401 4.34684547428505
C -7.93380076387464 1.20846698186879 2.34557326069859
H -9.69309717169918 2.38413651488620 2.79205414048462
H -8.66346088591912 2.06517961482275 4.18203998238051
H -7.31126928780233 2.07762767113312 2.13486853051629
H -8.35354119286148 0.85449823847069 1.39772280230692
C -4.85282657126605 -2.04301821338913 1.98396584654935
C -5.73145130506115 0.04382242112516 0.14313878095037
C -5.86818171266318 1.50813398353212 -0.28630770659008
H -4.75936136250116 -0.29896447918486 -0.22487766657674
C -5.71448174882423 1.61673365795559 -1.80822863557998
H -6.85084220389425 1.89011218684458 -0.00214949712132
H -5.13384228817413 2.12779386635733 0.22467733967653
C -6.75696984569081 0.74995639342771 -2.52572713750654
H -5.80930222604253 2.66207561952686 -2.11958988683457
H -4.70577832499677 1.29473638235617 -2.09335607040904
C -6.69731389152874 -0.70834411384043 -2.05401148476186
H -7.75801257373538 1.15226020298105 -2.31966097377414
H -6.61717927481234 0.79905451387620 -3.61050870596208
C -6.80319863656976 -0.82589733953228 -0.52420497742044
H -7.49026125284030 -1.29505541610460 -2.52904068724522
H -5.74436767994420 -1.14962295312621 -2.37372512776409
H -7.79785009393260 -0.50573600550971 -0.19674896886871

H -6.69453421749719 -1.87373573726956 -0.23431513283397
C -5.82239049575972 -3.22280416138166 2.13001818421038
H -4.46143480461238 -2.08369591086065 0.96174927992085
C -5.09378280936490 -4.53614914213456 1.80536031706773
H -6.19004691412594 -3.27311915585430 3.15707977619397
H -6.69575162115286 -3.10319986359149 1.48635816597534
C -3.87514781726047 -4.73744057629131 2.71446598860052
H -5.78419611071828 -5.37958211281804 1.90746674902391
H -4.76932173864118 -4.51935346685429 0.75636598981694
C -2.92400581291917 -3.53554581009907 2.65256408559638
H -4.21808737452497 -4.86659950924672 3.74805618907099
H -3.34402559382048 -5.65524015075954 2.44193630858178
C -3.67020125040234 -2.22910213872132 2.94201367599869
H -2.10399537372783 -3.65885616066467 3.36653863833007
H -2.46717850699217 -3.48828549087748 1.65532808933818
H -4.02157021233787 -2.20327066898202 3.97421227453677
H -2.96893903270108 -1.38437561479136 2.86111494606164
C -2.81213007253482 1.51581849561098 1.33734410619871
C -2.71725832373627 2.86380371921862 0.77713337909790
C -2.27102579453454 2.84601530249668 -0.51095604206108
C -2.00493202631437 1.43547605614284 -0.88752324948546
C -2.29736226292029 0.63132103817326 0.24321943492469
C -1.60797646435976 0.86102463819017 -2.08281544916379
C -2.09168348006867 4.01563525353798 -1.37016095237532
H -2.99161623493495 3.74443066161255 1.33418546480530
C -1.01995210907116 4.10943378158626 -2.27230372756063
C -0.84545554830430 5.24853627406136 -3.05139425813358
C -1.74345000568845 6.30937637641472 -2.95392186574310
C -2.81463063374091 6.22751906304345 -2.06451196112480
C -2.98669245147332 5.09483934716686 -1.27908030153402
H -0.30529782631386 3.29848063481745 -2.33749055975402
H -0.00436588531268 5.31023501951304 -3.73316890058757
H -1.61037765322814 7.19387677971845 -3.56653252594318
H -3.52017493248913 7.04745985667273 -1.98754822315007
H -3.82766858666469 5.02263657952992 -0.59929081025315
C -1.45966830025987 -0.53537225115648 -2.14738514707718
H -1.43329659556153 1.46480054068454 -2.96464269542257
H -1.14855351406202 -0.99702017088733 -3.07782256777512
C -1.71210672510193 -1.32370390398873 -1.03293436816075
C -2.13572574051841 -0.73902214831725 0.17049476476049
H -1.58575439982231 -2.39885283757155 -1.08873823399436
H -2.32050041306255 -1.36269734352462 1.02854358082753
C 0.48707472653200 1.33229935965585 1.69698871158166
C 1.03021068042139 0.32858287104182 0.89758210945443
C 0.94352622085202 -1.01693486976810 1.24925391108220
C 0.32794811426590 -1.34840432122753 2.45705412623260
C -0.21085289165419 -0.37926641422956 3.30535265544486
C 0.62037054158708 2.77537259553357 1.28638221138663
H 1.50410242068998 0.60496870657089 -0.03907272786607
C 1.51148928834616 -2.08093936597115 0.34581267163106
H 0.27967358142266 -2.38953798666885 2.75993266271525
C -0.76174207076409 -0.76985620117705 4.65528664931903

H 0.61801560526120 2.86887658695073 0.19925984205028
H -0.19577731430620 3.38109506933202 1.67632603072614
H 1.56543059514568 3.19557508856515 1.64990491802927
H 1.29308468095849 -3.08137864388732 0.72501706551796
H 1.09008272447413 -1.99836321635374 -0.65964560854330
H 2.59848894003095 -1.98521261694017 0.25697030642021
H -0.28083301889772 -0.20546687998189 5.45757661964751
H -1.83299694146625 -0.58837320893509 4.76560208739953
H -0.58414977169090 -1.83032850423362 4.84007847491938
C -3.09756510467705 3.49975382198110 7.31283125953471
C -3.71668151514973 4.61753433705804 7.88111100360575
C -3.52489998201371 4.96643647524607 9.21435587327760
C -2.66742496708372 4.21866429342097 10.01698157173081
C -2.01539965561785 3.11628927965180 9.47725457178226
C -2.26148058679688 2.76433461920500 8.15654390974795
H -4.35676439518404 5.22537876845384 7.25139830037190
H -4.03128502288265 5.83397129169478 9.62073509254681
H -1.32913440231040 2.52245348776227 10.06774438180411
H -3.83715863293171 4.04631542868481 5.38941649279769
H -4.02763793470706 2.33518877659551 5.76218499993535
N -1.56528911284895 1.55305576181468 7.66793353875551
H -2.49844437597639 4.49323537067322 11.05095278565137
O -0.33567604070597 1.60311359980526 7.61584545918333
O -2.24591031296913 0.58892871392116 7.38732044313498

14a(6)
125
Coordinates from ORCA-job
C 0.62743031027453 3.67355334681425 2.08877505925612
N 1.77925123714653 3.13149151025180 2.54448607468933
C 2.60693279680982 4.07975500886338 3.30944978328147
C 1.64410682217835 5.26159292649047 3.47550055144634
N 0.55471409040389 4.92863956261962 2.53908881025648
C 2.30845046175664 1.90259377098690 2.03233664883855
H 3.50283762588270 4.34288838826616 2.73856746156035
H 2.91485918972805 3.64584876557804 4.26067711602718
H 1.24248804911144 5.33353403868879 4.49009005453627
H 2.08932123175882 6.22281964705041 3.20899119295084
C -0.46091482884475 5.93153424620279 2.26942513284015
Ru -0.61428037292653 2.35297932142944 1.03295827624332
Cl -1.39511491736932 1.66252471170421 3.39863195300345
Cl -2.54971861719888 3.81597944503692 0.90754675195967
P -2.06207468173276 0.43866590191539 0.25319305690089
C -3.79276344964927 0.47778218728913 1.08325077920064
C -4.41058461877472 -0.86658393655828 1.48975344852792
H -3.54983762879411 1.02752937292257 1.99449259921603
C -5.50604153292804 -0.61380345983162 2.55541764025668
H -4.84153897556179 -1.35217237975540 0.60672387753915
H -3.67979707106145 -1.56012673062559 1.90074119831412
C -6.18961733258719 0.75816447104101 2.38151967980566
H -6.25207464353411 -1.41385854640894 2.49749333414191

H -5.05738334871850 -0.67134738644138 3.55160573205065
C -6.25495198754449 1.13527215359811 0.90029615908075
H -7.19067168331884 0.73807068440780 2.82217102172661
H -5.62759234535684 1.52574861823085 2.92262144521403
C -4.83847518980603 1.28225822231840 0.28598900801271
H -6.81424679313506 0.35725423580615 0.36650926890690
H -6.81237076147043 2.06546516860762 0.76143182728274
H -4.87756007480577 0.93062547535886 -0.74572186292891
H -4.53479375839263 2.32714378873805 0.26329621126665
C -1.40780850054086 -1.30747679051427 0.70361948896081
C -2.24932921004982 0.30536624185780 -1.63648567810616
C -2.63725452124487 1.62288583386008 -2.32131817720516
H -1.21757875003257 0.10730534898601 -1.93845646656995
C -2.45834287363907 1.48979054919321 -3.83809887712357
H -3.67571915171327 1.87871746878499 -2.10182178260314
H -2.03922254873527 2.44639841233399 -1.93342911965106
C -3.29351051481242 0.32654790566153 -4.38850816359360
H -2.74074217795601 2.42632899152409 -4.32967482195634
H -1.39762333461962 1.32224109861752 -4.06272988717853
C -2.96647807067646 -0.98953154854945 -3.67120347138372
H -4.35843762823582 0.55527353337180 -4.24887077345051
H -3.13505814518802 0.21597137840902 -5.46629372685331
C -3.10461819203865 -0.86250953572869 -2.14534753160086
H -3.61193950614468 -1.79397687893828 -4.03853338676639
H -1.93489547043173 -1.27944614853173 -3.90843305526633
H -4.15532683233364 -0.69987912077770 -1.88457172046338
H -2.80727236344608 -1.80185525855046 -1.67076680735777
C -1.06481743207251 -1.44245531256977 2.19335017400243
H -2.24889136155859 -1.96897415445637 0.47709657714748
C -0.76081623463087 -2.90476342287880 2.54176024973244
H -0.19490099430779 -0.81794015374429 2.41221427867676
H -1.85723229479007 -1.05078734311568 2.82862784296770
C 0.37245337917083 -3.46909235036139 1.67752594068681
H -0.50472962210770 -2.98621855878253 3.60314706409141
H -1.66628655661863 -3.50722657526092 2.39147777188716
C 0.09476278634661 -3.26130467379940 0.18499414721828
H 0.52771704087277 -4.53142093479214 1.89089820614105
H 1.30467787986536 -2.95460290549841 1.93623565961014
C -0.21320854251391 -1.78965925930241 -0.12825263172713
H -0.76045930282844 -3.87797202974913 -0.11967973067520
H 0.95005070421038 -3.59755125741354 -0.41160271243829
H 0.66466659290556 -1.18888726794749 0.12834616791392
H -0.38881141869795 -1.67211411988027 -1.19934439239333
C -0.03123055716134 2.95865240043429 -0.67873754029608
C -0.23227828204321 4.30077165803139 -1.22978814806400
C 0.26355722073992 4.40764720388242 -2.49733439729464
C 0.87559728535578 3.10450644962835 -2.86220677368102
C 0.69524360360420 2.23344275822987 -1.75986498405684
C 1.56571013120942 2.68415754399745 -3.98600457591445
C 0.17297291490659 5.58646745920408 -3.35777425788684
H -0.78939499545457 5.06927177998918 -0.72056397672834
C -0.05064892001417 5.46551963405775 -4.73910657520629

C -0.18582716082476 6.59614499564663 -5.53704763359906
C -0.08982077872858 7.86752137077896 -4.97499039746180
C 0.13238015380590 8.00198762226090 -3.60485192811819
C 0.25956970523773 6.87461949468463 -2.80397356481109
H -0.16123990745851 4.48193054502604 -5.17800372002038
H -0.37552516179746 6.48462430344250 -6.59868656375295
H -0.19204454095636 8.74812766527039 -5.59856424762523
H 0.20638582851395 8.98786932132940 -3.16009939474596
H 0.44216444362026 6.98205358037114 -1.74115065278369
C 2.05430260703218 1.36531640137093 -4.02567581462531
H 1.74198501378159 3.35200636653787 -4.81991549600488
H 2.59009861635707 1.02280365452093 -4.90380496203558
C 1.86018819248589 0.50273899680520 -2.95451958994256
C 1.18100844808168 0.94357396028291 -1.80730004059283
H 2.24160185590513 -0.51095509913299 -2.99746386288894
H 1.04895287090387 0.28607827838703 -0.96462949308685
C 2.96937642640086 1.91078983433120 0.78923969307276
C 3.49137063454776 0.71001006413501 0.30976971784442
C 3.38353136440815 -0.47831188144405 1.03269171891611
C 2.79052892525174 -0.43620933160815 2.29442056390438
C 2.27072549149207 0.74328992466027 2.82904879764771
C 3.18761596619035 3.18361287856969 0.01381882027342
H 3.98436093541270 0.70613262951695 -0.65606476221451
C 3.88247819388946 -1.77742608279644 0.45450485404631
H 2.74358219582581 -1.34031453663357 2.89164216583025
C 1.76204437175319 0.77924106450591 4.24246670966351
H 3.39773928265980 2.96263865073567 -1.03233817814035
H 2.32575665675475 3.84795717580577 0.04977832199873
H 4.04789521659249 3.72927966180665 0.41834616937319
H 3.10987420299162 -2.24349848056264 -0.16741641178638
H 4.76082219849933 -1.62152622538032 -0.17604095057853
H 4.14244062613196 -2.49076552279551 1.23899320293598
H 2.48658777153472 1.28430983156766 4.89171514303467
H 0.80916666090419 1.30314100321965 4.30791528238254
H 1.62544691365722 -0.23271398389019 4.62632534824657
C -1.36838506877048 6.17449724660575 3.46081522945935
C -1.76271266254504 5.07532724695733 4.22958394256403
C -2.59359978572617 5.22517026549561 5.33302812932886
C -3.04235473619888 6.48848845138036 5.71656129637793
C -2.66599674235194 7.59758298271653 4.97570259554218
C -1.85375240543570 7.42888473704970 3.85436913181844
H -1.44085739084248 4.08115851767583 3.94467041825482
H -2.89440596133008 4.34334614958461 5.88623813855875
H -2.98989598353470 8.59508518626896 5.23663440729936
H 0.04603930072432 6.84387665822164 1.96211750490913
H -1.07496908083382 5.58662418630041 1.44029028996829
N -1.54894995357889 8.65068866014773 3.08877728191037
H -3.68747839944900 6.60911904701316 6.57857886674998
O -1.54169031999411 9.71562024064691 3.69287611942874
O -1.33423846507890 8.53935371310183 1.88408108652554

14b(1) – global minimum

125

Coordinates from ORCA-job

C -2.12019530777102 2.84581625868012 -0.92230748248688
N -1.25188365545675 2.91316998365970 0.10394749805338
C -1.00185480745445 4.30143310342485 0.54697651521300
C -1.63212469003175 5.12236188250268 -0.57946167570802
N -2.41238973166227 4.10660131007018 -1.29727636341409
C -0.78292211059093 1.84547859813809 0.93057223976483
H 0.06733509016488 4.47902745443996 0.66196148504364
H -1.48709390239516 4.47134709800391 1.51282163112377
H -0.88545312051274 5.56388817191719 -1.24760278480951
H -2.28611120358644 5.91837637538891 -0.21600134463425
C -3.31427511741901 4.51267315399558 -2.35174464474492
Ru -2.88707269823547 1.19294724649225 -1.90619284184301
Cl -5.09404839171870 1.91133214463077 -0.96546119547733
Cl -0.79489455963636 1.13938553514609 -3.31070176256079
P -3.81659714106504 -0.35455335033591 -3.65478457963921
C -5.60396088926920 -0.97310851767335 -3.45891291681611
C -6.04550729089410 -2.13697676287389 -4.36148033321125
H -5.59801751637618 -1.32785213233101 -2.42110989844503
C -7.43440773496476 -2.63527692597629 -3.93433160626217
H -6.08818642694050 -1.80175380417833 -5.40091288377942
H -5.33501805357363 -2.96296582262323 -4.32837980096954
C -8.47209983350968 -1.50699147448798 -3.95957110576160
H -7.74980327818486 -3.45805320789548 -4.58435609599155
H -7.36764633460626 -3.04380126030298 -2.91821880011496
C -8.01146422329218 -0.30649754682651 -3.12523376070176
H -8.62592725646084 -1.18543375077155 -4.99793184542311
H -9.43885234508260 -1.87407271388300 -3.60048226489035
C -6.61918832544038 0.17793746969137 -3.54924312598477
H -8.73007875342359 0.51501403948386 -3.20886726387329
H -7.97742388670606 -0.58556530538220 -2.06462586200979
H -6.66682841125716 0.53897150041416 -4.58208045761102
H -6.30599233662960 1.00727233537509 -2.91841680863350
C -2.67673796015997 -1.87991581128712 -3.78247456029675
C -3.62581774857920 0.57257395408953 -5.30769553498585
C -3.87398062364131 2.07372459746548 -5.10323191711822
H -2.55531887398510 0.45437304284652 -5.50411594535461
C -3.54483503288736 2.86619005608105 -6.37334604467822
H -4.91364502334305 2.24995031692753 -4.80728717531351
H -3.23507617433714 2.42851448642157 -4.29198726635701
C -4.31362796771010 2.33319505119510 -7.58655041089790
H -3.76157602841285 3.92631174790343 -6.21772227301271
H -2.46744991057326 2.79037659904530 -6.56199507463644
C -4.07178935013665 0.83198249349901 -7.77761301261987
H -5.38807367755196 2.50615215429914 -7.44026389864255
H -4.02617777071024 2.88379596668989 -8.48764492002642
C -4.42358470788344 0.04469567088205 -6.50681717264291
H -4.65966667037640 0.45438360869629 -8.62037610058736
H -3.01706150942488 0.66282397509798 -8.02718290616643
H -5.49481796149679 0.15663166895000 -6.31193476834738

H -4.24125465266398 -1.01905262676452 -6.66209420627571
C -2.50646208505052 -2.51321674589163 -5.16933798081976
H -1.71663143306663 -1.43387155376006 -3.51051558504985
C -1.42368104795518 -3.60313002697568 -5.11971658674089
H -3.44971954813599 -2.95619249056581 -5.50705145813649
H -2.22182970368782 -1.75709452869661 -5.90459917162533
C -1.72440247235406 -4.66020881781678 -4.05119250320317
H -1.32491909829301 -4.07134803225099 -6.10443124847675
H -0.45903637591417 -3.13054283793180 -4.89692684248187
C -1.92859113789471 -4.01315013681411 -2.67717322057449
H -2.63359542038656 -5.20930554555506 -4.33201428227898
H -0.91431599591531 -5.39530462484517 -4.00945385574818
C -3.02494538238159 -2.94201709643401 -2.73107018052002
H -2.18152668627640 -4.76918583254548 -1.92817365936215
H -0.99181650074291 -3.55098935325557 -2.35058271236478
H -3.97494592951270 -3.42392766988882 -2.98179000439576
H -3.16126135696493 -2.48889810033652 -1.75122506434414
C -2.37541955651139 -0.20970517536850 -0.78238515344383
C -1.06584949138843 -0.86301760689201 -0.82038102430506
C -1.00316760844440 -1.90385871862354 0.05590347022640
C -2.29907557147956 -1.97575885592806 0.77968642467985
C -3.15278180707213 -0.97405662609587 0.24601781120318
C -2.72027554361818 -2.76524036722287 1.83521785326556
C 0.12793554278494 -2.82027555432383 0.19746706393528
H -0.29495573866226 -0.57174411045030 -1.51168304734250
C -0.04704227902760 -4.15264992738483 0.60640681684456
C 1.03775408037951 -5.01771229338563 0.70834985444341
C 2.32151568782683 -4.57339394799167 0.40355576022206
C 2.50935523247562 -3.25845080691491 -0.02379077640737
C 1.42782864355360 -2.39537689466483 -0.12879140919401
H -1.04246184961201 -4.52376239457081 0.80587166217525
H 0.87747823901775 -6.04329472896979 1.02085365500309
H 3.16761077997928 -5.24532659174789 0.49007604041127
H 3.50368101299753 -2.90608355034515 -0.27433278574339
H 1.58210598112196 -1.37501302405120 -0.45197416003470
C -4.02193619590928 -2.59493228255407 2.33610581866878
H -2.06106659596467 -3.48807509162065 2.29740007211810
H -4.35830219510172 -3.20925365244559 3.16414135759826
C -4.87491524146854 -1.65367438836196 1.77909017920499
C -4.44220134430672 -0.83041491448594 0.72683051650668
H -5.88161933870786 -1.53933478301525 2.16418654653009
H -5.09351186597246 -0.07626895641895 0.31032672959506
C 0.53576268264495 1.39972132134676 0.76281436920348
C 1.00908525416103 0.41641946177686 1.62733723341274
C 0.19560350551846 -0.14920163124557 2.61141939231197
C -1.10299847935965 0.33472176387380 2.76031081343910
C -1.60886883191420 1.34858927792440 1.94706860457238
C 1.39372596875775 1.93443486198031 -0.35293804343729
H 2.02571207665587 0.05752070527944 1.51069007002414
C 0.69569098789279 -1.29092531917106 3.45638311003147
H -1.74767079795798 -0.09436512999290 3.51992066772703
C -2.99471538038775 1.89244134521918 2.16859366185872

H 2.36885450603120 1.44431627990465 -0.35550793078022
H 0.91511432808247 1.77110899030145 -1.32243789691997
H 1.56651165303156 3.01087747419833 -0.25607915954827
H 0.53245793721927 -2.24298394691899 2.93976817301431
H 1.76785432342889 -1.20705101215565 3.64785965819154
H 0.17545158784311 -1.33627425430969 4.41480345711967
H -3.57218723349527 1.94684816834502 1.24543840650066
H -3.54076507804272 1.26315027605751 2.87177147899606
H -2.95268450895729 2.90393187370481 2.58969828045764
C -2.61933237955544 5.07552369767859 -3.57305365428874
C -3.22314342489880 6.10649060379089 -4.29850971279658
C -2.64105436367831 6.59272610333740 -5.46203139276986
C -1.43995301555821 6.03506351996173 -5.88509964279454
C -0.81210062908245 5.01632459697639 -5.17824028099777
C -1.40591486367745 4.54131240302665 -4.01634415118979
H -4.16039302214374 6.52867912475674 -3.95318119677839
H -3.09258999870909 7.38563742531037 -6.04122558303494
N -0.82038538755203 6.53513674835979 -7.12847801439700
H 0.11434963692462 4.60142308693454 -5.54800049516423
H -3.93594438724950 3.65573967195387 -2.61626803012844
H -4.00904985315993 5.25635797871114 -1.94626554301996
H -0.94397434291695 3.72021092995734 -3.48226438652231
O 0.22938739801847 6.01761658702953 -7.48666551606336
O -1.39537278241456 7.43484761554340 -7.72786834170926

14b(2)

125
Coordinates from ORCA-job
C -0.18928918966509 0.82195927381262 0.97672544955149
N -0.68281612168032 0.56839086955170 2.20270024512392
C 0.15545115304408 1.10153646007358 3.29215229711810
C 1.13959071476907 1.98825271631188 2.52850677000057
N 0.93613612388922 1.56484703934645 1.13173856030619
C -1.90786269843803 -0.10934997772397 2.50419210054048
H 0.65152594241154 0.28075811976481 3.81761995832744
H -0.45774121870862 1.65065899315213 4.00800476879296
H 2.17639660472924 1.84415900174937 2.83766121533994
H 0.89843756426991 3.05400654685821 2.62249151518511
C 1.59083267788680 2.33459093964143 0.09034385634679
Ru -1.13658334206715 0.26740848356821 -0.79127811349538
Cl -1.39265801761473 -2.09800333596618 -0.13557879631843
Cl -1.44014748911257 2.69770695626911 -1.24085956809178
P -2.60425895585835 -0.35384990170227 -2.77012839498963
C -4.10715186768885 -1.36859516732962 -2.18757806926514
C -5.26908700905958 -1.52485157651090 -3.17978916762245
H -3.65207537465588 -2.34956437369442 -2.01142298116819
C -6.33085421788979 -2.48216972043636 -2.61775797778999
H -5.73258165042784 -0.55002130944145 -3.36069793905520
H -4.92040011380129 -1.88776289900025 -4.14448549165921
C -6.84374362093957 -2.01030668714863 -1.25324176961914
H -7.16037164728657 -2.57563572733883 -3.32625698606792

H -5.89035529618266 -3.48188309561478 -2.51332367600425
C -5.68947252052424 -1.80557014780259 -0.26642926469907
H -7.37994005646900 -1.06064914885432 -1.38192394976666
H -7.56764745356974 -2.72689490435335 -0.85067785083457
C -4.62018738642070 -0.86189155218232 -0.82846884811759
H -6.06479945948350 -1.41240050912376 0.68126239747849
H -5.22029432546820 -2.77106752222419 -0.04050296112625
H -5.04276675847787 0.14139425717224 -0.93704271824159
H -3.79510621290459 -0.79638258950107 -0.11906424796558
C -1.70262756773623 -1.41232136783761 -4.08977688815136
C -3.19420322519612 1.21241271254412 -3.68900160784601
C -4.28560656948864 1.95236722293105 -2.89984965577746
H -2.29722346261558 1.83579690501898 -3.62733141855612
C -4.53758374697899 3.34017244562369 -3.50034634858583
H -5.21703107015992 1.37839009221078 -2.92421263709460
H -3.98474903661138 2.05640454036468 -1.85837896800544
C -4.90790594755461 3.24716338330132 -4.98423413052954
H -5.33043052187650 3.84739775194578 -2.94087490287409
H -3.63062872116669 3.94277330101171 -3.37877497769066
C -3.84263115517616 2.47653521790113 -5.77093245229829
H -5.87166773903275 2.73078481247834 -5.08412897073099
H -5.04191637330861 4.24695944725862 -5.40949421935809
C -3.57734254028295 1.08735606068466 -5.16842411395940
H -4.14017695183744 2.37324844024646 -6.81967168267615
H -2.90611697877065 3.04859158180072 -5.76335979069813
H -4.47362171002422 0.46660636060580 -5.27575794679616
H -2.77951437807277 0.60225011030913 -5.73598213923375
C -2.56836667135620 -2.11533338005230 -5.14851508963198
H -1.08421387191949 -0.65693147554716 -4.59005808894231
C -1.67662178651362 -2.71400096771433 -6.24813120283546
H -3.12682728667309 -2.92405459117374 -4.66535987740366
H -3.30095829253551 -1.44705611344778 -5.59776465064653
C -0.64556968393060 -3.69283351338111 -5.67636641628305
H -2.30100360019989 -3.21239753377146 -6.99726187246320
H -1.15641037519899 -1.89757901597505 -6.76573873038185
C 0.15937314216616 -3.04880259089470 -4.54322476646767
H -1.16997047296138 -4.57385833430670 -5.28421982702884
H 0.01914199194490 -4.04938381561612 -6.46936866478539
C -0.75988105027560 -2.45350116051060 -3.47271865336104
H 0.83175000243023 -3.77992064252871 -4.08395532909452
H 0.79707948264525 -2.25243039979334 -4.94822303217296
H -1.35349059549160 -3.24717199140722 -3.00735133304054
H -0.16909462729804 -2.02676660713057 -2.67222336205306
C 0.43473338399415 0.06428419006000 -1.76441901638443
C 0.72935518601997 0.71772594492717 -3.03792225178439
C 1.91407190344318 0.27009674706273 -3.55126893822342
C 2.47521362232137 -0.73535045754940 -2.61011860503632
C 1.58713047653613 -0.83970964972017 -1.50969435819854
C 3.59000497984237 -1.55149887784131 -2.69015603026389
C 2.51004483338849 0.68958773606639 -4.81871117401108
H 0.10461707885016 1.48677478779283 -3.46482188680445
C 3.90152661871249 0.76940775583998 -4.99284947275582

C 4.44189546540725 1.20189929685685 -6.19924686557026
C 3.60706404561604 1.54997244435191 -7.25901679659893
C 2.22319923114774 1.46883096381594 -7.10312592800702
C 1.68058321483051 1.04532631410396 -5.89686989312794
H 4.56650310731716 0.52823726682059 -4.17624267941763
H 5.51876458381898 1.26783760133205 -6.30579420487825
H 4.03044608870699 1.88010910437173 -8.20075465641837
H 1.56759489518602 1.73054513203444 -7.92591130425222
H 0.60566350600726 0.96581138643641 -5.78439325880468
C 3.84162276285046 -2.45627981210716 -1.64466321517929
H 4.25842015604076 -1.51499146757169 -3.54056808252856
H 4.72206561705474 -3.08700161408374 -1.69097644294893
C 2.97445382801643 -2.55212384368884 -0.56299140643111
C 1.82865625872699 -1.74596728705740 -0.49202564408267
H 3.17759353463589 -3.26514582778731 0.22829892507593
H 1.12280706108890 -1.85087203340689 0.31913869025876
C -1.86944305420926 -1.42766439047545 2.97780844621718
C -3.07509406298242 -2.04990265892881 3.28849915109612
C -4.29941226931293 -1.39583473920499 3.14646025377559
C -4.29864448228180 -0.07725921940910 2.69595264725539
C -3.11461660845745 0.59168868688502 2.37761213688076
C -0.57096739790014 -2.17124558590667 3.14690813299432
H -3.05888516436783 -3.07955001915804 3.63094204283028
C -5.58717916644131 -2.11099344981632 3.46846261310289
H -5.24025204017361 0.45223936602762 2.59403719922087
C -3.14826314457843 2.03498538702911 1.94519923170086
H -0.71044407411091 -3.23060087921743 2.92996556424592
H 0.19329573323109 -1.79446618703435 2.46889289131030
H -0.19881005490839 -2.08431108233805 4.17501081786084
H -6.44427994781059 -1.43856103291676 3.39918576465859
H -5.75441636573117 -2.94052120880264 2.77483104817627
H -5.56488526311931 -2.52931939311423 4.47885821876313
H -2.49442396408285 2.64980129463291 2.57138041238742
H -2.81084004524435 2.18152424336848 0.91713172763808
H -4.16156444988781 2.43171409560673 2.02593765340076
C 2.96984764980918 1.86387636007948 -0.30296241547999
C 3.58172929712316 2.47923831953844 -1.40138787868995
C 4.79651281010278 2.02206900799259 -1.88461420308375
C 5.40094167258177 0.94234054237102 -1.24746414540202
C 4.84483275106315 0.34829259098620 -0.12176209968999
C 3.62254588257345 0.81449636421501 0.34200014731487
H 3.07690941139357 3.29286836441316 -1.90937579426643
H 5.26164849903001 2.45928638801979 -2.75547014720058
N 6.61349486556451 0.35752056721668 -1.83234428703316
H 5.34290096602813 -0.49299406298103 0.33584803428720
H 1.64769672746140 3.37636395425484 0.43000119551220
H 0.94570734288739 2.35106544896762 -0.78490774203189
H 3.13865101865002 0.31250826054781 1.16808885377425
O 7.19869942792796 -0.50983382311808 -1.19647085707015
O 6.95779612576508 0.75408389833950 -2.94354210194190

14b(3)

125

Coordinates from ORCA-job

C -0.29184501890450 -2.03607884373974 0.82986481158885
N 0.98986530809401 -2.17112951255525 1.19210859723561
C 1.23253857132295 -1.78325146420348 2.59876702366751
C -0.16869934277637 -1.37997380025947 3.08681654746659
N -0.99466487980465 -1.60440451145591 1.89331300592523
C 2.08124451701800 -2.59624956087064 0.36905232191986
H 1.94972701672871 -0.96012961384713 2.63801688185563
H 1.64498465367451 -2.62649476645701 3.15549850546831
H -0.22384092497140 -0.32837155724563 3.38476673071457
H -0.52128728881679 -1.99380569229950 3.91906284594828
C -2.37109827975001 -1.16197487120865 1.85721376805009
Ru -1.24668631476706 -2.34875231632750 -0.96659343538588
Cl -1.75093495022692 -4.66797269526935 -0.17336245545202
Cl -1.23377277253384 0.17008552028964 -1.08929569040843
P -3.01290514653922 -2.54119946553651 -2.75858590694954
C -4.59440510029192 -3.27630710001954 -1.99797680518147
C -5.88609025992068 -3.14998678615577 -2.81670183618377
H -4.32100500218956 -4.33477106777568 -1.92823312734036
C -7.02643237769852 -3.92492706376670 -2.13807670141827
H -6.17145834509596 -2.09675324193534 -2.89274743483836
H -5.74468436389246 -3.51158006972110 -3.83501599279486
C -7.24359321501397 -3.45741976850833 -0.69377000889399
H -7.94804965768323 -3.81098345912473 -2.71835725093998
H -6.78322839139917 -4.99493713034899 -2.13855821642589
C -5.94785667567713 -3.54713015046473 0.12058674958624
H -7.59200421554471 -2.41644776946704 -0.70359655035370
H -8.03297094663562 -4.04826048055142 -0.21906131238532
C -4.80596469128870 -2.78301988320849 -0.55766215547027
H -6.10173662346694 -3.15939195650454 1.13154700855986
H -5.65200666925123 -4.59722194630028 0.22908181973880
H -5.03034787826282 -1.71223359425059 -0.55635971070884
H -3.89053091305508 -2.93850454565021 0.01391011708963
C -2.52411986688914 -3.71303020061756 -4.18525295468252
C -3.32161462780823 -0.80258036417868 -3.49302792688107
C -4.22650536444931 0.04496083154219 -2.58555373884023
H -2.31876836986847 -0.37603172182597 -3.40836025506613
C -4.21367148763060 1.51093180335225 -3.03283188377446
H -5.25285992421269 -0.33358076042173 -2.62183993378526
H -3.87799560063831 -0.01684734113700 -1.55600357689496
C -4.62982483199461 1.65033882011386 -4.50007107208243
H -4.87579330255607 2.09933970817582 -2.39003108911334
H -3.20160341721919 1.90656100134442 -2.89313452387125
C -3.75063654259695 0.78019402073540 -5.40307237575060
H -5.67779854583997 1.34113813467802 -4.61171058272851
H -4.57662454188550 2.69725568512989 -4.81600202851384
C -3.75159320697248 -0.69161874624510 -4.95986186816517
H -4.08035384026401 0.85047170989750 -6.44477658747270
H -2.72037025882629 1.15796216981501 -5.37161408495180
H -4.75428055680924 -1.11197634464060 -5.10038044702383

H -3.07093609599325 -1.25364458807226 -5.60239768627081
C -3.53687320209539 -3.98076547036423 -5.31283004195559
H -1.65629476011132 -3.19311228469619 -4.60818450936796
C -2.83978083559511 -4.73892002985351 -6.45591472905157
H -4.34704261058257 -4.60429623622618 -4.92065612995452
H -3.99296723185661 -3.07224211500855 -5.69579583014109
C -2.23487601211566 -6.06314414132837 -5.97181232685527
H -3.55158733467897 -4.92114401311392 -7.26753771518869
H -2.04567079375911 -4.10368856206019 -6.86916258239181
C -1.33329743324777 -5.85685059198499 -4.74917461270770
H -3.05070208154134 -6.74643565500701 -5.70270745333657
H -1.67860158619509 -6.54379088557079 -6.78338222240459
C -2.05476729886840 -5.07238640903184 -3.64702101643933
H -0.99444346035929 -6.81943885948405 -4.35606837526786
H -0.42960123822792 -5.30755214648312 -5.03962396317449
H -2.92811355494476 -5.64133556854642 -3.30793545355443
H -1.41798175591036 -4.95638785757957 -2.78057836744810
C 0.13848248588218 -2.59446596485200 -2.19184722291845
C 0.58821617140041 -1.54889903672772 -3.12029471839784
C 1.55270509419232 -2.02266716149806 -3.95603573064387
C 1.77246447672316 -3.45724868427135 -3.64973924529264
C 0.93801209074812 -3.80472777271260 -2.55803550625502
C 2.55361928058229 -4.41071040578369 -4.27956208525482
C 2.28310168923691 -1.23824272296385 -4.95202130572041
H 0.23589353967579 -0.53245922100846 -3.07121102743960
C 3.65260039016118 -1.45074331283482 -5.17929202247110
C 4.34948901996340 -0.66937707247304 -6.09505369195793
C 3.69228249659193 0.33243446509563 -6.80650200141797
C 2.33272253353941 0.55494350445943 -6.58889222038137
C 1.63541656429506 -0.22029236356343 -5.67080154620027
H 4.17446564089927 -2.20836194241804 -4.60940721494544
H 5.40870873048232 -0.84077945768693 -6.25167374542455
H 4.23499821211823 0.93636288762585 -7.52475601964165
H 1.81503259654024 1.33201953555457 -7.13987048078124
H 0.57626509212702 -0.05553620640188 -5.51153692493120
C 2.54589725197463 -5.72450698637387 -3.78488275235157
H 3.15312726702949 -4.15884227850385 -5.14572211112037
H 3.15308286596548 -6.48164876576459 -4.26843086560218
C 1.76287122645948 -6.05851020526993 -2.68872385969317
C 0.94713600000658 -5.09916635111356 -2.07090066652761
H 1.76652979350743 -7.07439782309727 -2.31073371994241
H 0.31473535698479 -5.37085488531237 -1.23907526268836
C 2.73867171668354 -1.65720540094325 -0.43616092815739
C 3.80942432893471 -2.10077436062262 -1.21361623392318
C 4.25005514332823 -3.42237263641846 -1.17440590636350
C 3.59222598079490 -4.31929834391516 -0.33144307613528
C 2.49997398871076 -3.93147166610207 0.44050854863648
C 2.32710601507851 -0.21045278962088 -0.45418558741961
H 4.30955026657151 -1.39048245653263 -1.86419704060762
C 5.39017009347557 -3.88677343668978 -2.04272723887955
H 3.91492911439105 -5.35470198361904 -0.30024652148407
C 1.76449982696282 -4.93081992535490 1.29424538757826

H 2.84952853180668 0.32350999298122 -1.24914028913910
H 1.25459406960693 -0.08560127650917 -0.60714737272746
H 2.58027124656332 0.27858522306918 0.49419878810200
H 5.94103428189375 -3.04152087517900 -2.46031039554460
H 5.01302767938455 -4.48964721723762 -2.87417167145433
H 6.09325126053306 -4.50632824952635 -1.47899954808383
H 0.68856708759788 -4.89515614609635 1.10735253964021
H 2.11572186543204 -5.94242163721968 1.08337986524625
H 1.92268590706247 -4.74666780235495 2.36291938335567
C -3.33083247161396 -2.01376917143832 2.65683538308089
C -3.14909439540590 -3.39430062974478 2.76757733119017
C -4.08928966761433 -4.17552654417674 3.42747205517172
C -5.20580917305519 -3.55940832288092 3.97973817813355
C -5.40851069681308 -2.18677823983420 3.89126528923878
C -4.46020871884112 -1.41950356508305 3.22814772660786
H -2.29816283952757 -3.86453876030903 2.29202871291040
H -3.97878974213639 -5.24792346574028 3.50482511414124
N -6.21551195318526 -4.38605108126651 4.66988227634702
H -6.29123862185163 -1.74996275911082 4.33611118926156
H -2.68855241848363 -1.12619173302009 0.81077402605891
H -2.41802229052254 -0.12207804515335 2.19879745334497
H -4.60238611888183 -0.34682209848465 3.14695474543077
O -6.01050389144449 -5.59018515530032 4.74293411237031
O -7.19912952135838 -3.81635503923658 5.12626145349796

14b(4)

125
Coordinates from ORCA-job
C 0.01778101565181 1.02705535140963 0.59588047774334
N 1.22003509729328 0.84357633808305 1.15718397469343
C 1.41039693789367 1.68910880951144 2.35586442163480
C -0.01204241662211 2.19262733949641 2.63027342206015
N -0.68058338782440 1.88878432249318 1.35899147417747
C 2.27850666809254 0.00281661931231 0.68730578273763
H 2.10196485628802 2.50459543774562 2.12485163462087
H 1.82416826620014 1.10225111911670 3.17631732659463
H -0.05262873049500 3.26060321553563 2.84727934553664
H -0.49779945244918 1.64621448081117 3.44727508487237
C -2.08132843616331 2.19723795792197 1.15071945444321
Ru -0.86619679465525 0.30432398498982 -1.10483473597764
Cl -2.01715010582368 -1.35371314489230 0.37893530359629
Cl -0.24108564434398 2.53030336187838 -2.09880798681198
P -2.48052004248729 -0.19379922618578 -3.00193225311416
C -4.26822139169462 -0.45671148670496 -2.39977933770868
C -5.34742612866725 -0.44914429144092 -3.49265139479589
H -4.20859126110380 -1.46228004470176 -1.97192229286219
C -6.71668100805660 -0.81928070623566 -2.90250808481932
H -5.41009671268956 0.54646546847755 -3.94126764661559
H -5.09438326623068 -1.13790797930840 -4.29869461370123
C -7.10002226660028 0.10799443381181 -1.74488930397334
H -7.47926523177786 -0.78700689401670 -3.68757211203058

H -6.68118586491400 -1.85466925599583 -2.53974030383771
C -6.01267892130792 0.12244464084552 -0.66507073328199
H -7.24076746247647 1.12588899058472 -2.13087990446049
H -8.05898571271433 -0.19922469785200 -1.31581685671912
C -4.64081539049876 0.48747453374352 -1.24616133893761
H -6.27600611910076 0.82649961980626 0.13114005037706
H -5.94589408317397 -0.86833216161355 -0.19950430369610
H -4.65856562477215 1.52399755049992 -1.59451527071519
H -3.88775564892101 0.40488694506033 -0.46195622253384
C -2.00354903396463 -1.83825615182141 -3.86416227637979
C -2.41789068830845 1.25493494592645 -4.24586707749811
C -3.19378878222364 2.46274580930835 -3.69327560948126
H -1.35901286400289 1.51996613703508 -4.19658038924769
C -2.92613659486433 3.71358267129762 -4.53638117290622
H -4.26803298753940 2.25199527152391 -3.70277931918914
H -2.89696928547494 2.65179528438445 -2.66200327459071
C -3.28101458277833 3.47298633441488 -6.00667886349596
H -3.49967657946825 4.55631765854906 -4.13712737183378
H -1.86729637844422 3.97795208125751 -4.44460181014949
C -2.52593629658839 2.25937558367368 -6.55608460190996
H -4.36194146137956 3.29802969434080 -6.09395186696770
H -3.05739593841667 4.36074349824669 -6.60690436041163
C -2.76659173667364 0.99433945487979 -5.71520990882033
H -2.80904856049387 2.06735504012433 -7.59628552942771
H -1.45100555170487 2.48105173643230 -6.55641772510224
H -3.80988484698354 0.67593362122786 -5.81705759653523
H -2.14208221623587 0.19281892875962 -6.11131716548587
C -2.77545926381199 -2.31431226555940 -5.10761502748437
H -0.96438757158876 -1.64308608478527 -4.15312521562495
C -2.07556756904988 -3.54511382670222 -5.70987828282441
H -3.79084605300682 -2.59842032894279 -4.81485571865765
H -2.86892834908344 -1.54169266754683 -5.86655333537414
C -1.97111753044459 -4.69044875487941 -4.69704171838235
H -2.61480908136448 -3.87410916336367 -6.60417757549152
H -1.06922441047111 -3.25480290142277 -6.03754261171906
C -1.31608045121197 -4.22077398270855 -3.39377153189399
H -2.97979283971083 -5.06361932431844 -4.47775405552502
H -1.41277581175607 -5.52816048568843 -5.12716052069801
C -2.01621127560182 -2.97765752264806 -2.83197671307462
H -1.33003241056450 -5.02032968044910 -2.64699108994600
H -0.26113191286521 -3.98399472018330 -3.57190915938042
H -3.05787094271404 -3.22727028215235 -2.59643864267954
H -1.55989671474992 -2.67200694352701 -1.89751967360886
C 0.51899248238009 -0.65078073428320 -1.90767790747430
C 1.30020605047698 -0.15072088962121 -3.04572263109258
C 2.20081391762886 -1.08275154618031 -3.46363681071422
C 2.03362428379814 -2.29979033551849 -2.63030140884850
C 1.02917274410917 -2.03470378792409 -1.66697887495498
C 2.62101990616509 -3.55088621186337 -2.71633368635937
C 3.19260632801352 -0.89504419234372 -4.52234782227920
H 1.20496109065080 0.85307170276468 -3.42458688438778
C 4.48245997097244 -1.44035428333672 -4.41693618809741

C 5.43348705636576 -1.20914893118311 -5.40556000319944
C 5.11318843266793 -0.43931488534096 -6.52198665204039
C 3.83471684998515 0.10580377638568 -6.64010060178125
C 2.88542348168352 -0.11731317796713 -5.65089408875675
H 4.74719670006289 -2.01558598773224 -3.54010948430506
H 6.42821175778277 -1.62775590477620 -5.30218550993002
H 5.85345798909982 -0.26372589509234 -7.29425601475644
H 3.57752514979271 0.70291367503089 -7.50768187899304
H 1.88932925349651 0.29774281064773 -5.74827260624364
C 2.23797555358444 -4.54079659309526 -1.79761375807894
H 3.35274898809412 -3.77428034582620 -3.48279313542823
H 2.69040326033383 -5.52462272075281 -1.85327439996153
C 1.27819154654507 -4.27221445553989 -0.83031310075672
C 0.65949914702048 -3.01558907937509 -0.76435894781723
H 0.98783253423880 -5.04503571062364 -0.12770667816240
H -0.11138206748401 -2.81667905552226 -0.03465229862008
C 3.17918270634372 0.48096552093480 -0.27082297254708
C 4.20026450840981 -0.37500601273884 -0.68889429473784
C 4.35045870772577 -1.65515272972107 -0.16068082501682
C 3.45359604649334 -2.08282320495676 0.82050409450403
C 2.40832409104891 -1.27328868453864 1.25421849642597
C 3.06729078746497 1.87177619262237 -0.83185734466129
H 4.89044035964953 -0.02805028296622 -1.45063023268250
C 5.44516790154896 -2.57203449450162 -0.64147203267193
H 3.54817546192319 -3.08250397857498 1.23081986922963
C 1.41686452189555 -1.76599397941492 2.27482980488402
H 3.75161016899198 1.99847741856271 -1.67223669286035
H 2.05813554884937 2.09528940501971 -1.17766023436448
H 3.32818912986976 2.61998218292564 -0.07497175242236
H 5.01962041801598 -3.46974478027049 -1.09982062432865
H 6.08061370027406 -2.07863258931702 -1.38001958001604
H 6.08199344819396 -2.89839248264595 0.18612341027897
H 1.51360061930122 -1.22557109924162 3.22283202746497
H 0.38937942159395 -1.63196056818216 1.92892762726612
H 1.57613773307230 -2.82507577854257 2.48164015038375
C -2.36812511510053 3.67659676458473 1.25709747002535
C -3.39697671009118 4.14087923015173 2.07797159908829
C -3.68169291490685 5.49996645020253 2.15756870791313
C -2.91536894338475 6.38125315517964 1.40647323450904
C -1.88385450964990 5.94551402631249 0.57993769830831
C -1.61262273791696 4.58636546646128 0.50811383285475
H -3.98151785864737 3.43606061248437 2.65912384542013
H -4.47347196040814 5.88470742819140 2.78488869540860
N -3.20763108632266 7.82764638722231 1.48768431806455
H -1.32140250754114 6.66798285943530 0.00586282635672
H -2.70495274056369 1.62541244280599 1.84668460219267
H -2.35222487148410 1.86714092974082 0.14419522740861
H -0.83389570237906 4.21395604891395 -0.14784783419720
O -2.51229512963855 8.58461940259242 0.82369713506627
O -4.12758406241069 8.18077220346752 2.21416485579463

14b(5)

125

Coordinates from ORCA-job

C -1.10843731084181 0.37628375332760 1.09475989232499
N -0.44645981239523 -0.57897086964232 1.78149141273893
C -0.12575721244937 -0.19513938395392 3.16941591402053
C -0.73051767102653 1.21114406269201 3.26443801097560
N -1.26739974882964 1.42759670696945 1.91059233985787
C 0.12551954324139 -1.77420420525665 1.24130459363225
H 0.95680096964845 -0.20961980677885 3.32250236003733
H -0.58135905044002 -0.89672123378641 3.87001999621996
H 0.01055103354682 1.97765500023149 3.50723653461976
H -1.54101641516721 1.27004289122638 3.99568164101724
C -1.71068558654934 2.75263519169975 1.52883094988011
Ru -1.90336785700754 -0.08337425586016 -0.82284423266474
Cl -3.94393955187801 -0.71249474948031 0.60339620372224
Cl -2.70425022815188 2.14591274700810 -1.40451016843707
P -3.10665113770136 -0.99264380991545 -2.83591639032223
C -4.99688192239917 -0.73909606877454 -2.96172345834551
C -5.58327465792722 -0.92659850040075 -4.36965281531083
H -5.37209942163572 -1.53390361879625 -2.30631212845607
C -7.11720185351260 -0.87723187617096 -4.33239745193208
H -5.22064685090933 -0.11985912463057 -5.01482936456704
H -5.25849724925486 -1.86214015392982 -4.82253141685370
C -7.61882454771082 0.43093830561851 -3.71277835436237
H -7.51446107958713 -1.00185094734176 -5.34539609655026
H -7.48840579845733 -1.72557802221288 -3.74256089721225
C -6.99886141300291 0.65276176651270 -2.33005204405974
H -7.34092870836334 1.26539509655375 -4.37044388555520
H -8.71196727635360 0.42819205078077 -3.65060520143231
C -5.46700851480020 0.59499970543717 -2.36797552875909
H -7.31610449446862 1.61692855214257 -1.91994648443440
H -7.36540461493021 -0.11626028441053 -1.63811423052654
H -5.07848041313746 1.42331559707535 -2.96844170568751
H -5.06601748620340 0.71821810031420 -1.36552193912234
C -2.85956736754803 -2.89298734125078 -2.79615527507366
C -2.22093303406522 -0.46626781605641 -4.44295993381274
C -2.39801076214505 1.02648419410133 -4.74771512053347
H -1.18201520659523 -0.61165825109286 -4.13729306538404
C -1.41205370111910 1.46444608164653 -5.83787555775540
H -3.42418788774974 1.21280545735193 -5.08581152640825
H -2.25308680378518 1.61672457856912 -3.84394793683067
C -1.54839042289432 0.60863141877298 -7.10220817283428
H -1.56524956794141 2.52215952293801 -6.07501477525646
H -0.39088823530383 1.37811613533105 -5.44628613959302
C -1.41925731396136 -0.88483296068022 -6.78061104810794
H -2.53058666719837 0.79211611565517 -7.55756515237900
H -0.79846208505160 0.90173503019031 -7.84381356895559
C -2.42267007153206 -1.32063790989711 -5.70035963002046
H -1.56162539451908 -1.48461005217850 -7.68540394627144
H -0.40292465796483 -1.09088128685241 -6.42028120656640
H -3.44053893765826 -1.20686995113508 -6.08350576475091

H -2.27334738169850 -2.37968006259045 -5.47926766147760
C -3.89301168338890 -3.73840082770643 -3.55430025358123
H -1.88877585162991 -3.02790363916023 -3.28562733953698
C -3.54821353431888 -5.23197225787210 -3.47803649566609
H -4.88545727787570 -3.58024500405933 -3.11950365682339
H -3.95000237653788 -3.43515220742070 -4.59983266352060
C -3.43321622662869 -5.70594629667954 -2.02741477842064
H -4.30517372972076 -5.81305695143006 -4.01397888665007
H -2.59429167556166 -5.40636670473252 -3.99377618292056
C -2.41260721642234 -4.86220948466434 -1.25821120301191
H -4.41277768786390 -5.61604358341744 -1.54024125675664
H -3.15829598311234 -6.76491153671700 -1.99102033829597
C -2.74143205515937 -3.36888121205191 -1.34191458346643
H -2.37790735833125 -5.17234305782566 -0.21064595334089
H -1.41062014010665 -5.03851635184703 -1.67143358083779
H -3.66800994659175 -3.15291277579664 -0.80303771615886
H -1.97121248554974 -2.79501008061215 -0.81471838368869
C -0.31176345377066 0.22697816983033 -1.81609911414860
C 0.25351743268773 1.51280796783848 -2.21154108551162
C 1.33313988938406 1.35909113082399 -3.03164111467873
C 1.55457491335083 -0.09645186972319 -3.23453517636894
C 0.57994118160481 -0.78316410152859 -2.46675491585353
C 2.43108958040632 -0.79356199201949 -4.04720951545255
C 2.11769676054818 2.44878962608257 -3.60604232818223
H -0.14984508018168 2.45509443712461 -1.88547024067659
C 3.51077535341923 2.35433573564964 -3.75008823770024
C 4.24605071959245 3.43410902847667 -4.22608604604601
C 3.60398589824398 4.61719898693406 -4.58426941803288
C 2.21847518455387 4.71637561321209 -4.46779701041326
C 1.48228609035608 3.64681674896751 -3.97643228319029
H 4.01926429329632 1.44909476774375 -3.44214491494385
H 5.32475141567888 3.35830270666288 -4.30296683174975
H 4.17940534885030 5.46368616040756 -4.93896667446402
H 1.71699913945626 5.63801726513425 -4.73592679472265
H 0.40513522822784 3.72232624646414 -3.88512194746931
C 2.36406572465462 -2.19904712522585 -4.06343273411668
H 3.14460287139478 -0.27590516086187 -4.67678454337910
H 3.04562887323131 -2.75717097663339 -4.69567917533332
C 1.43609949928547 -2.87549290132073 -3.28230537487447
C 0.53288772373931 -2.16412511600667 -2.47584231996901
H 1.40155495882192 -3.95912028401830 -3.29770997096395
H -0.17928143856081 -2.69889551046290 -1.86494479353212
C 1.39254532901151 -1.69476663431741 0.63569654846784
C 1.99194625837492 -2.87072672396657 0.19683691147028
C 1.37217992743640 -4.11219642542696 0.35533826993165
C 0.13437469879103 -4.16024091429495 0.99203159897436
C -0.50567152685076 -3.00744687115503 1.45729108204533
C 2.09391534638551 -0.37447002459191 0.46402885612156
H 2.95586914287920 -2.81425419038972 -0.29717180403746
C 2.03274187556429 -5.36379123901661 -0.16293664373971
H -0.34788109786890 -5.11944101047038 1.14200675944810
C -1.81311998495270 -3.09908263178182 2.19537126246442

H 3.00598458237344 -0.49882935236471 -0.12000017874019
H 1.46175705275199 0.34892687088392 -0.04975389661680
H 2.37008596219732 0.05847547090324 1.43163401734396
H 1.44021477947602 -6.25111175103073 0.06662483837400
H 2.16182368794396 -5.31317941094763 -1.24853506252931
H 3.02690069891068 -5.49617170326908 0.27335949607536
H -2.54810964177305 -2.38591220022714 1.81434522656738
H -2.22727211563799 -4.10598053675042 2.11626634932721
H -1.67305372456207 -2.88731753139801 3.26105681306444
C -0.64630977745243 3.54944488863981 0.79581501923952
C -1.02269529678764 4.69236959849041 0.08146581970293
C -0.08942181756388 5.42132293609646 -0.64012775835391
C 1.23642851437344 4.99608727429330 -0.64370376664052
C 1.64197937992251 3.86962532428232 0.05755817147946
C 0.69211475488104 3.15371263537486 0.77591022443523
H -2.06457761775813 4.98828747004786 0.06014703638606
H -0.36812665029323 6.29575138384689 -1.21042012046138
N 2.23479858948478 5.75630110518519 -1.41317457050551
H 2.67713100239108 3.56192658087998 0.01657727342879
H -2.00496415082586 3.27733051760931 2.44305321338362
H -2.59096723949817 2.66682015945875 0.89562455861411
H 0.99959561607418 2.25673036047026 1.29344609526286
O 3.40019980039617 5.38638141062544 -1.35912397701006
O 1.84058260012005 6.71615206368240 -2.06574240096731

14b(6)

125
Coordinates from ORCA-job
C -0.10837035798926 -0.50513652843136 1.52669106573760
N 0.97213059589011 0.29326422714867 1.71515428542224
C 1.41062259303426 0.36461562511346 3.12405365906210
C 0.36326669487827 -0.47970487119968 3.84522446563828
N -0.44233224944749 -1.00643349699084 2.73331456016433
C 1.91198608640441 0.78533233959342 0.75269855907553
H 1.42561691342382 1.40347620568657 3.45699259461851
H 2.42277212610943 -0.03782427793587 3.21864446820934
H -0.26943031847028 0.11165518393473 4.51356011891676
H 0.79905117220169 -1.29950834860587 4.42277373116908
C -1.42287792442901 -2.04379951681284 3.01871937374548
Ru -1.19627742457654 -0.68863836057822 -0.33394227786613
Cl -3.50153063518052 -1.24297397710548 0.77470414426026
Cl -1.30831685846433 1.71694843289872 -0.43595740777274
P -2.64956096074480 -0.76160102896496 -2.42370505994221
C -4.49437704183288 -0.32515130936949 -2.21923819332214
C -5.33915516228066 -0.28562037426044 -3.50478332249589
H -4.84263676919410 -1.15008145401245 -1.59092974413376
C -6.82615088030815 -0.12314336716001 -3.15019284600182
H -5.03102811364938 0.55788970418510 -4.13051159196094
H -5.20648896709246 -1.18470796098213 -4.10234827909462
C -7.07282928892041 1.12522615216596 -2.29557937429631
H -7.42143706153248 -0.08404005518021 -4.06821178794668

H -7.15808004193816 -1.01098403515529 -2.59636328547174
C -6.18046174768605 1.12837558470923 -1.04965920124587
H -6.85353404724344 2.01775303255242 -2.89685199135919
H -8.12930980508476 1.18781823065834 -2.01443394293543
C -4.69997357732466 0.96955485450716 -1.41305165671969
H -6.32216702698778 2.05466382450859 -0.48320350609087
H -6.47017884418591 0.30587648109086 -0.38470127215282
H -4.37890423146355 1.83068482824765 -2.00548852915038
H -4.09225364441742 0.95435552074651 -0.51306833581168
C -2.53923202598842 -2.58729173753781 -2.98477698380823
C -1.94926126325638 0.24740154081712 -3.89040532645715
C -2.37645899041481 1.72254592383521 -3.83873085364869
H -0.87821074625932 0.21233673607039 -3.68284286607361
C -1.61672732165445 2.55315503709062 -4.87956033755543
H -3.44771155786365 1.80075768543643 -4.04329015682471
H -2.20495471869448 2.12356486973247 -2.83958954353678
C -1.75891802619676 1.97041607422626 -6.28922681430812
H -1.97404381249712 3.58745266863807 -4.85559264097701
H -0.55737077097102 2.58600202187039 -4.60236832935157
C -1.35616827856860 0.49241337000557 -6.32264943568964
H -2.80477522507735 2.06349117509786 -6.60998236311189
H -1.15913452364074 2.54288318057546 -7.00363167792183
C -2.14561462866552 -0.33416448615753 -5.29552419294088
H -1.50838705767638 0.07910200487717 -7.32555191388177
H -0.28515202881407 0.40223067987478 -6.10407082111690
H -3.20716273796115 -0.32867904869312 -5.56509648470573
H -1.80591492266571 -1.37134677548328 -5.33645009473233
C -3.58200620467231 -3.10815681386641 -3.98455838342276
H -1.55000150861493 -2.62087780607502 -3.45887411116347
C -3.23494275125507 -4.53322346574297 -4.43797787624411
H -4.56279971872108 -3.12098450711250 -3.49730487281889
H -3.66662885473018 -2.45604136031957 -4.85236656557061
C -3.13319459729166 -5.48673724190785 -3.24450633340593
H -3.98666229267008 -4.88900066060042 -5.15024732910523
H -2.27595735670448 -4.51495238804699 -4.97115492278364
C -2.14819473169381 -4.95291036918264 -2.19961317126452
H -4.12412354902307 -5.59439438447332 -2.78507900329332
H -2.82925464175186 -6.48505641689800 -3.57299301499524
C -2.50067356873985 -3.52493785092456 -1.76820400097811
H -2.12358223976892 -5.61916297240275 -1.33554764988475
H -1.13728291144834 -4.95228783082925 -2.62379491976781
H -3.46556352228757 -3.49981497042816 -1.25284934663901
H -1.77639181951088 -3.16925089288114 -1.03018980878267
C 0.32178380191112 -0.76536946147787 -1.45249810204184
C 0.95073868345473 0.24118069556138 -2.30536617541640
C 1.89100087050383 -0.31669928345352 -3.12454019561567
C 1.92433770298387 -1.78236130996966 -2.86295858699003
C 1.00904980602285 -2.04214326289978 -1.81521147234816
C 2.62225472548346 -2.82723191034468 -3.44904169686606
C 2.75127080105887 0.40432716179066 -4.06109416731589
H 0.72790474464373 1.29225764185688 -2.21842056787691
C 4.04260758822334 -0.04815004882609 -4.37833482445568

C 4.85646848926091 0.66973543269586 -5.24790124599672
C 4.40127344978425 1.85411835035889 -5.82193709726530
C 3.12544331656165 2.32326234574814 -5.50784198519991
C 2.31325652134518 1.60786934845240 -4.63840700891750
H 4.42583649357707 -0.94240287885405 -3.90812653152391
H 5.85374514057685 0.30790582341889 -5.47028763329062
H 5.03507082157450 2.40970971691114 -6.50359441444434
H 2.76217498231493 3.24621318666734 -5.94510269772841
H 1.32329664406529 1.97220509053381 -4.40270679487538
C 2.45640212771278 -4.12734328526554 -2.93992669943077
H 3.27873846434462 -2.66500843711258 -4.29427216841869
H 3.00707745706809 -4.94707084290436 -3.38668314608378
C 1.60332587323472 -4.37344192242954 -1.87102466510638
C 0.86247833490451 -3.32206589931699 -1.31235758944773
H 1.50569151425003 -5.37592798603984 -1.47094630006745
H 0.19690440319200 -3.51641636129208 -0.48361051165069
C 1.96227947182474 2.15581932254974 0.46213922528995
C 2.93759713598529 2.60087696065005 -0.43062297186799
C 3.88612324068898 1.74109910804209 -0.98325672305467
C 3.85827320956622 0.39910466211748 -0.60651626408237
C 2.89240050533783 -0.09600894761717 0.26794180268030
C 1.07516010703327 3.15634891375240 1.15448762557168
H 2.96757474065644 3.65703334935629 -0.68225212481896
C 4.91920621599971 2.26543630069227 -1.94644204354521
H 4.60347889899984 -0.28160799424139 -1.00485502630369
C 2.95048936952655 -1.53308649281212 0.71876842360113
H 0.74392481715971 3.92653083475279 0.45653051779148
H 0.18566218714915 2.69193199675085 1.57014584461616
H 1.63555736594337 3.65193658979087 1.95677836117552
H 5.44216813425437 3.13011446270527 -1.52703811551849
H 4.45298339281435 2.58402563729255 -2.88248172320694
H 5.65883354085378 1.50265347139903 -2.19164622175200
H 1.96350711426828 -1.98573437353268 0.76788004322249
H 3.55336528632764 -2.12985257062821 0.03434325769910
H 3.40729033314150 -1.60499521578318 1.71397152014164
C -1.08026391693023 -3.37192667647770 2.38366968231274
C 0.24394774376617 -3.81362559477022 2.28634661740593
C 0.54761380174431 -5.02774098243775 1.68738853787303
C -0.49670805342035 -5.80439723963203 1.19634456526132
C -1.82493527773950 -5.41043527649250 1.31422592578217
C -2.10958398683406 -4.18357761454092 1.89805625468478
H 1.04919179354113 -3.19223226997081 2.65609499425596
H 1.56618206163729 -5.37097813304941 1.57471170680678
N -0.18368066617271 -7.05427091755337 0.48546531013050
H -2.60619314561862 -6.04680044450950 0.92495331785697
H -2.40985787618506 -1.72503892020936 2.69063284701347
H -1.44668211018956 -2.16222852224600 4.10597288511854
H -3.12842210818517 -3.82168905081119 1.93435473510159
O 0.99744765244592 -7.30299471800203 0.26284436207824
O -1.11872803319127 -7.76560381192968 0.14202785556677
